# Dietary Habits and Risk of Early-Onset Dementia in an Italian Case-Control Study

**DOI:** 10.3390/nu12123682

**Published:** 2020-11-29

**Authors:** Tommaso Filippini, Giorgia Adani, Marcella Malavolti, Caterina Garuti, Silvia Cilloni, Giulia Vinceti, Giovanna Zamboni, Manuela Tondelli, Chiara Galli, Manuela Costa, Annalisa Chiari, Marco Vinceti

**Affiliations:** 1 Environmental, Genetic and Nutritional Epidemiology Research Center (CREAGEN), Department of Biomedical, Metabolic and Neural Sciences, University of Modena and Reggio Emilia, 41125 Modena, Italy; tommaso.filippini@unimore.it (T.F.); giorgia.adani@unimore.it (G.A.); marcella.malavolti@unimore.it (M.M.); caterina.garuti@gmail.com (C.G.); silvia.cilloni@unimore.it (S.C.); 2 Center for Neurosciences and Neurotechnology, Department of Biomedical, Metabolic, and Neural Sciences, University of Modena and Reggio Emilia, 41126 Modena, Italy; giulia.vinceti@unimore.it (G.V.); giovanna.zamboni@unimore.it (G.Z.); 3 Neurology Unit, Modena Policlinico-University Hospital, 41126 Modena, Italy; manuelatondelli@gmail.com (M.T.); ch.galli@ausl.mo.it (C.G.); chiari.annalisa@aou.mo.it (A.C.); 4 Primary care Department, Modena Local Health Authority, 41124 Modena, Italy; 5 Department of Neuroscience, Psychology, Pharmacology and Child Health (NeuroFARBA), University of Florence, 50139 Florence, Italy; 6 Neurology Unit of Carpi Hospital, Modena Local Health Authority, 41012 Carpi, Italy; m.costa@ausl.mo.it; 7 Department of Epidemiology, Boston University School of Public Health, Boston, MA 02118, USA

**Keywords:** early-onset dementia, dietary habits, MIND diet, DASH diet, Mediterranean diet, risk, prevention

## Abstract

Risk of early-onset dementia (EOD) might be modified by environmental factors and lifestyles, including diet. The aim of this study is to evaluate the association between dietary habits and EOD risk. We recruited 54 newly-diagnosed EOD patients in Modena (Northern Italy) and 54 caregivers as controls. We investigated dietary habits through a food frequency questionnaire, assessing both food intake and adherence to dietary patterns, namely the Greek-Mediterranean, the Dietary Approaches to Stop Hypertension (DASH), and the Mediterranean-DASH Intervention for Neurodegenerative Delay (MIND) diets. We modeled the relation between dietary factors and risk using the restricted cubic spline regression analysis. Cereal intake showed a U-shaped relation with EOD, with risk increasing above 350 g/day. A high intake (>400 g/day) of dairy products was also associated with excess risk. Although overall fish and seafood consumption showed no association with EOD risk, we found a U-shaped relation with preserved/tinned fish, and an inverse relation with other fish. Similarly, vegetables (especially leafy) showed a strong inverse association above 100 g/day, as did citrus and dry fruits. Overall, sweet consumption was not associated with EOD risk, while dry cake and ice-cream showed a positive relation and chocolate products an inverse one. For beverages, we found no relation with EOD risk apart from a U-shaped relation for coffee consumption. Concerning dietary patterns, EOD risk linearly decreased with the increasing adherence to the MIND pattern. On the other hand, an inverse association for the Greek-Mediterranean and DASH diets emerged only at very high adherence levels. To the best of our knowledge, this is the first study that explores the association between dietary factors and EOD risk, and suggests that adherence to the MIND dietary pattern may decrease such risk.

## 1. Introduction

Dementia is a syndrome, usually of a chronic or progressive nature, characterized by impairment of cognitive functions beyond what might be expected from normal ageing [1,2]. Early-onset dementia (EOD) is a heterogeneous group of cognitive disorders characterized by an onset of dementia symptoms before the age of 65 [3]. Such an age cut-point has not been established based on biological differences between younger and older subjects, but mainly on the socio-economic implications of dementia diagnosis at a younger age [3,4]. Indeed, the main feature of EOD compared to late-onset forms is a higher impact at two levels: First, on affected people, particularly in terms of their social functioning and working life [5]; second, on family members, especially when young children are still present [6].

EOD prevalence has been estimated to range between 38 and 420 cases per 100,000 inhabitants, with an annual incidence between 2.4 and 22.6 new cases per 100,000 inhabitants [4], the most common forms being Alzheimer’s dementia (AD), frontotemporal dementia (FTD), and vascular dementia [7].

Little is known about EOD etiology, not least in comparison with the determinants of late-onset dementia. Genetic mutations apparently only account for a small percentage of EOD, around 10% [8,9]. Therefore, the role of environmental factors and modifiable lifestyles including diet seems particularly relevant [10,11,12,13,14]. Among the dietary habits involved, a high consumption of vegetables, fruit, and fish [15,16,17], and adherence to the Mediterranean diet or other dietary patterns (e.g., the Dietary Approaches to Stop Hypertension (DASH) and the Mediterranean-DASH Intervention for Neurodegenerative Delay (MIND) diet) have been associated with a slower cognitive decline and decreased risk of all-age dementia [12,18,19,20,21]. Interestingly, recent studies have evaluated the correlation between AD brain biomarkers and dietary patterns characterized by a higher intake of fresh fruit and vegetables, whole grains, fish and low-fat dairies, along with a lower intake of sweets, fried potatoes, high-fat dairies, and meat. Data provided evidence of protective effects against the risk of developing AD, suggesting that dietary interventions may play a role in the prevention of cognitive decline [22,23].

In this study, we investigated EOD risk in an Italian population in relation to dietary habits, including food consumption and adherence to dietary patterns.

## 2. Methods

Following approval by the Modena Ethics Committee (*n*. 186/2016), we performed a case-control study on environmental and lifestyle risk factors for EOD in the province of Modena, Northern Italy. We recruited EOD cases from newly-diagnosed patients referred to the Cognitive Neurology Network of Modena province, including the Modena Policlinico-University Hospital Memory Center (Modena, Italy) and the Carpi Hospital Neurology Department (Carpi, Italy), in the period October 2016–2019 [7,24]. Cases are referred to this Network by either Neurology Units, primary care services, or general practitioners through specific pathways activated by the Modena Local Health Authority to identify and care for subjects with dementia. Inclusion criteria encompassed EOD diagnosis, residence in the Modena province, and presence of a reliable caregiver. The diagnosis of EOD subtypes has been established according to the most recent clinical criteria, as previously described [7]. The gene mutation status was not available for all participants since it is not routinely part of the clinical workflow. Among those tested, one subject was a carrier of *SP1* gene mutation. We recruited controls from caregivers of subjects with a diagnosis of early or late-onset dementia referred to the same Cognitive Neurology Network. All subjects provided a written informed consent.

We administered a questionnaire tailored to collect personal characteristics and clinical, occupational, and environmental factors potentially affecting the central nervous system [25], and a detailed food frequency questionnaire (FFQ). The latter is a validated semi-quantitative FFQ developed within the European Prospective Investigation in Cancer (EPIC) project, in a version specifically validated for the population of Northern Italy [26,27]. The EPIC-FFQ was designed to estimate the intake of 188 food items over the previous year in terms of frequency and amount. Photos of serving sizes were also used to assist with proper completion by participants.

Foods and beverages were categorized into major food groups and subgroups based on the common EPIC-SOFT classification, as previously reported in detail [28,29]. The final list of food categories included the following items and subcategories: Cereals and cereal products, meat and meat products, milk and dairy products, eggs, fish and seafood, vegetables, mushrooms, legumes, potatoes, fresh and dry fruits, sweet products, oils and fats, and beverages. We also computed alcohol (ethanol) intake by conversion of all quantities of alcoholic beverages into grams of ethanol per day using a methodology previously described [30]. We also computed scores for three diet quality patterns defined *a priori*: The Greek Mediterranean (GM) diet [31], the Dietary Approaches to Stop Hypertension (DASH) diet [32,33], based on a methodology described elsewhere [34], and the Mediterranean-DASH Intervention for Neurodegenerative Delay (MIND) diet [19,35]. In more detail, the GM diet is based on Mediterranean diet scales [31], and scoring is calculated on median intake levels of nine items: Vegetables, legumes, fruit and nuts, dairy products, cereals, meat and meat products, fish, alcohol, and the monounsaturated/saturated fatty acid ratio [34,36]. The range of possible scores was 0–9, with higher scores indicating higher adherence. The DASH diet was originally designed to reduce blood pressure [32,33], and it has been suggested to be neuroprotective [37]. DASH diet adherence scores were calculated according to previous studies [34,38] based on eight components: Fruits, vegetables, nuts and legumes, low-fat dairy products, whole grains, sodium, sweetened beverages, red and processed meats. Overall, possible scores ranged from 8 to 40, with higher scores indicating higher adherence. Eventually, the MIND diet was developed as a hybrid of the Mediterranean and DASH diets associated with slower cognitive decline and decreased incidence of Alzheimer’s dementia [19,35]. MIND diet scores were based on the intake of 15 items, namely whole grains, green leafy and other vegetables, berries, red meat, poultry, fish, legumes, nuts, fast/fried food, olive oil and other fats, cheese, sweets and alcohol/wine. Scores ranged from 0 to 15, with higher scores meaning higher adherence.

In data analysis, we used a multivariable unconditional logistic regression model to estimate the EOD risk associated with dietary factors and patterns. We performed an analysis on the overall population for risk of EOD, also performing a stratified analysis according to the type of diagnosis, namely early-onset Alzheimer’s dementia (EO-AD) and early-onset frontotemporal dementia spectrum (EO-FTD). Regarding dietary factors, we calculated the odds ratio (ORs) and 95% confidence intervals (CIs) according to the increasing tertiles based on the distribution in the control group using the lowest tertile as a reference category. We also modeled the relation between dietary factors and EOD risk using the restricted cubic spline model with three knots (10, 50, and 90 percentiles). We included in the multivariable model as potential confounders and effect-modifiers sex, age, educational attainment (as years of education), and total energy intake (kcal/day). We used “logit”, “mkspline”, and “xblc” routines of the Stata-16.1 statistical package (Stata Corp., College Station, TX, USA, 2020) for statistical analyses.

## 3. Results

### 3.1. Characteristics of the Study Population

Of the 150 eligible participants, only 144 could be contacted. We recruited 112 subjects, 58 EOD cases, and 54 controls with an average response rate of 78%. Reasons for non-participation were unwillingness to contribute to the research and lack of time to fill out the questionnaire. In addition, four cases were excluded as they did not return a reliable and complete FFQ, leaving 54 cases and 54 controls for the final analysis. Table 1 reports the characteristics of the study participants. The average age at the questionnaire filling date was 65 years (66 for EOD cases and 64 years for referents), with a higher proportion of women (57%). The mean age of EOD onset was 59.8 years (59.7 years for EO-AD and 59.8 for EO-FTD), ranging from 45 to 65 years. Alzheimer’s dementia (EO-AD) was the most frequent diagnosis (*n* = 30), followed by frontotemporal dementia spectrum disorders (EO-FTD, *n* = 18), vascular dementia (*n* = 4), or other rarer diseases (Supplemental Table S1).

### 3.2. Assessment of Dietary Habits

Supplemental Tables S2 and 3 show the average intake of food and beverages for the study participants. No subjects reported a special dietary regimen for medical purposes. The total energy intake was slightly higher in cases compared to controls. In regards to cereal products, cases were shown to have a higher intake compared to controls, in particular EO-FTD subjects. Cases had a slightly higher intake of meat products compared with controls, including red and white meat, and a higher intake of dairy products, particularly milk and yogurt, especially in EO-AD compared to EO-FTD cases. Cases also showed a substantially similar intake of fish and seafood, but a higher consumption of preserved and tinned fish particularly for EO-FTD cases, a lower intake of piscivorous fish and crustaceans/molluscs, especially in EO-FTD cases. Cases also showed a lower intake of overall vegetables, with similar results for all vegetable types but cabbage, and a lower intake of potatoes and legumes. Overall, fresh fruit intake was somewhat higher in cases than controls, although the former showed a lower intake of citrus fruits yet a higher intake of all other fruits, with similar results across EO-AD and EO-FTD. Dry fruit intake was lower in cases than in controls, particularly for nuts and seeds and especially in EO-FTD, characterized by a much lower intake of both overall dry fruit, nuts, and seeds. Sweet intake was higher in cases, particularly due to a higher consumption of ice-cream, biscuits, and dry cakes, while consumption of chocolate-based products was lower. Cases consumed less oils and fats, with a lower intake of both vegetable oils and olive oil. Concerning beverages, cases had a lower intake of coffee and tea, but results were the opposite in the subgroup analysis according to an EOD diagnosis, with a higher intake in EO-AD cases and a much lower one in EO-FTD cases, who showed very low tea consumption. Wine consumption was substantially similar in cases and controls, with EO-AD cases reporting a lower intake and EO-FTD a higher one, mainly due to the higher consumption of white wine. A higher intake of aperitif wines and beers was reported for controls. The overall alcohol intake was comparable in cases and controls, although EO-FTD cases showed a much higher intake compared to EO-AD cases. Concerning non-alcoholic beverages, cases reported a higher intake of fruit juices and a lower consumption of soft drinks.

### 3.3. Assessment of the Relation between Dietary Habits and Dementia Risk

In Supplemental Table S4, we report risk estimates associated with increasing tertiles of food and beverage intake. Overall, about cereals products, we found an inverse association with EOD risk, although a positive association can be noted for subjects in the third tertile for both pasta and rice consumption. For meat products, we found a direct association with EOD, with a higher risk in the second tertile compared to the third one. In the subgroup analyses, however, red, white, and processed meat showed a very imprecise inverse association in the second tertile, but a null/positive association in the third tertile. On the other hand, offal intake seemed inversely associated with EOD risk, especially for subjects in the third tertile. Milk and dairy products were in general positively associated with EOD, particularly in the third tertile of exposure. Both piscivorous and non-piscivorous fish seemed inversely associated with EOD risk. For preserved and tinned fish, crustaceans and molluscs, conversely, we found an inverse association in the second tertile, but a positive one in the third tertile. In general, vegetables were inversely associated with EOD risk, particularly leafy, root, and other vegetables as well as mushrooms and potatoes, and an almost substantial null association for legumes. In regards to fruits, we found an inverse association for fresh fruits, especially citrus fruits, as well as dry fruits. Conversely, the intake of all other fruits showed a negative association in the second tertile and a positive one in the third tertile. For overall sweets, we found a positive association with EOD risk, mainly due to ice-cream, cakes, pies/pastries, and biscuits/dry cakes, while chocolate and other (non-chocolate) confectionery showed an inverse association. Overall, oils and fats showed an inverse association, mainly due to the intake of olive and non-olive oils, while we found a positive association with butter and other animal fats. Concerning beverages, coffee and tea, aperitif wines/beers, and spirits/liqueurs showed an inverse association with EOD risk. Red wine showed an inverse association for subjects in the second tertile, and a positive one in the third tertile. Conversely, white wine and soft drinks showed a higher risk in the second tertile and lower one in the third tertile. Finally, fruit juices seemed positively associated with disease risk, especially in the second tertile.

In general, we found similar results in the analysis based on the EOD diagnosis, with a few exceptions (Supplemental Table S4). Bread intake was inversely associated with EOD risk and this was also true for EO-AD. However, a direct/null association is present in EO-FDT. Fresh cheese intake showed a positive association with EO-FTD, but a null association for EO-AD. Conversely, aged cheese intake did not show such an association with EO-FTD and a slight positive association emerged for EO-AD in the third tertile of exposure. With regard to fish intake, EO-FTD showed a positive association with preserved and tinned fish, while an inverse one can be noted for EO-AD. In regards to fresh fruit intake, both EO-AD and EO-FTD showed an inverse association, but we found a positive association for citrus fruit in EO-FTD and a negative one in EO-AD. In relation to sweet intake, the increased risk for biscuits and dry cakes was confirmed in EO-AD, while we found a decreased risk for EO-FTD. Finally, alcohol intake was associated with a slightly increased risk in EO-FTD, but not in EO-AD.

### 3.4. Assessment of the Relation between Dietary Patterns and Dementia Risk

Moving on to the analysis of adherence to the investigated dietary patterns, cases (overall and both EO-AD and EO-FTD) generally showed slightly lower mean scores (Table 2). In the risk analysis according to the tertile distribution (Table 3), we found a lower EOD risk at increasing adherence in all three dietary patterns, especially for high adherence to MIND dietary patterns.

**Figure 1:**
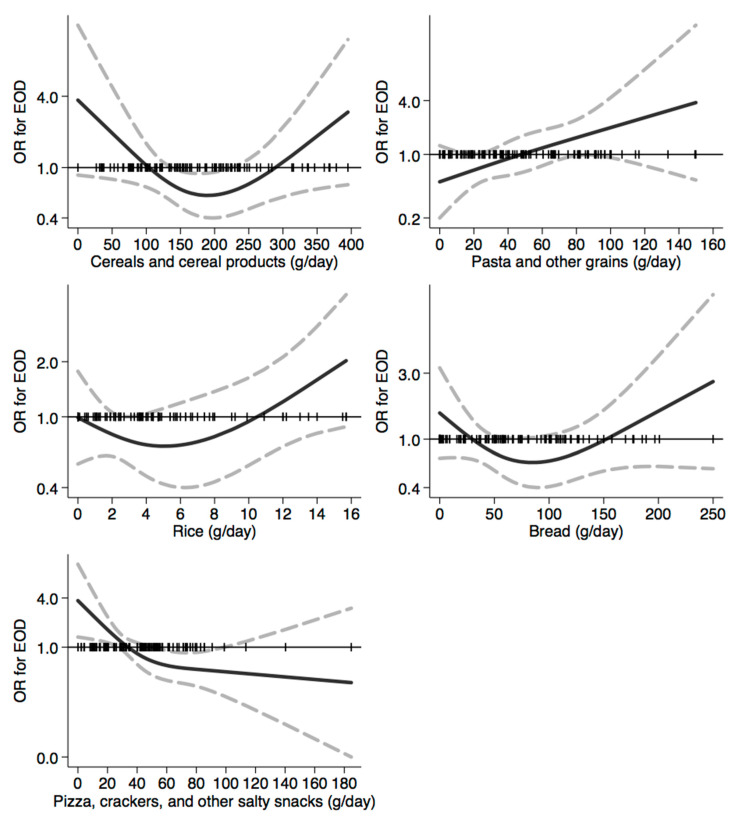
Spline regression analysis of early-onset dementia (EOD) risk for increasing intake of cereals and cereal products. The black line indicates odds ratios for dementia risk; dash gray lines are 95% confidence limits; the reference line at 1.0 with black spikes indicates the distribution of participant intake.

**Figure 2:**
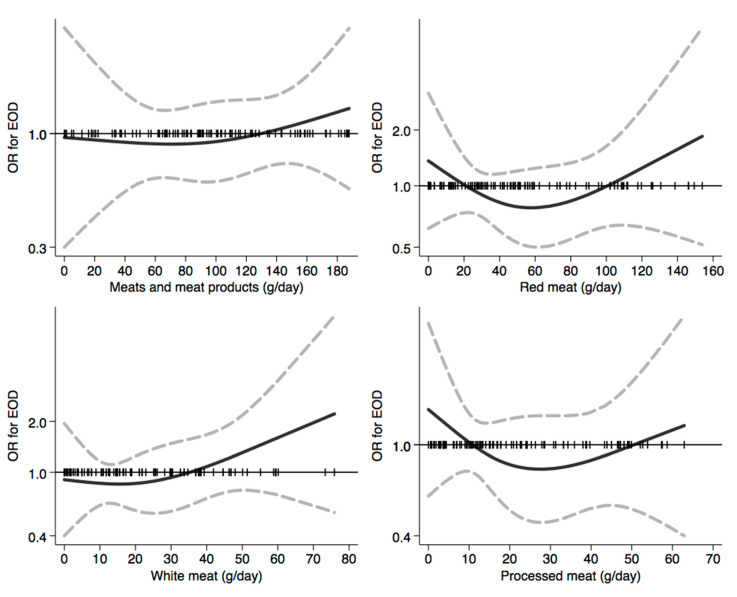
Spline regression analysis of early-onset dementia (EOD) risk for increasing intake of meats and meat products. The black line indicates odds ratios for dementia risk; dash gray lines are 95% confidence limits; the reference line at 1.0 with black spikes indicates the distribution of participant intake. Note: spline analysis was not possible for offal due to a few subjects reporting consumption which is different from a null value.

**Figure 3:**
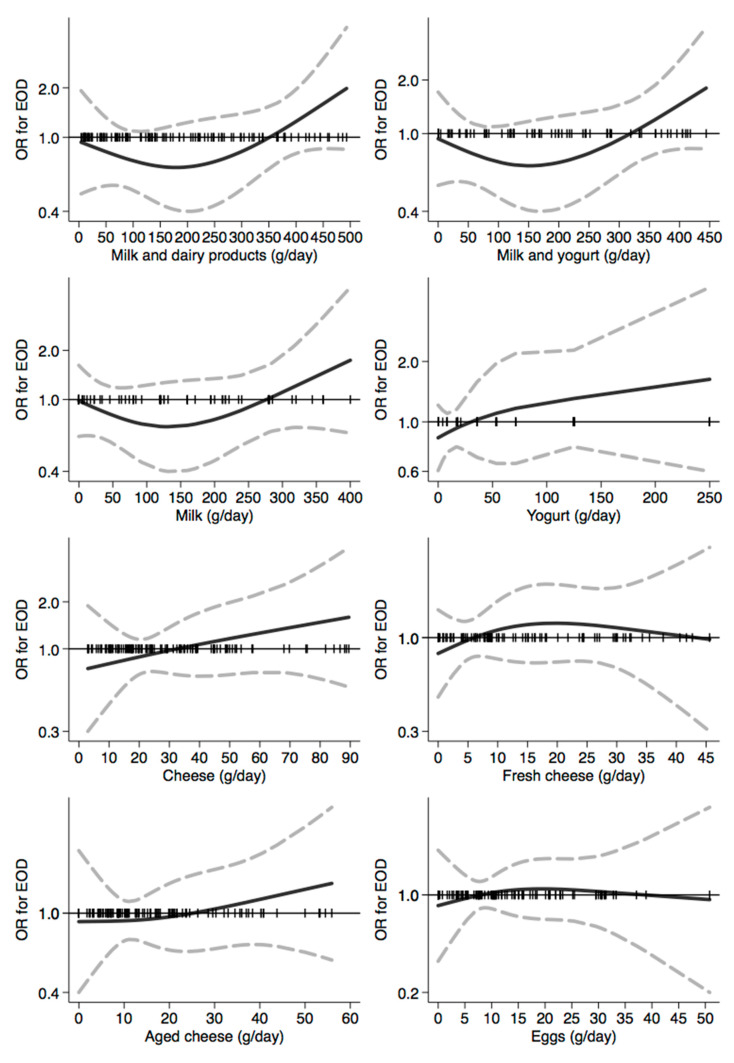
Spline regression analysis of early-onset dementia (EOD) risk for increasing intake of milk, dairy products, and eggs. The black line indicates odds ratios for dementia risk; dash gray lines are 95% confidence limits; the reference line at 1.0 with black spikes indicates the distribution of participant intake.

**Figure 4:**
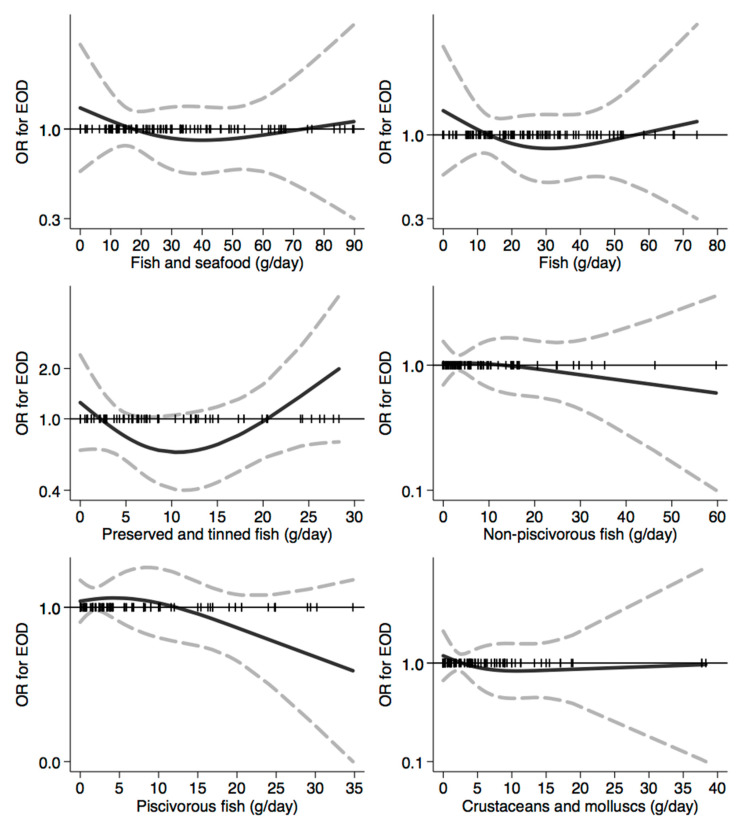
Spline regression analysis of early-onset dementia (EOD) risk for increasing intake of fish and seafood. The black line indicates odds ratios for dementia risk; dash gray lines are 95% confidence limits; the reference line at 1.0 with black spikes indicates the distribution of participant intake.

**Figure 5:**
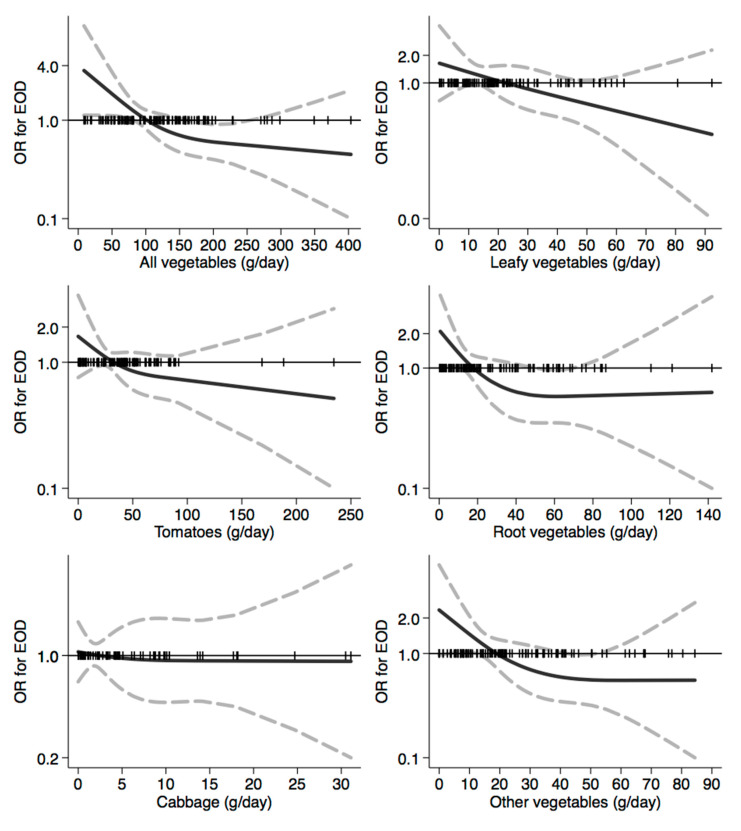
Spline regression analysis of early-onset dementia (EOD) risk for increasing intake of vegetables. The black line indicates odds ratios for dementia risk; dash gray lines are 95% confidence limits; the reference line at 1.0 with black spikes indicates the distribution of participant intake.

**Figure 6:**
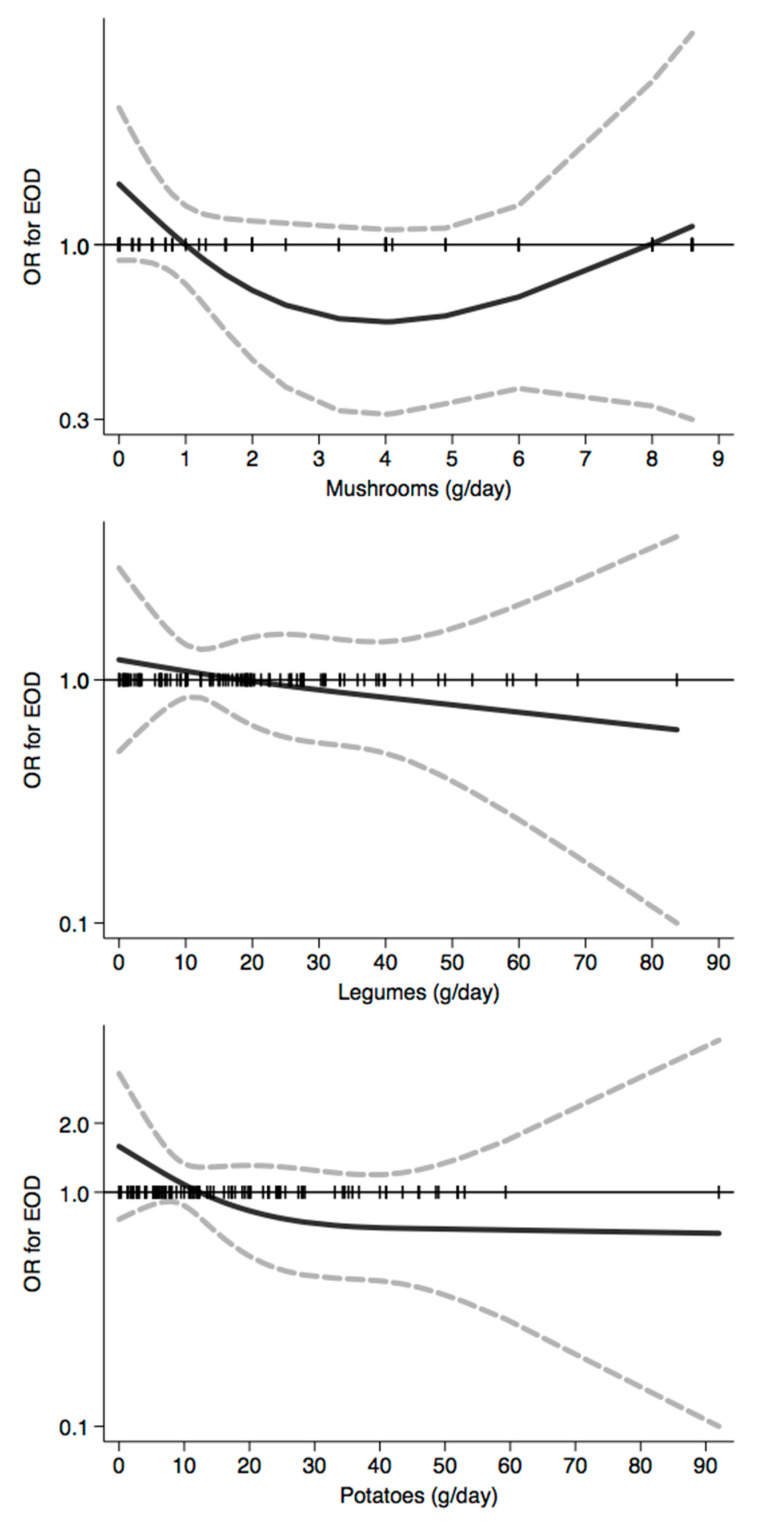
Spline regression analysis of early-onset dementia (EOD) risk for increasing intake of mushrooms, legumes, and potatoes. The black line indicates odds ratios for dementia risk; dash gray lines are 95% confidence limits; the reference line at 1.0 with black spikes indicates the distribution of participant intake.

**Figure 7:**
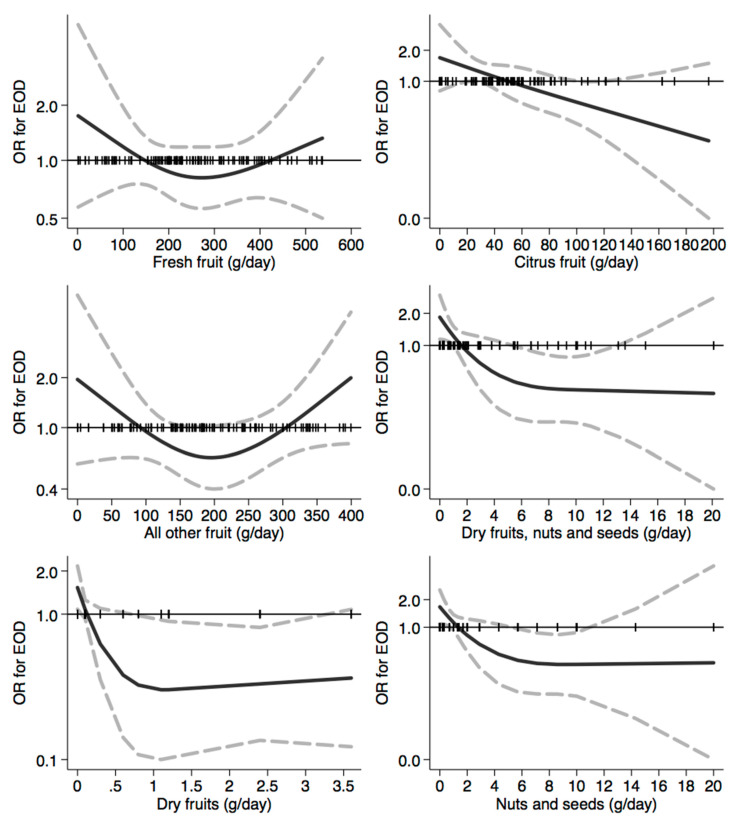
Spline regression analysis of early-onset dementia (EOD) risk for increasing intake of fresh and dry fruits. The black line indicates odds ratios for dementia risk; dash gray lines are 95% confidence limits; the reference line at 1.0 with black spikes indicates the distribution of participant intake.

**Figure 8:**
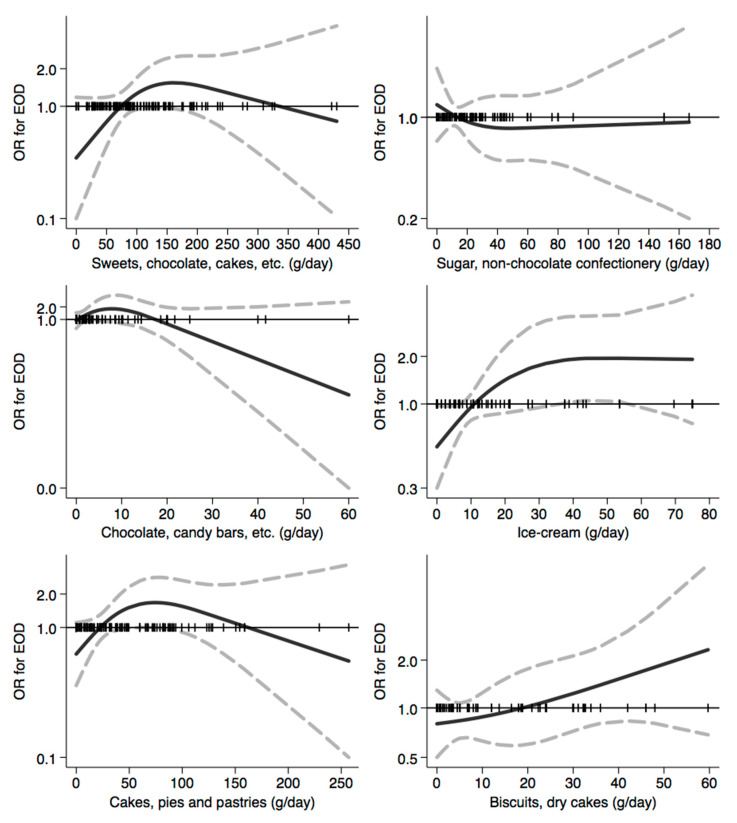
Spline regression analysis of early-onset dementia (EOD) risk for increasing intake of sweets products. The black line indicates odds ratios for dementia risk; dash gray lines are 95% confidence limits; the reference line at 1.0 with black spikes indicates the distribution of participant intake.

**Figure 9:**
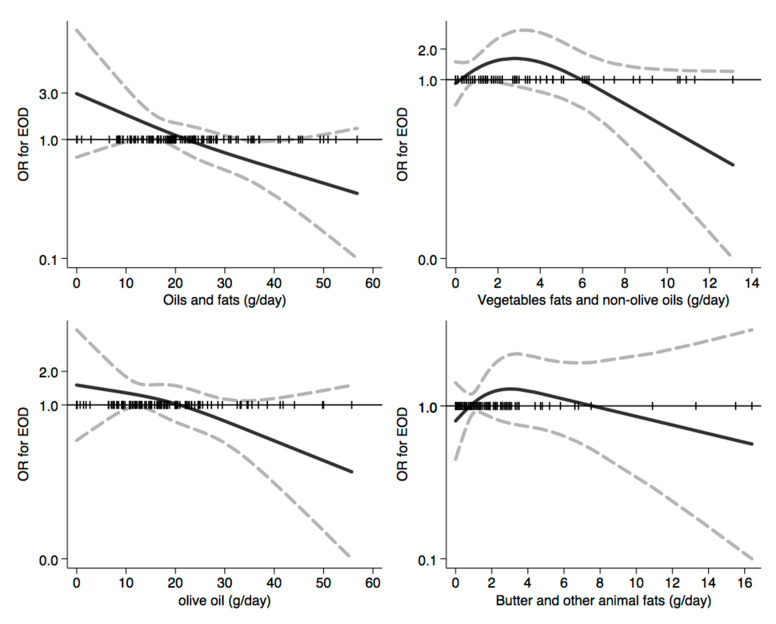
Spline regression analysis of early-onset dementia (EOD) risk for increasing intake of oils and fats. The black line indicates odds ratios for dementia risk; dash gray lines are 95% confidence limits; the reference line at 1.0 with black spikes indicates the distribution of participant intake.

**Figure 10:**
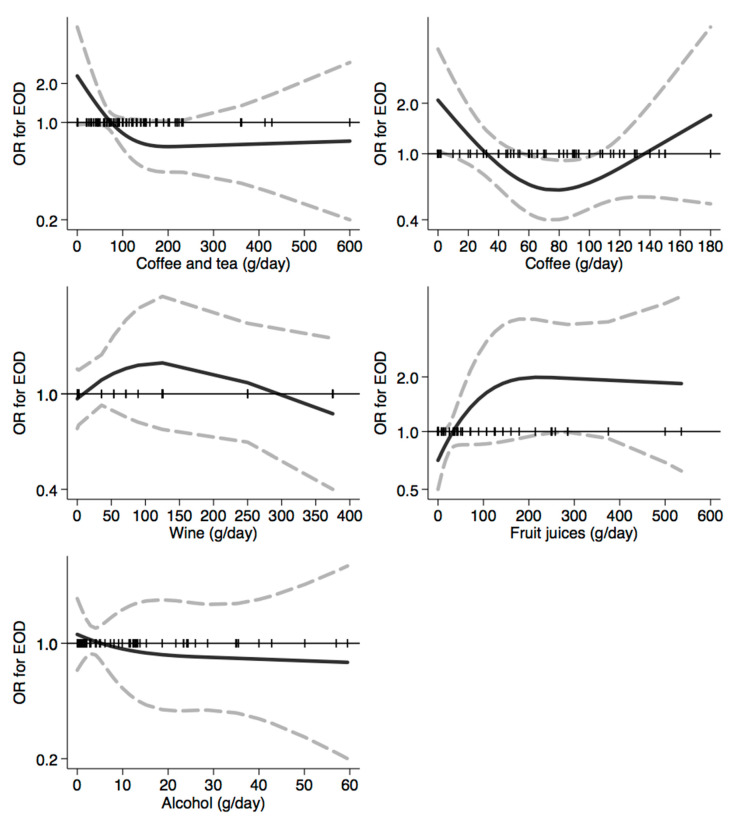
Spline regression analysis of early-onset dementia (EOD) risk for increasing intake of beverages. The black line indicates odds ratios for dementia risk; dash gray lines are 95% confidence limits; the reference line at 1.0 with black spikes indicates the distribution of participant intake. Note: Spline analysis was not possible for most of the beverages (namely tea, red, white and aperitif wines and beers, spirits and soft drinks) due to a few subjects reporting consumption which is different from a null value.

In Figure 1, Figure 2, Figure 3, Figure 4, Figure 5, Figure 6, Figure 7, Figure 8, Figure 9 and Figure 10, we present data based on the spline regression analysis adjusted for sex, age, educational attainment, and total energy intake. Due to the very high number of non-consumers, the spline analysis was not feasible for some offal and several beverages, namely tea, red and white wine, aperitif wines and beers, and soft drinks. Overall, cereal products showed a U-shaped relation with EOD, with a lower risk at around 200 g/day, yet a higher one above 350 g/day (Figure 1). A similar relation was found for bread and rice intake. Conversely, pasta and other grains showed a linear relation with decreased risk at low intake levels and an increasing one from 50–60 g/day. On the other hand, pizza, crackers, and other salty snacks showed increased risk in the case of null exposure, with decreased risk from 40 g/day after which a plateau was reached. Overall meat consumption did not seem to be associated with EOD (Figure 2), although an increase in risk can be noted for a high intake of both red (>100/120 g/day) and white meat (>40/50 g/day). In regards to dairy products, we found null/decreased risk for all products, except for an increase in risk at very high intake levels, namely >400 g/day for overall dairy products or >350 g/day for milk and yogurt (Figure 3). Similarly, we found null risk in association with the intake of eggs (Figure 3) and overall fish and seafood (Figure 4). Interestingly, we found a U-shaped relation with fish intake, with increased risk for null consumption and above 20 g/day for preserved and tinned fish (Figure 4). Conversely, a high intake (>20 g/day) of other fish, especially piscivorous fish, appeared to the decreased EOD risk. All vegetables showed an inverse association with EOD, with increased risk in the case of null intake and decreasing risk starting from 100 g/day, above which a plateau seems to have been reached (Figure 5). Such pattern of association can also be noted in all vegetable subgroups, especially leafy vegetables showing a slight continuous decrease, while cabbages showed a null association at all intake levels. Mushrooms showed a U-shape relation, with a lower risk at around 4 g/day. On the other hand, we found a slight inverse association for potatoes and legumes, albeit a very imprecise one (Figure 6). In spite of the substantially linear inverse correlation of citrus fruit intake with EOD risk, we found a U-shaped relation with fresh fruit and all other fruit intake, with a lower risk at approximately 250 and 200 g/day, respectively (Figure 7). Conversely, dry fruit intake showed an increased risk for subjects reporting null consumption and a decreased risk from 4 g/day of dry fruits and nuts and from 1 g/day of dry fruits, above which a plateau was reached (Figure 7). Sweet products showed an inverse-U relation with EOD risk, mainly driven by the lower risk associated with a high intake of chocolate and other chocolate-based products (Figure 8). However, sugar and other confectionery showed a null association, while ice-cream, biscuits, and dry cakes showed a positive correlation, with null risk for non-consumers and an increased risk for intake above 20–30 g/day for both ice-cream and dry cakes, respectively (Figure 8). In general, oils and fats showed that a linear inverse association with increased risk starting from null intake, soon reversed above 20 g/day especially for olive oil and other vegetable oils and fats (Figure 9). For beverages, we found a U-shaped relation with coffee consumption, with a lower risk at 70 g/day (Figure 10). Conversely, we found almost a null relation with wine and alcohol intake, while risk increased with fruit juice consumption exceeding 100 g/day (Figure 10).

Spline regression analyses stratified by the EOD clinical type are reported in Supplemental Figures S1–S10 for EO-AD and in Supplemental Figures S11–S20 for EO-FTD. We generally found comparable results across EOD forms. Nonetheless, there was evidence of a U-shaped relation between red meat intake and EO-AD (Supplemental Figure S2), and between processed meat and EO-FTD (Supplemental Figure S12), with the lowest risk ratio occurring at 60 and at 25 g/day, respectively. In regards to dairy products, the shape of the relation with cheese intake seemed opposite for EO-AD and EO-FTD, with an inverse U-shaped relation for EO-AD, especially for fresh-cheese intake (Supplemental Figure S3), and a U-shaped relation for EO-FTD, especially for overall cheese intake (Supplemental Figure S13). Moreover, egg intake seemed to establish an inverse-U relation for EO-AD only (Supplemental Figure S3). In addition, mushroom intake showed a linear inverse association for EO-AD (Supplemental Figure S6), and a U-shaped one with EO-FTD (Supplemental Figure S16).

**Figure 11:**
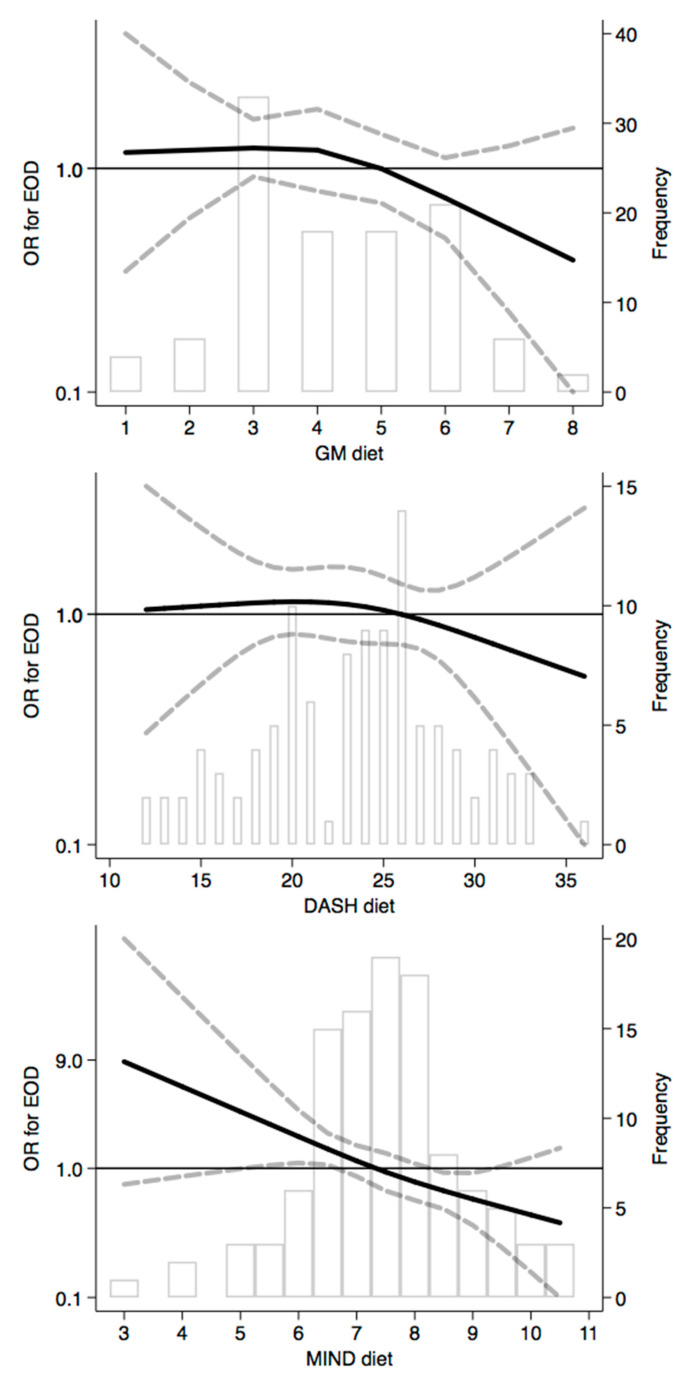
Spline regression analysis for dietary patterns. Greek-Mediterranean (GM) diet, Dietary Approaches to Stop Hypertension (DASH) diet, and Mediterranean-DASH Intervention for Neurodegenerative Delay (MIND) diet. The black line indicates odds ratios for dementia risk; dash gray lines are 95% confidence limits; the reference line at 1.0 with gray bars shows the dietary-pattern score distribution.

The spline regression analysis carried out for dietary pattern adherence showed no substantial association between EOD risk and both GM and DASH diets, with a reduction in risk from a score of ≥6 and ≥28, respectively (Figure 11). Conversely, a substantially linear inverse association emerged for the MIND diet, with a higher risk at very low adherence and decreasing risk above a total score around 8/9. We found comparable results in the analyses performed by the EOD subtype (Supplemental Figures S21 and 22), with the exception of adherence to the DASH diet. This dietary pattern showed an inverse U-shaped relation with EO-FTD risk, with a decreased risk for subjects at very low and very high adherence levels (Supplemental Figure S22), while no association emerged for EO-AD (Supplemental Figure S21). 

## 4. Discussion

In this study, we found detrimental effects of consumption of high amounts of cereals, dairy products, and some types of sweets on EOD risk, while the intake of vegetables, dry fruits, and chocolate appeared to be beneficial, as was a specific dietary pattern, the MIND diet.

The role of dietary habits on cognitive decline has been investigated in previous studies [39], but the real association between diet and dementia, along with the mechanisms involved in beneficial and detrimental effects, are still unclear and thoroughly debated. In addition, it should be noted that previous studies were generally performed among individuals with late-onset dementia, while the present study specifically focused on younger subjects. Consistent with previous findings, we found a protective effect on EOD particularly with a higher consumption of leafy vegetables and fresh fruit. This protective effect on cognitive decline could be linked to the consumption of foods rich in bioactive substances such as polyphenols, highly present in fruits, vegetables, cereals, coffee/tea, cacao, and wine [40,41,42]. In the studies suggesting that vegetable intake showed an inverse association with cognitive decline [43,44], an apparent beneficial role of consumption of leafy and root vegetables has been reported [17,45]. Conversely, fruit intake showed contrasting results with null [17,44] or an inverse association [42], with the latter mainly emerging from the analysis evaluating dry fruit intake [17,40,46].

Similarly, the intake of omega-3 fatty acids due to fish consumption has been linked to improved cognition capacities [41,43,47,48,49,50]. Interestingly, we found a null association when overall fish and seafood intake was considered, whereas a slight decrease in risk could be found when considering fish, particularly fresh fish, only. The association we detected between a high daily intake of preserved and tinned fish and EOD is of interest, since such an intake in Italy is mainly due to the consumption of canned tuna [51], a source of heavy metals such as mercury, lead, cadmium, and a metalloid of potential neurotoxicity such as selenium [51,52,53,54,55,56].

Contrasting results with detrimental [57,58] or null effects [31,59] have been reported for dairy products. However, a preventive role has not yet been entirely ruled out, especially considering the different types of dairy products [60,61]. In particular, we found a dose-dependent association between dairy products and EOD risk. In addition, a null to negative association was observed for subjects reporting an intake of up to 350 g/day, when EOD risk starts to increase, thus suggesting adverse effects only at very high intake levels. A possible beneficial role in the cognitive function of substances released during fermentation processes, such as oleamide and dehydroergosterol, has been suggested, following suppression of microglial inflammation, as well as promotion of synaptic extension and neuronal survival [62].

A high cereal intake seems associated with a sharper cognitive decline [58]. In our study, we found an optimum cereal intake at approximately 200 g/day with reference to EOD risk. This is not surprising in that cereals (particularly bread) are fundamental components of the Mediterranean diet [63]. This mainly relates to a beneficial effect of whole grain consumption [64], possibly due to high concentrations of dietary fiber, resistant starch, and oligosaccharides, as well as several phytochemicals (phytates and phenolic compounds) characterized by antioxidant activities [65].

Sweets are generally associated with adverse cognitive effects [66,67]. Possible biological mechanisms of that association may involve generally high fat contents, especially high in saturated and trans-fats and lower in polyunsaturated and monounsaturated fats leading to blood brain barrier dysfunction and increased amyloid beta protein aggregation [68]. However, we observed an indication of beneficial effects from moderate consumption of chocolate products, consistent with previous studies [69,70]. Such beneficial effects, if real, may be due to the cocoa polyphenol intake [71] that might slow MCI progression to dementia [72].

We found a clear inverse association between coffee consumption and EOD risk. A recent dose-response meta-analysis of prospective studies [73] showed a statistically imprecise U-shaped relation between coffee intake and all-cause dementia risk, with the lowest dementia risk at two cups/day (RR = 0.90, 95% CI 0.95 to 1.08) and increased risk at and above five cups/day (RR = 1.11, 95% CI 0.94 to 1.30). Estimate imprecision might be due to the heterogeneity of exposure assessment methods (i.e., cups), which may correspond to different coffee amounts depending on country. Nonetheless, we also found a U-shaped association with a lowest risk at 70 g/day, corresponding to approximately two small (i.e., ‘espresso type’) cups, according to typically Italian habits as assessed in the EPIC-FFQ [74,75]. Contrary to our expectations based on the epidemiologic literature, we found no clear and meaningful relation between wine or alcohol intake and disease risk. No consumption and excessive consumption of wine or alcohol have both been associated with increased dementia risk [76,77]. In particular, a J-shaped relation has been proposed where low-to-moderate intake was associated with reduced risk, while both null and elevated intakes were correlated with increased risk [78,79,80].

Concerning dietary patterns, our results for EOD are fully consistent with previous studies, although these were generally carried out in older individuals, suggesting a beneficial effect on the overall cognitive function at higher adherence levels [19,35,81,82,83,84,85,86]. In particular, we found an indication of protective effects on EOD risk only at high adherence levels to the Greek-Mediterranean Diet (≥6) and DASH index (≥28). On the other hand, the only diet which was strongly and linearly associated with EOD risk was the MIND pattern. This was not entirely unexpected, since the MIND pattern has been shown to better predict an incidence of cognitive impairment or cognitive decline as compared to the Mediterranean Diet and DASH index [87,88]. In addition, the MIND diet has shown beneficial effects on other neurological diseases [89,90,91], while a clinical trial about the role of the diet on cognitive decline and brain neurodegeneration is ongoing [92].

The three dietary patterns under investigation have some similarities, since they emphasize natural plant-based foods and limit the intake of animal-derived products and high saturated-fats. Nevertheless, MIND has a distinctive pattern. It was developed as a hybrid of the DASH and Mediterranean diets with modifications, by reflecting the most compelling scientific evidence on foods and nutrients that protect the brain [93]. The MIND pattern attributes beneficial effects to the intake of cheese (<1 serving/week), green leafy vegetables (≥6 servings/week), berries (>1 serving/week), and fast fried food (<1 time/week) [35,83]. In addition, a high adherence to the investigated dietary patterns is generally associated with high levels of physical activity and other diet-related lifestyle factors [63] having beneficial effects on cognitive function [94,95]. These factors were not taken into account in our study. They might have contributed to a lower EOD risk and to masking the effect of the diet, which becomes evident only at high adherence levels.

To the best of our knowledge, our study is the first to investigate the role of dietary factors on early-onset dementia risk. In fact, previous studies focused on late-onset dementia or did not implement a specific restriction to EOD cases. The use of the validated EPIC-FFQ also allowed for a comprehensive assessment of dietary habits, including the investigation of single food categories and overall dietary-pattern adherence. In addition, we included recently-diagnosed EOD cases, thus limiting the risk of bias related to a long duration of the disease due to disease progression and increasing disability. Another strength of the present study was the ability to apply stratified analyses to the investigation of possible selective associations between diet and EOD risk according to the clinical dementia type.

Some limitations of our study should also be noted. First, the small samples of EOD participants, mainly due to the low incidence of the disease, affected the statistical precision of the risk estimates, especially for those food categories characterized by a large number of non-consumers. As a consequence, such a limited number of subjects hampered our ability to reliably identify weak associations. This was the case especially in subgroup analyses, which suggest caution in the interpretation of estimates characterized by a high imprecision, as well as the need to plan larger studies on the relations between diet and EOD. Secondly, although the EPIC-FFQ was designed and validated to collect dietary information at least over the previous year, we cannot rule out a possible misclassification due to changes of dietary habits of study participants, especially with reference to life-long habits. However, a recent survey comparing adherence to the Mediterranean diet over a long time period (1997–2012) showed similar values for the Emilia Romagna population [96], suggesting a general stability over time of dietary habits. In addition, the use of caregivers as controls could have led to a risk of overmatching and downplayed the real associations between dietary factors and disease risk. As a matter of fact, some controls (particularly cases’ family members) may have shared lifestyle habits including dietary patterns with EOD cases, thus reducing the strength of the associations we estimated. Finally, a genetic status was unavailable for almost all study participants. In particular, no data about the referent population could be retrieved, thus hampering the ability to assess their role in modifying EOD risk in relation to dietary factors.

## 5. Conclusions

This case-control study provides insights on the role of dietary factors in EOD risk. In particular, our findings suggest a detrimental effect on EOD risk due to a high intake of cereals, dairy products, and some types of sweets. Conversely, the intake of some types of fish, vegetables, dry fruits, and chocolate alongside moderate coffee consumption appear to be beneficial. Finally, our study indicates that an increasing adherence to the Mediterranean-DASH Intervention for Neurodegenerative Delay (MIND) may decrease EOD risk.

## Figures and Tables

**Table 1:** Characteristics of the study participants. Early-onset dementia (EOD), early-onset Alzheimer’s dementia (EO-AD), and early-onset frontotemporal dementia spectrum (EO-FTD).

	Controls	All EOD Cases	EO-AD Cases	EO-FTD Cases
	*n* (%)	*n* (%)	*n* (%)	*n* (%)
All subjects	54 (100)	54 (100)	30 (100)	18 (100)
Age at questionnaire filling				
Mean (standard deviation)	63.8 (9.6)	66.2 (4.6)	65.9 (4.5)	66.6 (4.7)
<65 years	28 (51.8)	19 (35.2)	11 (36.7)	5 (27.8)
≥65 years	26 (48.2)	35 (64.8)	19 (63.3)	13 (72.2)
Sex				
Men	23 (42.6)	24 (44.4)	11 (36.7)	10 (55.6)
Women	31 (57.4)	30 (55.6)	19 (63.3)	8 (44.4)
Educational attainment				
Primary or less	11 (20.4)	13 (24.1)	6 (20.0)	5 (27.8)
Middle school	11 (20.4)	20 (37.0)	10 (33.3)	8 (44.4)
High school	21 (38.9)	18 (33.3)	12 (40.0)	4 (22.2)
College or more	11 (20.4)	3 (5.6)	2 (6.7)	1 (5.6)
Marital status				
Married/unmarried partner	48 (88.9)	45 (83.3)	23 (76.7)	16 (88.9)
Single	3 (5.6)	1 (1.9)	1 (3.3)	0 (0.0)
Separated/divorced	2 (3.7)	2 (3.7)	0 (0.0)	2 (11.1)
Widowed	1 (1.9)	6 (11.1)	6 (20.0)	0 (0.0)

**Table 2:** Adherence to dietary patterns (Greek-Mediterranean (GM) diet; DASH: Dietary Approaches to Stop Hypertension (DASH) diet); Mediterranean-DASH Intervention for Neurodegenerative Delay (MIND) diet) in the study population (Early-onset dementia (EOD), early-onset Alzheimer’s dementia (EO-AD), and early-onset frontotemporal dementia spectrum (EO-FTD). Values reported as mean and standard deviation.

Dietary Pattern	Controls	EOD Cases	EO-AD Cases	EO-FTD Cases
GM diet (range 0–9)	4.4 (1.7)	4.1 (1.5)	4.1 (1.5)	4.1 (1.5)
DASH diet (rang 8–40)	23.7 (5.5)	23.1 (5.0)	23.5 (5.4)	23.6 (4.4)
MIND diet (range 0–15)	7.8 (1.3)	7.1 (1.4)	7.2 (1.4)	7.2 (1.3)

**Table 3:** Odds ratio (OR) and 95% confidence intervals (CI) of early-onset dementia (EOD), early-onset Alzheimer’s dementia (EO-AD), and early-onset frontotemporal dementia spectrum (EO-FTD) for increasing tertiles of adherence to dietary patterns. Greek-Mediterranean (GM) diet, Dietary Approaches to Stop Hypertension (DASH) diet, and Mediterranean-DASH Intervention for Neurodegenerative Delay (MIND) diet. A linear trend for 1-unit increase is reported.

				All EOD			EO-AD			EO-FTD
Food Items	Median	Cases/ Controls	OR	(95% CI)	Cases/ Controls	OR	(95% CI)	Cases/ Controls	OR	(95% CI)
**GM diet**										
1st tertile (ref.)	3	24/19	1.00	-	14/19	1.00	-	7/19	1.00	-
2nd tertile	5	18/18	0.76	(0.30–1.96)	10/18	0.73	(0.24–2.17)	6/18	0.87	(0.22–3.35)
3nd tertile	6	12/17	0.45	(0.16–1.26)	6/17	0.40	(0.12–1.35)	5/17	0.60	(0.14–2.61)
Linear trend			0.84	(0.65-1.09)		0.84	(0.62–1.13)		0.83	(0.57–1.20)
**DASH diet**										
1st tertile (ref.)	18	21/19	1.00	-	10/19	1.00	-	7/19	1.00	-
2nd tertile	25	22/19	0.87	(0.35–2.15)	13/19	1.08	(0.37–3.17)	7/19	0.80	(0.22–3.00)
3nd tertile	29	11/16	0.60	(0.21–1.72)	7/16	0.79	(0.23–2.74)	4/16	0.71	(0.14–3.52)
Linear trend			0.98	(0.90–1.06)		0.99	(0.91–1.08)		1.01	(0.89–1.14)
**MIND diet**										
1st tertile (ref.)	6.5	30/16	1.00	-	16/16	1.00	-	9/16	1.00	-
2nd tertile	7.5	15/22	0.32	(0.12–0.83)	9/22	0.39	(0.13–1.15)	5/22	0.31	(0.07–1.28)
3nd tertile	9.0	9/16	0.31	(0.11–0.90)	5/16	0.32	(0.09–1.13)	4/16	0.45	(0.10–2.00)
Linear trend			0.66	(0.47–0.91)		0.67	(0.46–0.98)		0.66	(0.41–1.08)

## Supplementary Materials

The following are available online at https://www.mdpi.com/2072-6643/12/12/3682/s1. Supplemental Table S1: Clinical diagnosis of early-onset dementia cases; Supplemental Table S2: Food and beverages mean (standard deviation (SD)) intake and number (%) of consumers in the study population. Early-onset dementia (EOD); Supplemental Table S3: Food and beverages mean (standard deviation (SD)) intake and number (%) of consumers according to the EOD subtype. Early-onset Alzheimer’s dementia (EO-AD) and early-onset frontotemporal dementia spectrum (EO-FTD); Supplemental Table S4: Odds ratio (OR) and 95% confidence intervals (CI) for early-onset dementia (EOD), early-onset Alzheimer’s dementia (EO-AD), and early-onset frontotemporal dementia spectrum (EO-FTD) for increasing tertiles of food and beverage intake; Supplemental Figures S1–S10: Spline regression analysis of risk of early-onset Alzheimer’s dementia (EO-AD) for an increasing intake of food: Cereals and cereal products (1); meats and meat products (2); milk, dairy products, and eggs (3); fish and seafood (4); vegetables (5); mushrooms, legumes, and potatoes (6); fresh and dry fruits (7); sweets, chocolate, cakes, etc. (8); oils and fats (9); beverages (10). The black line indicates the odds ratio for dementia risk; the dash gray lines are 95% confidence limits; the reference line at 1.0 with black spikes indicates the distribution of intake of participants. Note: Spline analysis was not possible for offal and most beverages due to a few subjects reporting consumption which is different from the null value (tea, red, white, aperitif wines and beers, spirits and soft drinks); Supplemental Figures S11–S20: Spline regression analysis of risk of early-onset frontotemporal dementia spectrum (EO-FTD) for an increasing intake of food: Cereals and cereal products (1); meats and meat products (2); milk, dairy products, and eggs (3); fish and seafood (4); vegetables (5); mushrooms, legumes, and potatoes (6); fresh and dry fruits (7); sweets, chocolate, cakes, etc. (8); oils and fats (9); beverages (10). The black line indicates the odds ratio for dementia risk; the dash gray lines are 95% confidence limits; the reference line at 1.0 with black spikes indicates the distribution of intake of participants. Note: Spline analysis was not possible for offal and most beverages due to a few subjects reporting consumption which is different from the null value (tea, red, white, aperitif wines and beers, spirits, fruit juices, and soft drinks); Supplemental Figure S21: Spline regression analysis of risk of early-onset Alzheimer’s dementia (EO-AD) for increasing adherence to the Greek-Mediterranean (GM) diet, Dietary Approaches to Stop Hypertension (DASH) diet, and Mediterranean-DASH Intervention for Neurodegenerative Delay (MIND) diet. The black line indicates the odds ratio for dementia risk; the dash gray lines are 95% confidence limits; the reference line at 1.0 with gray bars shows the distribution of dietary pattern scores; Supplemental Figure S22: Spline regression analysis of risk of frontotemporal dementia spectrum (EO-FTD) for increasing adherence to dietary patterns. Greek-Mediterranean (GM) diet, Dietary Approaches to Stop Hypertension (DASH) diet, and Mediterranean-DASH Intervention for Neurodegenerative Delay (MIND) diet. The black line indicates the odds ratio for dementia risk; the dash gray lines are 95% confidence limits; the reference line at 1.0 with gray bars shows the distribution of dietary pattern scores.

## References

[1] WHO (2019). Dementia. https://www.who.int/news-room/fact-sheets/detail/dementia.

[2] Wolters F.J., Ikram M.A. (2018). Epidemiology of dementia: The burden on society, the challenges for research. Methods Mol. Biol..

[3] Rossor M.N., Fox N.C., Mummery C.J., Schott J.M., Warren J.D. (2010). The diagnosis of young-onset dementia. Lancet Neurol..

[4] Vieira R.T., Caixeta L., Machado S., Silva A.C., Nardi A.E., Arias-Carrion O., Carta M.G. (2013). Epidemiology of early-onset dementia: A review of the literature. Clin. Pract. Epidemiol. Ment. Health.

[5] Sakata N., Okumura Y. (2017). Job loss after diagnosis of early-onset dementia: A matched cohort study. J. Alzheimers Dis..

[6] Sikes P., Hall M. (2018). The impact of parental young onset dementia on children and young people’s educational careers. Br. Educ. Res. J..

[7] Chiari A., Vinceti G., Adani G., Tondelli M., Galli C., Fiondella L., Costa M., Molinari M.A., Filippini T., Zamboni G. (2020). Epidemiology of early onset dementia and its clinical presentations in the province of Modena, Italy. Alzheimer’s Dement..

[8] Jarmolowicz A.I., Chen H.Y., Panegyres P.K. (2015). The patterns of inheritance in early-onset dementia: Alzheimer’s disease and frontotemporal dementia. Am. J. Alzheimers Dis. Other Demen..

[9] Koedam E.L., Lauffer V., van der Vlies A.E., van der Flier W.M., Scheltens P., Pijnenburg Y.A. (2010). Early-versus late-onset Alzheimer’s disease: More than age alone. J. Alzheimers Dis..

[10] Cations M., Withall A., Low L.F., Draper B. (2016). What is the role of modifiable environmental and lifestyle risk factors in young onset dementia?. Eur. J. Epidemiol..

[11] Cations M., Withall A., Draper B. (2019). Modifiable risk factors for young onset dementia. Curr. Opin. Psychiatry.

[12] Angeloni C., Businaro R., Vauzour D. (2020). The role of diet in preventing and reducing cognitive decline. Curr. Opin. Psychiatry.

[13] Livingston G., Huntley J., Sommerlad A., Ames D., Ballard C., Banerjee S., Brayne C., Burns A., Cohen-Mansfield J., Cooper C. (2020). Dementia prevention, intervention, and care: 2020 report of the Lancet Commission. Lancet.

[14] Stamati P., Siokas V., Aloizou A.M., Karampinis E., Arseniou S., Rakitskii V.N., Tsatsakis A., Spandidos D.A., Gozes I., Mitsias P.D. (2019). Does SCFD1 rs10139154 polymorphism decrease Alzheimer’s disease risk?. J. Mol. Neurosci..

[15] Morris M.C., Evans D.A., Tangney C.C., Bienias J.L., Wilson R.S. (2005). Fish consumption and cognitive decline with age in a large community study. Arch. Neurol..

[16] Morris M.C., Evans D.A., Tangney C.C., Bienias J.L., Wilson R.S. (2006). Associations of vegetable and fruit consumption with age-related cognitive change. Neurology.

[17] Nooyens A.C., Bueno-de-Mesquita H.B., van Boxtel M.P., van Gelder B.M., Verhagen H., Verschuren W.M. (2011). Fruit and vegetable intake and cognitive decline in middle-aged men and women: The Doetinchem Cohort Study. Br. J. Nutr..

[18] Martinez-Lapiscina E.H., Clavero P., Toledo E., Estruch R., Salas-Salvado J., San Julian B., Sanchez-Tainta A., Ros E., Valls-Pedret C., Martinez-Gonzalez M.A. (2013). Mediterranean diet improves cognition: The PREDIMED-NAVARRA randomised trial. J. Neurol. Neurosurg. Psychiatry.

[19] Morris M.C., Tangney C.C., Wang Y., Sacks F.M., Barnes L.L., Bennett D.A., Aggarwal N.T. (2015). MIND diet slows cognitive decline with aging. Alzheimers Dement..

[20] Smith P.J., Blumenthal J.A., Babyak M.A., Craighead L., Welsh-Bohmer K.A., Browndyke J.N., Strauman T.A., Sherwood A. (2010). Effects of the dietary approaches to stop hypertension diet, exercise, and caloric restriction on neurocognition in overweight adults with high blood pressure. Hypertension.

[21] Tanaka T., Talegawkar S.A., Jin Y., Colpo M., Ferrucci L., Bandinelli S. (2018). Adherence to a mediterranean diet protects from cognitive decline in the invecchiare in Chianti study of aging. Nutrients.

[22] Berti V., Murray J., Davies M., Spector N., Tsui W.H., Li Y., Williams S., Pirraglia E., Vallabhajosula S., McHugh P. (2015). Nutrient patterns and brain biomarkers of Alzheimer’s disease in cognitively normal individuals. J. Nutr. Health Aging.

[23] Mosconi L., Murray J., Davies M., Williams S., Pirraglia E., Spector N., Tsui W.H., Li Y., Butler T., Osorio R.S. (2014). Nutrient intake and brain biomarkers of Alzheimer’s disease in at-risk cognitively normal individuals: A cross-sectional neuroimaging pilot study. BMJ Open.

[24] Adani G., Filippini T., Garuti C., Malavolti M., Vinceti G., Zamboni G., Tondelli M., Galli C., Costa M., Vinceti M. (2020). Environmental risk factors for early-onset Alzheimer’s dementia and frontotemporal dementia: A case-control study in northern Italy. Int. J. Environ. Res. Public Health.

[25] Filippini T., Fiore M., Tesauro M., Malagoli C., Consonni M., Violi F., Arcolin E., Iacuzio L., Oliveri Conti G., Cristaldi A. (2020). Clinical and lifestyle factors and risk of amyotrophic lateral sclerosis: A population-based case-control study. Int. J. Environ. Res. Public Health.

[26] Pala V., Sieri S., Palli D., Salvini S., Berrino F., Bellegotti M., Frasca G., Tumino R., Sacerdote C., Fiorini L. (2003). Diet in the Italian EPIC cohorts: Presentation of data and methodological issues. Tumori.

[27] Pasanisi P., Berrino F., Bellati C., Sieri S., Krogh V. (2002). Validity of the Italian EPIC questionnaire to assess past diet. IARC Sci. Publ..

[28] Filippini T., Malagoli C., Wise L.A., Malavolti M., Pellacani G., Vinceti M. (2019). Dietary cadmium intake and risk of cutaneous melanoma: An Italian population-based case-control study. J. Trace Elem. Med. Biol..

[29] Malavolti M., Fairweather-Tait S.J., Malagoli C., Vescovi L., Vinceti M., Filippini T. (2020). Lead exposure in an Italian population: Food content, dietary intake and risk assessment. Food Res. Int..

[30] Sieri S., Krogh V., Saieva C., Grobbee D.E., Bergmann M., Rohrmann S., Tjonneland A., Ferrari P., Chloptsios Y., Dilis V. (2009). Alcohol consumption patterns, diet and body weight in 10 European countries. Eur. J. Clin. Nutr..

[31] Trichopoulou A., Costacou T., Bamia C., Trichopoulos D. (2003). Adherence to a Mediterranean diet and survival in a Greek population. N. Engl. J. Med..

[32] Appel L.J., Moore T.J., Obarzanek E., Vollmer W.M., Svetkey L.P., Sacks F.M., Bray G.A., Vogt T.M., Cutler J.A., Windhauser M.M. (1997). A clinical trial of the effects of dietary patterns on blood pressure. DASH Collaborative Research Group. N. Engl. J. Med..

[33] Sacks F.M., Svetkey L.P., Vollmer W.M., Appel L.J., Bray G.A., Harsha D., Obarzanek E., Conlin P.R., Miller E.R., Simons-Morton D.G. (2001). Effects on blood pressure of reduced dietary sodium and the Dietary Approaches to Stop Hypertension (DASH) diet. DASH-Sodium Collaborative Research Group. N. Engl. J. Med..

[34] Malagoli C., Malavolti M., Agnoli C., Crespi C.M., Fiorentini C., Farnetani F., Longo C., Ricci C., Albertini G., Lanzoni A. (2015). Diet quality and risk of melanoma in an Italian population. J. Nutr..

[35] Morris M.C., Tangney C.C., Wang Y., Sacks F.M., Bennett D.A., Aggarwal N.T. (2015). MIND diet associated with reduced incidence of Alzheimer’s disease. Alzheimers Dement..

[36] Agnoli C., Krogh V., Grioni S., Sieri S., Palli D., Masala G., Sacerdote C., Vineis P., Tumino R., Frasca G. (2011). A priori-defined dietary patterns are associated with reduced risk of stroke in a large Italian cohort. J. Nutr..

[37] McGrattan A.M., McGuinness B., McKinley M.C., Kee F., Passmore P., Woodside J.V., McEvoy C.T. (2019). Diet and inflammation in cognitive ageing and Alzheimer’s disease. Curr. Nutr. Rep..

[38] Fung T.T., Chiuve S.E., McCullough M.L., Rexrode K.M., Logroscino G., Hu F.B. (2008). Adherence to a DASH-style diet and risk of coronary heart disease and stroke in women. Arch. Intern. Med..

[39] Di Marco L.Y., Marzo A., Munoz-Ruiz M., Ikram M.A., Kivipelto M., Ruefenacht D., Venneri A., Soininen H., Wanke I., Ventikos Y.A. (2014). Modifiable lifestyle factors in dementia: A systematic review of longitudinal observational cohort studies. J. Alzheimers Dis..

[40] Devore E.E., Kang J.H., Breteler M.M., Grodstein F. (2012). Dietary intakes of berries and flavonoids in relation to cognitive decline. Ann. Neurol..

[41] Roman G.C., Jackson R.E., Gadhia R., Roman A.N., Reis J. (2019). Mediterranean diet: The role of long-chain omega-3 fatty acids in fish; polyphenols in fruits, vegetables, cereals, coffee, tea, cacao and wine; probiotics and vitamins in prevention of stroke, age-related cognitive decline, and Alzheimer disease. Rev. Neurol..

[42] Zielinska M.A., Bialecka A., Pietruszka B., Hamulka J. (2017). Vegetables and fruit, as a source of bioactive substances, and impact on memory and cognitive function of elderly. Postepy Hig. Med. Dosw. (Online).

[43] Barberger-Gateau P., Raffaitin C., Letenneur L., Berr C., Tzourio C., Dartigues J.F., Alperovitch A. (2007). Dietary patterns and risk of dementia: The Three-City cohort study. Neurology.

[44] Kang J.H., Ascherio A., Grodstein F. (2005). Fruit and vegetable consumption and cognitive decline in aging women. Ann. Neurol..

[45] Morris M.C., Wang Y., Barnes L.L., Bennett D.A., Dawson-Hughes B., Booth S.L. (2018). Nutrients and bioactives in green leafy vegetables and cognitive decline: Prospective study. Neurology.

[46] Agarwal P., Holland T.M., Wang Y., Bennett D.A., Morris M.C. (2019). Association of strawberries and anthocyanidin intake with Alzheimer’s dementia risk. Nutrients.

[47] Devore E.E., Grodstein F., van Rooij F.J., Hofman A., Rosner B., Stampfer M.J., Witteman J.C., Breteler M.M. (2009). Dietary intake of fish and omega-3 fatty acids in relation to long-term dementia risk. Am. J. Clin. Nutr..

[48] Larrieu S., Letenneur L., Helmer C., Dartigues J.F., Barberger-Gateau P. (2004). Nutritional factors and risk of incident dementia in the PAQUID longitudinal cohort. J. Nutr. Health Aging.

[49] Morris M.C., Evans D.A., Bienias J.L., Tangney C.C., Bennett D.A., Wilson R.S., Aggarwal N., Schneider J. (2003). Consumption of fish and n-3 fatty acids and risk of incident Alzheimer disease. Arch. Neurol..

[50] Samieri C., Morris M.C., Bennett D.A., Berr C., Amouyel P., Dartigues J.F., Tzourio C., Chasman D.I., Grodstein F. (2018). Fish intake, genetic predisposition to Alzheimer disease, and decline in global cognition and memory in 5 cohorts of older persons. Am. J. Epidemiol..

[51] Pappalardo A.M., Copat C., Ferrito V., Grasso A., Ferrante M. (2017). Heavy metal content and molecular species identification in canned tuna: Insights into human food safety. Mol. Med. Rep..

[52] Filippini T., Cilloni S., Malavolti M., Violi F., Malagoli C., Tesauro M., Bottecchi I., Ferrari A., Vescovi L., Vinceti M. (2018). Dietary intake of cadmium, chromium, copper, manganese, selenium and zinc in a Northern Italy community. J. Trace Elem. Med. Biol..

[53] Russo R., Lo Voi A., De Simone A., Serpe F.P., Anastasio A., Pepe T., Cacace D., Severino L. (2013). Heavy metals in canned tuna from Italian markets. J. Food Prot..

[54] Storelli M.M., Barone G., Cuttone G., Giungato D., Garofalo R. (2010). Occurrence of toxic metals (Hg, Cd and Pb) in fresh and canned tuna: Public health implications. Food Chem. Toxicol..

[55] Vinceti M., Mandrioli J., Borella P., Michalke B., Tsatsakis A., Finkelstein Y. (2014). Selenium neurotoxicity in humans: Bridging laboratory and epidemiologic studies. Toxicol. Lett..

[56] Vinceti M., Filippini T., Mandrioli J., Violi F., Bargellini A., Weuve J., Fini N., Grill P., Michalke B. (2017). Lead, cadmium and mercury in cerebrospinal fluid and risk of amyotrophic lateral sclerosis: A case-control study. J. Trace Elem. Med. Biol..

[57] Kesse-Guyot E., Assmann K.E., Andreeva V.A., Ferry M., Hercberg S., Galan P. (2016). Consumption of dairy products and cognitive functioning: Findings from the SU.VI.MAX 2 study. J. Nutr. Health Aging.

[58] Otsuka R., Kato Y., Nishita Y., Tange C., Nakamoto M., Tomida M., Imai T., Ando F., Shimokata H. (2014). Cereal intake increases and dairy products decrease risk of cognitive decline among elderly female Japanese. J. Prev. Alzheimers Dis..

[59] Lee J., Fu Z., Chung M., Jang D.J., Lee H.J. (2018). Role of milk and dairy intake in cognitive function in older adults: A systematic review and meta-analysis. Nutr. J..

[60] Ozawa M., Ninomiya T., Ohara T., Doi Y., Uchida K., Shirota T., Yonemoto K., Kitazono T., Kiyohara Y. (2013). Dietary patterns and risk of dementia in an elderly Japanese population: The hisayama Study1–3. Am. J. Clin. Nutr..

[61] Rahman A., Baker P.S., Allman R.M., Zamrini E. (2007). Dietary factors and cognitive impairment in community-dwelling elderly. J. Nutr. Health Aging.

[62] Ano Y., Nakayama H. (2018). Preventive effects of dairy products on dementia and the underlying mechanisms. Int. J. Mol. Sci..

[63] Willett W.C., Sacks F., Trichopoulou A., Drescher G., Ferro-Luzzi A., Helsing E., Trichopoulos D. (1995). Mediterranean diet pyramid: A cultural model for healthy eating. Am. J. Clin. Nutr..

[64] Wengreen H., Munger R.G., Cutler A., Quach A., Bowles A., Corcoran C., Tschanz J.T., Norton M.C., Welsh-Bohmer K.A. (2013). Prospective study of Dietary Approaches to Stop Hypertension- and Mediterranean-style dietary patterns and age-related cognitive change: The cache county study on memory, health and aging. Am. J. Clin. Nutr..

[65] Slavin J. (2004). Whole grains and human health. Nutr. Res. Rev..

[66] Akbaraly T.N., Singh-Manoux A., Marmot M.G., Brunner E.J. (2009). Education attenuates the association between dietary patterns and cognition. Dement. Geriatr. Cogn. Disord..

[67] Pilleron S., Desport J.C., Jesus P., Mbelesso P., Ndamba-Bandzouzi B., Dartigues J.F., Clement J.P., Preux P.M., Guerchet M. (2015). Diet, Alcohol consumption and cognitive disorders in central Africa: A study from the EPIDEMCA program. J. Nutr. Health Aging.

[68] Morris M.C., Tangney C.C. (2014). Dietary fat composition and dementia risk. Neurobiol. Aging.

[69] Crichton G.E., Elias M.F., Alkerwi A. (2016). Chocolate intake is associated with better cognitive function: The maine-syracuse longitudinal study. Appetite.

[70] Moreira A., Diogenes M.J., de Mendonca A., Lunet N., Barros H. (2016). Chocolate consumption is associated with a lower risk of cognitive decline. J. Alzheimers Dis..

[71] Barrera-Reyes P.K., de Lara J.C., Gonzalez-Soto M., Tejero M.E. (2020). Effects of cocoa-derived polyphenols on cognitive function in humans. Systematic review and analysis of methodological aspects. Plant. Foods Hum. Nutr..

[72] Calabro R.S., De Cola M.C., Gervasi G., Portaro S., Naro A., Accorinti M., Manuli A., Marra A., De Luca R., Bramanti P. (2019). The efficacy of cocoa polyphenols in the treatment of mild cognitive impairment: A retrospective study. Medicina.

[73] Larsson S.C., Orsini N. (2018). Coffee consumption and risk of dementia and Alzheimer’s disease: A dose-response meta-analysis of prospective studies. Nutrients.

[74] Filippini T., Tancredi S., Malagoli C., Cilloni S., Malavolti M., Violi F., Vescovi L., Bargellini A., Vinceti M. (2019). Aluminum and tin: Food contamination and dietary intake in an Italian population. J. Trace Elem. Med. Biol..

[75] Malagoli C., Malavolti M., Farnetani F., Longo C., Filippini T., Pellacani G., Vinceti M. (2019). Food and beverage consumption and melanoma risk: A population-based case-control study in northern Italy. Nutrients.

[76] Lao Y., Hou L., Li J., Hui X., Yan P., Yang K. (2020). Association between alcohol intake, mild cognitive impairment and progression to dementia: A dose-response meta-analysis. Aging Clin. Exp. Res..

[77] Reale M., Costantini E., Jagarlapoodi S., Khan H., Belwal T., Cichelli A. (2020). Relationship of wine consumption with Alzheimer’s disease. Nutrients.

[78] Deng J., Zhou D.H., Li J., Wang Y.J., Gao C., Chen M. (2006). A 2-year follow-up study of alcohol consumption and risk of dementia. Clin. Neurol. Neurosurg..

[79] Orgogozo J.M., Dartigues J.F., Lafont S., Letenneur L., Commenges D., Salamon R., Renaud S., Breteler M.B. (1997). Wine consumption and dementia in the elderly: A prospective community study in the Bordeaux area. Rev. Neurol..

[80] Xu W., Wang H., Wan Y., Tan C., Li J., Tan L., Yu J.T. (2017). Alcohol consumption and dementia risk: A dose-response meta-analysis of prospective studies. Eur. J. Epidemiol..

[81] Berendsen A.M., Kang J.H., Feskens E.J.M., de Groot C., Grodstein F., van de Rest O. (2018). Association of long-term adherence to the MIND diet with cognitive function and cognitive decline in American women. J. Nutr. Health Aging.

[82] Feart C., Samieri C., Rondeau V., Amieva H., Portet F., Dartigues J.F., Scarmeas N., Barberger-Gateau P. (2009). Adherence to a Mediterranean diet, cognitive decline, and risk of dementia. JAMA.

[83] Munoz-Garcia M.I., Toledo E., Razquin C., Dominguez L.J., Maragarone D., Martinez-Gonzalez J., Martinez-Gonzalez M.A. (2020). “A priori” dietary patterns and cognitive function in the SUN project. Neuroepidemiology.

[84] Scarmeas N., Stern Y., Tang M.X., Mayeux R., Luchsinger J.A. (2006). Mediterranean diet and risk for Alzheimer’s disease. Ann. Neurol..

[85] Tangney C., Hong L., Barnes L.L., Schneider J., Bennett D., Morris M. (2013). O1–05–03: Accordance to Dietary Approaches to Stop Hypertension (DASH) is associated with slower cognitive decline. Alzheimers Dement..

[86] Tangney C.C., Li H., Wang Y., Barnes L., Schneider J.A., Bennett D.A., Morris M.C. (2014). Relation of DASH- and Mediterranean-like dietary patterns to cognitive decline in older persons. Neurology.

[87] Hosking D.E., Eramudugolla R., Cherbuin N., Anstey K.J. (2019). MIND not Mediterranean diet related to 12-year incidence of cognitive impairment in an Australian longitudinal cohort study. Alzheimers Dement..

[88] Morris M.C., Tangney C.C., Wang Y., Barnes L.L., Bennett D., Aggarwal N. (2014). MIND diet score more predictive than DASH or Mediterranean Diet scores. Alzheimer’s Dement. J. Alzheimer’s Assoc..

[89] Agarwal P., Wang Y., Buchman A.S., Holland T.M., Bennett D.A., Morris M.C. (2018). MIND diet associated with reduced incidence and delayed progression of parkinsonism in old age. J. Nutr Health Aging.

[90] Cherian L., Wang Y., Fakuda K., Leurgans S., Aggarwal N., Morris M. (2019). Mediterranean-Dash Intervention for Neurodegenerative Delay (MIND) diet slows cognitive decline after stroke. J. Prev. Alzheimers Dis..

[91] Cherian L., Wang Y., Holland T., Agarwal P., Aggarwal N., Morris M.C. (2020). DASH and Mediterranean-Dash Intervention for Neurodegenerative Delay (MIND) diets are associated with fewer depressive symptoms over time. J. Gerontol. A Biol. Sci. Med. Sci..

[92] Morris M.C. (2018). MIND Diet Intervention and Cognitive Decline (MIND). https://mind-diet-trial.org/meet-the-researchers/.

[93] Morris M.C. (2016). Nutrition and risk of dementia: Overview and methodological issues. Ann. N. Y. Acad. Sci..

[94] Biazus-Sehn L.F., Schuch F.B., Firth J., Stigger F.S. (2020). Effects of physical exercise on cognitive function of older adults with mild cognitive impairment: A systematic review and meta-analysis. Arch. Gerontol. Geriatr..

[95] Whitty E., Mansour H., Aguirre E., Palomo M., Charlesworth G., Ramjee S., Poppe M., Brodaty H., Kales H.C., Morgan-Trimmer S. (2020). Efficacy of lifestyle and psychosocial interventions in reducing cognitive decline in older people: Systematic review. Ageing Res. Rev..

[96] Benedetti I., Biggeri L., Laureti T., Secondi L. (2016). Exploring the Italians’ food habits and tendency towards a sustainable diet: The Mediterranean eating pattern. Agric. Agric. Sci. Procedia.

