# Supplementary material for: Dietary Habits and Risk of Early-Onset Dementia in an Italian Case-Control Study
Source: Nutrients. 2020 Nov 29;12(12):3682. doi: 10.3390/nu12123682 (PMC7760835; doi:10.3390/nu12123682)

nutrients

MDPI

SUPPLEMENTAL MATERIAL

Supplemental Table S1. Clinical diagnosis of early onset dementia cases.

Dementia diagnosis	N (%)
Alzheimer's dementia	30 (55.6)
Frontotemporal dementia spectrum	18 (33.3)
Frontotemporal dementia	16 (29.6)
Progressive supranuclear palsy	2 (3.7)
Vascular dementia	4 (7.4)
Cerebral amyloid angiopathy	1 (1.9)
Lewy body dementia	1 (1.9)

Supplemental Table S2. Food and beverages mean (standard deviation (SD)) intake and number (%) of consumers in the study population. Early onset dementia (EOD).

	Controls		EOD Cases	
	Mean (SD)	N (%)	Mean (SD)	N (%)
**Total energy intake (Kcal/day)**	1867.8 (803.9)	-	2010.4 (707.0)	-
**Food (g/day)**		54 (100)		54 (100)
**Cereals and cereal products**	168.9 (82.7)	0 (0.0)	185.1 (116.5)	1 (1.85)
Pasta and other grains	42.0 (35.0)	1 (1.85)	56.4 (37.3)	1 (1.85)
Rice	4.2 (4.2)	11 (20.37)	6.7 (8.5)	11 (20.37)
Bread	72.4 (59.4)	2 (3.70)	83.2 (81.1)	8 (14.81)
Pizza, crackers, and other salty snacks	50.3 (38.1)	1 (1.85)	38.8 (32.4)	2 (3.70)
**Meats and meat products**	96.9 (54.6)	1 (1.85)	106.1 (59.8)	0 (0.0)
Red meat	51.3 (40.0)	2 (3.70)	54.2 (45.0)	2 (3.70)
White meat	21.8 (18.8)	3 (5.56)	27.8 (23.9)	1 (1.85)
Processed meat	22.2 (18.2)	1 (1.85)	23.1 (19.4)	0 (0.0)
Offal	1.7 (5.9)	40 (74.07)	1.1 (3.2)	41 (75.93)
**Milk and dairy products**	182.5 (139.8)	0 (0.0)	276.1 (300.4)	0 (0.0)
Milk and yogurt	152.2 (133.5)	12 (22.22)	238.1 (299.2)	10 (18.52)
Milk	108.9 (112.8)	17 (31.48)	179.3 (291.6)	17 (31.48)
Yogurt	43.3 (72.5)	27 (50.00)	58.8 (92.0)	24 (44.44)
Cheese	30.3 (23.6)	(0.0)	38.0 (32.8)	0 (0.0)
Fresh cheese	12.4 (13.5)	8 (14.81)	13.7 (14.2)	12 (22.22)
Aged cheese	17.1 (14.9)	3 (5.56)	22.4 (23.3)	2 (3.70)
**Eggs**	13.2 (13.1)	4 (7.41)	14.8 (11.6)	3 (5.56)
**Fish and seafood**	30.9 (23.6)	4 (7.41)	30.0 (24.1)	4 (7.41)
Fish	24.8 (19.0)	5 (9.26)	24.9 (21.2)	5 (9.26)
Preserved and tinned fish	8.7 (7.7)	8 (14.81)	12.1 (14.6)	7 (12.96)
Non-piscivorous fish	8.3 (13.5)	14 (25.93)	7.9 (10.9)	14 (25.93)
Piscivorous fish	7.9 (11.0)	17 (31.48)	4.9 (7.2)	21 (38.89)
Crustaceans and molluscs	6.0 (9.2)	8 (22.22)	5.1 (7.0)	14 (25.93)
**All vegetables**	143.0 (91.8)	0 (0.0)	113.5 (78.5)	0 (0.0)
Leafy vegetables	26.7 (22.1)	0 (0.0)	20.2 (16.6)	0 (0.0)
Tomatoes	47.2 (52.6)	0 (0.0)	41.3 (42.7)	1 (1.85)
Root vegetables	34.5 (34.1)	7 (12.96)	24.3 (27.1)	13 (24.07)
Cabbage	4.9 (7.7)	20 (37.04)	5.1 (8.4)	16 (29.63)

Other vegetables	29.6 (21.3)	0 (0.0)	22.6 (20.1)	3 (5.56)
**Mushrooms**	2.5 (2.6)	11 (20.37)	2.2 (2.8)	21 (38.89)
**Legumes**	21.1 (21.5)	0 (0.0)	19.6 (17.3)	4 (7.41)
**Potatoes**	22.5 (29.6)	0 (0.0)	15.7 (14.7)	3 (5.56)
**Fresh fruit**	248.3 (137.9)	0 (0.0)	259.0 (155.4)	0 (0.0)
Citrus fruit	58.5 (50.4)	2 (3.70)	45.4 (39.5)	5 (9.26)
All other fruit	189.8 (101.3)	0 (0.0)	213.7 (133.1)	2 (3.70)
**Dry fruits, nuts and seeds**	3.9 (5.1)	4 (7.41)	2.3 (4.1)	8 (14.81)
Dry fruits	0.6 (1.2)	20 (37.04)	0.4 (1.2)	29 (53.70)
Nuts and seeds	3.3 (4.7)	5 (9.26)	1.9 (3.4)	8 (14.81)
**Sweets, chocolate, cakes, etc.**	113.9 (130.4)	1 (1.85)	128.8 (84.3)	1 (1.85)
Sugar, non-chocolate confectionery	26.7 (39.4)	11 (20.37)	27.5 (39.1)	10 (18.52)
Chocolate, candy bars, etc.	7.7 (15.4)	22 (40.74)	4.1 (5.8)	19 (35.19)
Ice-cream	15.8 (22.8)	10 (18.52)	22.8 (27.4)	8 (14.81)
Cakes, pies and pastries	52.0 (101.3)	6 (11.11)	55.6 (49.7)	7 (12.96)
Biscuits, dry cakes	11.7 (15.1)	16 (29.63)	18.9 (23.2)	13 (24.07)
**Oils and fats**	25.0 (13.3)	0 (0.0)	21.4 (11.6)	0 (0.0)
Vegetable fats and non-olive oils	3.0 (4.2)	16 (29.63)	1.9 (2.4)	18 (33.33)
Olive oil	19.6 (12.8)	1 (1.85)	17.4 (10.7)	2 (3.70)
Butter and other animal fats	2.4 (4.5)	6 (11.11)	2.1 (3.1)	11 (20.37)
**Beverages**				
Coffee and tea	137.8 (163.6)	2 (3.70)	112.9 (152.4)	10 (18.52)
Coffee	69.9 (44.2)	7 (12.96)	67.0 (71.0)	15 (27.78)
Tea	67.9 (156.8)	28 (51.85)	45.9 (136.7)	38 (70.37)
Wine	86.7 (159.0)	24 (44.44)	84.5 (139.0)	29 (53.70)
Red wine	28.5 (64.7)	29 (53.70)	42.8 (89.4)	34 (62.96)
White wine	58.2 (130.9)	29 (53.70)	41.7 (101.4)	34 (62.96)
Aperitif wines and beers	44.9 (133.6)	25 (46.30)	17.1 (63.6)	37 (68.52)
Spirits and liqueurs	1.2 (4.2)	36 (66.67)	0.1 (0.4)	48 (88.89)
Fruit juices	68.8 (140.1)	30 (55.56)	113.3 (201.8)	19 (35.19)
Soft drinks	73.8 (184.8)	37 (68.52)	42.1 (94.5)	35 (64.81)
Alcohol intake	10.3 (15.7)	6 (11.11)	10.1 (19.5)	9 (18.67)

nutrients MDPI

Supplemental Table S3. Food and beverages mean (standard deviation (SD)) intake and number (%) of consumers according to the EOD subtype. Early-onset Alzheimer's dementia (EO-AD) and early-onset frontotemporal dementia spectrum (EO-FTD).

	EO-AD cases		EO-FTD	
	**Mean (SD)**	**N (%)**	**Mean (SD)**	**N (%)**
**Total energy intake (Kcal/day)**	1973.2 (769.1)		2106.0 (680.7)	
**Food (g/day)**		30 (100)		18 (100)
**Cereals and cereal products**	167.1 (111.5)	1 (3.33)	213.1 (136.9)	0 (0.0)
Pasta and other grains	50.2 (37.0)	1 (3.33)	62.3 (40.4)	0 (0.0)
Rice	7.0 (9.5)	5 (16.67)	5.3 (6.5)	5 (27.78)
Bread	71.9 (73.3)	6 (20.00)	105.9 (100.0)	1 (5.56)
Pizza, crackers, and other salty snacks	38.1 (27.7)	2 (6.67)	39.6 (41.5)	0 (0.0)
**Meats and meat products**	100.7 (58.8)	0 (0.0)	109.1 (59.3)	0 (0.0)
Red meat	51.0 (45.7)	0 (0.0)	51.4 (40.7)	1 (5.56)
White meat	27.3 (21.8)	0 (0.0)	31.4 (29.6)	0 (0.0)
Processed meat	21.4 (17.3)	0 (0.0)	25.4 (23.0)	0 (0.0)
Offal	1.1 (3.1)	22 (73.33)	1.0 (4.0)	17 (94.44)
**Milk and dairy products**	328.1 (366.5)	0 (0.0)	248.8 (183.6)	0 (0.0)
Milk and yogurt	290.9 (369.8)	4 (13.33)	208.8 (166.9)	4 (22.22)
Milk	213.6 (368.9)	9 (30.00)	172.1 (148.8)	5 (27.78)
Yogurt	77.3 (114.1)	13 (43.33)	36.6 (51.1)	9 (50.00)
Cheese	37.2 (40.2)	0 (0.0)	40.1 (23.3)	0 (0.0)
Fresh cheese	13.8 (16.7)	7 (23.33)	15.1 (9.8)	2 (11.11)
Aged cheese	21.9 (26.3)	1 (3.33)	23.0 (21.4)	0 (0.0)
**Eggs**	14.9 (13.4)	2 (6.67)	12.9 (8.4)	1 (5.56)
**Fish and seafood**	29.4 (24.2)	2 (6.67)	33.0 (25.8)	1 (5.56)
Fish	23.6 (19.7)	2 (6.67)	29.6 (24.6)	1 (5.56)
Preserved and tinned fish	10.9 (15.7)	3 (10.00)	15.7 (14.3)	2 (11.11)
Non-piscivorous fish	8.4 (9.4)	7 (23.33)	8.9 (14.3)	3 (16.67)
Piscivorous fish	4.4 (6.3)	11 (36.67)	5.1 (8.0)	7 (38.89)
Crustaceans and molluscs	5.8 (8.2)	6 (20.00)	3.4 (4.5)	7 (38.89)
**All vegetables**	117.3 (89.8)	0 (0.0)	111.4 (66.5)	0 (0.0)
Leafy vegetables	22.2 (18.2)	0 (0.0)	15.9 (11.9)	0 (0.0)
Tomatoes	40.5 (45.5)	0 (0.0)	41.6 (44.6)	1 (5.56)
Root vegetables	26.3 (30.5)	6 (20.00)	23.1 (23.2)	3 (16.67)
Cabbage	5.0 (8.5)	8 (26.67)	6.5 (9.4)	3 (16.67)
Other vegetables	23.3 (19.9)	2 (6.67)	24.2 (22.3)	0 (0.0)
**Mushrooms**	2.4 (3.1)	13 (43.33)	1.4 (2.1)	7 (38.89)
**Legumes**	19.3 (18.0)	3 (10.00)	20.2 (18.4)	1 (5.56)
**Potatoes**	16.5 (16.4)	2 (6.67)	13.4 (10.6)	1 (5.56)
**Fresh fruit**	245.2 (169.7)	0 (0.0)	261.9 (155.1)	0 (0.0)
Citrus fruit	38.0 (37.7)	3 (10.00)	51.3 (34.8)	2 (11.11)
All other fruit	207.2 (144.0)	2 (6.67)	210.5 (132.0)	0 (0.0)
**Dry fruits, nuts and seeds**	3.3 (5.1)	5 (16.67)	1.4 (2.1)	0 (0.0)
Dry fruits	0.6 (1.6)	18 (60.00)	0.3 (0.6)	7 (38.89)
Nuts and seeds	2.8 (4.1)	5 (16.67)	1.0 (2.0)	0 (0.0)
**Sweets, chocolate, cakes, etc.**	133.0 (97.7)	1 (3.33)	129.1 (58.3)	0 (0.0)
Sugar, non-chocolate confectionery	33.3 (47.3)	5 (16.67)	24.3 (26.9)	3 (16.67)
Chocolate, candy bars, etc.	3.9 (6.2)	12 (40.00)	3.9 (4.6)	5 (27.78)

Ice-cream	23.2 (33.0)	4 (13.33)	21.4 (15.4)	2 (11.11)
Cakes, pies and pastries	52.3 (53.2)	5 (16.67)	60.3 (41.3)	2 (11.11)
Biscuits, dry cakes	20.4 (21.1)	6 (20.00)	19.1 (29.2)	5 (27.78)
**Oils and fats**	21.8 (12.4)	1 (3.33)	21.3 (11.2)	0 (0.0)
Vegetable fats and non-olive oils	1.8 (2.5)	9 (30.00)	1.8 (2.1)	7 (38.89)
Olive oil	18.0 (11.4)	1 (3.33)	17.4 (10.3)	1 (5.56)
Butter and other animal fats	2.1 (2.8)	5 (16.67)	2.1 (3.9)	5 (27.78)
**Beverages**				
Coffee and tea	150.0 (191.7)	4 (13.33)	65.9 (55.8)	4 (22.22)
Coffee	77.8 (82.4)	8 (26.67)	62.8 (53.1)	4 (22.22)
Tea	72.1 (177.4)	19 (63.33)	3.1 (7.3)	15 (83.33)
Wine	72.7 (125.1)	18 (60.00)	111.3 (170.8)	8 (44.44)
Red wine	51.9 (109.9)	20 (66.67)	31.8 (56.4)	11 (61.11)
White wine	20.8 (40.3)	21 (70.00)	79.5 (161.6)	10 (55.56)
Aperitif wines and beers	16.0 (46.2)	21 (70.00)	24.7 (93.7)	10 (55.56)
Spirits and liqueurs	0.1 (0.5)	27 (90.00)	0.1 (0.2)	16 (88.89)
Fruit juices	100.9 (176.0)	9 (30.00)	117.6 (192.5)	8 (44.44)
Soft drinks	39.2 (87.8)	19 (63.33)	22.6 (53.1)	13 (72.22)
Alcohol intake	8.0 (12.7)	6 (20.00)	14.8 (29.1)	2 (11.11)

nutrients

MDPI

Supplemental Table S4. Odds ratio (OR) and 95% confidence intervals (CI) for early onset dementia (EOD), early onset Alzheimer's dementia (EO-AD) and early-onset frontotemporal dementia spectrum (EO-FTD) for increasing tertiles of food and beverage intake.

		All EOD			EO-AD			EO-FDT		
**Food items**	**Median**	**Cases/ controls**	**OR[a]**	**(95% CI)**	**Cases/ controls**	**OR[a]**	**(95% CI)**	**Cases/ controls**	**OR[a]**	**(95% CI)**
**Cereals and cereal products**										
1st tertile (ref.)	90.1	21/18	1.00	-	14/18	1.00	-	6/18	1.00	-
2nd tertile	154.0	7/18	0.30	(0.09 - 0.96)	3/18	0.18	(0.04 - 0.81)	3/18	0.38	(0.07 - 2.04)
3nd tertile	231.5	26/18	0.80	(0.27 - 2.38)	13/18	0.56	(0.15 - 2.02)	9/18	0.75	(0.16 - 3.40)
Linear trend (10g increase)			*1.01*	*(0.96 - 1.06)*		*0.98*	*(0.92 - 1.05)*		*1.04*	*(0.98 - 1.11)*
Pasta and other grains										
1st tertile (ref.)	12.8	12/17	1.00	-	9/17	1.00	-	3/17	1.00	-
2nd tertile	32.2	10/19	0.74	(0.24 - 2.25)	6/19	0.59	(0.17 - 2.11)	3/19	0.78	(0.13 - 4.76)
3nd tertile	80.1	32/18	2.57	(0.87 - 7.55)	15/18	1.58	(0.45 - 5.49)	12/18	3.48	(0.72 - 16.86)
Linear trend (10g increase)			*1.15*	*(1.00 - 1.32)*		*1.09*	*(0.92 - 1.29)*		*1.17*	*(0.97 - 1.40)*
Rice										
1st tertile (ref.)	0.5	23/25	1.00	-	12/25	1.00	-	10/25	1.00	-
2nd tertile	4.0	11/15	0.78	(0.28 - 2.15)	7/15	0.95	(0.29 - 3.11)	2/15	0.24	(0.04 - 1.42)
3nd tertile	8.4	20/14	1.45	(0.56 - 3.77)	11/14	1.68	(0.55 - 5.15)	6/14	0.81	(0.21 - 3.04)
Linear trend (10g increase)			*1.86*	*(0.94 - 3.97)*		*1.98*	*(0.94 - 4.20)*		*1.47*	*(0.49 - 4.37)*
Linear increase (1g increase)			*1.06*	*(0.99 - 1.14)*		*1.07*	*(0.99 - 1.15)*		*1.04*	*(0.93 - 1.16)*
Bread										
1st tertile (ref.)	17.2	21/18	1.00	-	15/18	1.00	-	5/18	1.00	-
2nd tertile	58.6	15/18	0.80	(0.30 - 2.16)	6/18	0.48	(0.14 - 1.60)	6/18	1.40	(0.33 - 5.84)
3nd tertile	111.7	18/18	0.63	(0.22 - 1.82)	9/18	0.47	(0.14 - 1.54)	7/18	1.02	(0.24 - 4.39)
Linear trend (10g increase)			*1.02*	*(0.96 - 1.08)*		*0.99*	*(0.92 - 1.07)*		*1.06*	*(0.98 - 1.15)*
Pizza, crackers, and other salty snacks										
1st tertile (ref.)	19.2	29/18	1.00	-	16/18	1.00	-	10/18	1.00	-
2nd tertile	45.8	11/18	0.30	(0.11 - 0.84)	4/18	0.18	(0.05 - 0.73)	5/18	0.42	(0.10 - 1.68)
3nd tertile	73.5	14/18	0.33	(0.12 - 0.93)	10/18	0.45	(0.14 - 1.43)	3/18	0.18	(0.03 - 0.90)

Linear trend (10g increase)			*0.86*	*(0.75 - 0.99)*		*0.83*	*(0.69 - 1.01)*		*0.88*	*(0.72 - 1.06)*
**Meats and meat products**										
1st tertile (ref.)	38.5	15/18	1.00	-	9/18	1.00	-	5/18	1.00	-
2nd tertile	90.2	19/18	1.37	(0.51 - 3.68)	11/18	1.18	(0.38 - 3.71)	6/18	1.16	(0.27 - 4.96)
3rd tertile	158.6	20/18	1.18	(0.41 - 3.45)	10/18	0.96	(0.27 - 3.39)	7/18	1.09	(0.24 - 4.86)
Linear trend (10g increase)			*1.02*	*(0.94 - 1.11)*		*1.00*	*(0.91 - 1.10)*		*1.02*	*(0.90 - 1.14)*
Red meat										
1st tertile (ref.)	12.4	19/19	1.00	-	11/19	1.00	-	7/19	1.00	-
2nd tertile	46.0	15/17	0.80	(0.29 - 2.23)	9/17	0.67	(0.20 - 2.29)	5/17	0.73	(0.17 - 3.05)
3rd tertile	92.9	20/18	1.04	(0.38 - 2.86)	10/18	0.81	(0.24 - 2.71)	6/18	0.75	(0.18 - 3.21)
Linear trend (10g increase)			*1.01*	*(0.91 - 1.12)*		*0.99*	*(0.88 - 1.12)*		*0.98*	*(0.84 - 1.15)*
White meat										
1st tertile (ref.)	4.9	15/20	1.00	-	9/20	1.00	-	5/20	1.00	-
2nd tertile	18.6	13/17	0.80	(0.28 - 2.29)	5/17	0.50	(0.13 - 1.89)	5/17	0.78	(0.17 - 3.50)
3rd tertile	38.4	26/17	1.85	(0.71 - 4.84)	16/17	2.08	(0.68 - 6.32)	8/17	1.47	(0.36 - 6.01)
Linear trend (10g increase)			*1.12*	*(0.92 - 1.36)*		*1.15*	*(0.90 - 1.46)*		*1.15*	*(0.89 - 1.48)*
Processed meat										
1st tertile (ref.)	6.2	20/18	1.00	-	11/18	1.00	-	7/18	1.00	-
2nd tertile	15.8	11/17	0.51	(0.18 - 1.47)	6/17	0.54	(0.16 - 1.87)	4/17	0.60	(0.14 - 2.64)
3rd tertile	45.0	23/19	0.94	(0.35 - 2.54)	13/19	1.01	(0.32 - 3.14)	7/19	0.79	(0.19 - 3.18)
Linear trend (10g increase)			*0.98*	*(0.78 - 1.24)*		*0.92*	*(0.69 - 1.23)*		*1.03*	*(0.75 - 1.40)*
Offal										
1st tertile (ref.)	0.0	42/42	1.00	-	23/42	1.00	-	17/42	1.00	-
2nd tertile	0.9	8/7	1.28	(0.40 - 4.07)	4/7	1.17	(0.30 - 4.67)	0/7	-	-
3rd tertile	8.5	4/5	0.57	(0.13 - 2.43)	3/5	0.90	(0.19 - 4.19)	1/5	0.24	(0.02 - 2.52)
Linear trend (10g increase)			*0.58*	*(0.24 - 1.42)*		*0.67*	*(0.24 - 1.88)*		*0.50*	*(0.13 - 1.95)*
Linear trend (1g increase)			*0.95*	*(0.87 - 1.04)*		*0.96*	*(0.87 - 1.07)*		*0.93*	*(0.81 - 1.07)*
**Milk and dairy products**										
1st tertile (ref.)	31.1	16/18	1.00	-	7/18	1.00	-	5/18	1.00	-
2nd tertile	149.1	14/18	1.05	(0.37 - 3.00)	8/18	1.40	(0.38 - 5.12)	4/18	0.92	(0.17 - 5.04)
3rd tertile	338.6	24/18	1.25	(0.48 - 3.25)	15/18	1.92	(0.61 - 6.05)	9/18	1.45	(0.37 - 5.67)
Linear trend (10g increase)			*1.02*	*(1.00 - 1.04)*		*1.03*	*(1.00 - 1.05)*		*1.02*	*(0.98 - 1.06)*
Milk and yogurt										

	1st tertile (ref.)	17.9	23/25	1.00	-	10/25	1.00	-	8/25	1.00	-
	2nd tertile	168.3	12/15	0.85	(0.32 - 2.28)	10/15	1.67	(0.55 - 5.12)	1/15	0.17	(0.02 - 1.61)
	3nd tertile	326.4	19/14	1.25	(0.50 - 3.16)	10/14	1.62	(0.53 - 4.98)	9/14	1.57	(0.46 - 5.38)
	Linear trend (10g increase)			*1.02*	*(1.00 - 1.04)*		*1.03*	*(1.00 - 1.05)*		*1.02*	*(0.98 - 1.06)*
Milk											
	1st tertile (ref.)	0.0	26/28	1.00	-	13/28	1.00	-	8/28	1.00	-
	2nd tertile	160.0	11/14	0.73	(0.26 - 1.06)	8/14	1.27	(0.39 - 4.12)	2/14	0.35	(0.06 - 2.15)
	3nd tertile	257.7	17/12	1.25	(0.48 - 3.26)	9/12	1.50	(0.49 - 4.59)	8/12	1.95	(0.53 - 7.16)
	Linear trend (10g increase)			*1.02*	*(0.99 - 1.04)*		*1.02*	*(0.99 - 1.05)*		*1.03*	*(0.99 - 1.08)*
Yogurt											
	1st tertile (ref.)	0.0	29/34	1.00	-	16/34	1.00	-	11/34	1.00	-
	2nd tertile	44.6	10/8	1.40	(0.46 - 4.21)	3/8	0.75	(0.17 - 3.37)	3/8	1.06	(0.20 - 5.67)
	3nd tertile	125.0	15/12	1.65	(0.64 - 4.28)	11/12	2.07	(0.72 - 5.98)	4/12	1.20	(0.30 - 4.80)
	Linear trend (10g increase)			*1.03*	*(0.98 - 1.08)*		*1.04*	*(0.99 - 1.10)*		*0.99*	*(0.91 - 1.08)*
Cheese											
	1st tertile (ref.)	10.3	13/18	1.00	-	10/18	1.00	-	2/18	1.00	-
	2nd tertile	22.9	16/18	1.03	(0.36 - 2.90)	9/18	0.78	(0.24 - 2.51)	5/18	2.51	(0.40 - 15.71)
	3nd tertile	49.6	25/18	1.88	(0.60 - 5.91)	11/18	0.97	(0.26 - 3.68)	11/18	7.05	(1.02 - 48.58)
	Linear trend (10g increase)			*1.09*	*(0.91 - 1.31)*		*1.06*	*(0.89 - 1.28)*		*1.15*	*(0.85 - 1.57)*
	Linear trend (1g increase)			*1.01*	*(0.99 - 1.03)*		*1.01*	*(0.99 - 1.02)*		*1.01*	*(0.98 - 1.05)*
Fresh cheese											
	1st tertile (ref.)	0.8	20/23	1.00	-	13/23	1.00	-	4/23	1.00	-
	2nd tertile	8.7	16/16	1.39	(0.53 - 3.65)	8/16	1.01	(0.33 - 3.09)	6/16	2.75	(0.60 - 12.62)
	3nd tertile	30.1	15/18	1.47	(0.53 - 4.12)	9/18	1.00	(0.30 - 3.41)	8/18	3.88	(0.77 - 19.58)
	Linear trend (10g increase)			*1.06*	*(0.77 - 1.47)*		*1.03*	*(0.73 - 1.47)*		*1.21*	*(0.72 - 2.03)*
	Linear trend (1g increase)			*1.01*	*(0.97 - 1.04)*		*1.00*	*(0.97 - 1.04)*		*1.02*	*(0.97 - 1.07)*
Aged cheese											
	1st tertile (ref.)	5.6	18/20	1.00	-	11/20	1.00	-	6/20	1.00	-
	2nd tertile	12.7	13/17	0.87	(0.32 - 2.38)	8/17	0.94	(0.30 - 3.00)	5/17	0.91	(0.21 - 3.95)

3rd tertile	28.6	23/17	1.37	(0.49 - 3.79)	11/17	1.15	(0.34 - 3.87)	7/17	0.91	(0.18 - 4.47)
Linear trend (10g increase)			*1.11*	*(0.85 - 1.45)*		*1.11*	*(0.83 - 1.47)*		*1.09*	*(0.72 - 1.64)*
Linear trend (1g increase)			*1.01*	*(0.98 - 1.04)*		*1.01*	*(0.98 - 1.04)*		*1.01*	*(0.97 - 1.05)*
**Eggs**										
1st tertile (ref.)	3.9	21/20	1.00	-	14/20	1.00	-	6/20	1.00	-
2nd tertile	10.4	13/17	0.64	(0.23 - 1.77)	6/17	0.44	(0.13 - 1.49)	5/17	1.06	(0.24 - 4.74)
3rd tertile	23.3	20/17	0.86	(0.31 - 2.34)	10/17	0.61	(0.19 - 1.94)	7/17	0.99	(0.24 - 4.09)
Linear trend (10g increase)			*1.02*	*(0.72 - 1.43)*		*1.02*	*(0.71 - 1.48)*		*0.84*	*(0.50 - 1.41)*
Linear trend (1g increase)			*1.00*	*(0.97 - 1.04)*		*1.00*	*(0.97 - 1.04)*		*0.98*	*(0.93 - 1.04)*
**Fish and seafood**										
1st tertile (ref.)	9.7	22/20	1.00	-	13/20	1.00	-	6/20	1.00	-
2nd tertile	28.4	18/17	0.98	(0.39 - 2.48)	9/17	0.83	(0.28 - 2.48)	7/17	1.40	(0.37 - 5.30)
3rd tertile	53.9	14/17	0.70	(0.26 - 1.86)	8/17	0.69	(0.22 - 2.12)	5/17	0.92	(0.21 - 3.95)
Linear trend (10g increase)			*0.97*	*(0.82 - 1.15)*		*0.97*	*(0.80 - 1.18)*		*1.04*	*(0.81 - 1.33)*
Fish										
1st tertile (ref.)	8.0	26/21	1.00	-	14/21	1.00	-	9/21	1.00	-
2nd tertile	25.3	13/17	0.58	(0.22 - 1.51)	9/17	0.79	(0.27 - 2.31)	2/17	0.24	(0.04 - 1.32)
3rd tertile	45.3	15/16	0.69	(0.26 - 1.85)	7/16	0.65	(0.20 - 2.10)	7/16	0.87	(0.23 - 3.25)
Linear trend (10g increase)			*0.98*	*(0.80 - 1.19)*		*0.96*	*(0.75 - 1.22)*		*1.09*	*(0.83 - 1.44)*
Preserved and tinned fish										
1st tertile (ref.)	2.0	19/22	1.00	-	12/22	1.00	-	5/22	1.00	-
2nd tertile	8.5	19/18	0.85	(0.32 - 2.29)	11/18	0.83	(0.26 - 2.70)	6/18	1.00	(0.23 - 4.39)
3rd tertile	17.6	16/14	1.22	(0.44 - 3.36)	7/14	0.88	(0.26 - 2.95)	7/14	1.88	(0.44 - 8.11)
Linear trend (10g increase)			*1.29*	*(0.88 - 1.88)*		*1.19*	*(0.79 - 1.80)*		*1.96*	*(1.04 - 3.70)*
Linear trend (1g increase)			*1.03*	*(0.99 - 1.07)*		*1.02*	*(0.98 - 1.06)*		*1.07*	*(1.00 - 1.14)*
Non-piscivorous fish										
1st tertile (ref.)	0.0	26/27	1.00	-	13/27	1.00	-	9/27	1.00	-
2nd tertile	5.7	14/14	0.86	(0.33 - 2.26)	7/14	0.91	(0.28 - 2.94)	5/14	0.77	(0.19 - 3.09)
3rd tertile	16.3	14/13	0.81	(0.29 - 2.27)	10/13	1.42	(0.45 - 4.42)	4/13	0.49	(0.10 - 2.40)
Linear trend (10g increase)			*0.91*	*(0.64 - 1.28)*		*0.97*	*(0.66 - 1.45)*		*0.95*	*(0.59 - 1.53)*
Linear trend (1g increase)			*0.99*	*(0.96 - 1.03)*		*1.00*	*(0.96 - 1.04)*		*1.00*	*(0.95 - 1.04)*
Piscivorous fish										
1st tertile (ref.)	0.0	34/29	1.00	-	20/29	1.00	-	11/29	1.00	-

2nd tertile	6.8	14/13	0.91	(0.35 - 2.32)	7/13	0.78	(0.25 - 2.39)	5/13	1.05	(0.28 - 3.86)
3nd tertile	24.4	6/12	0.48	(0.16 - 1.49)	3/12	0.36	(0.09 - 1.49)	2/12	0.58	(0.10 - 3.27)
Linear trend (10g increase)			*0.72*	*(0.46 - 1.14)*		*0.62*	*(0.34 - 1.14)*		*0.79*	*(0.41 - 1.54)*
Linear trend (1g increase)			*0.97*	*(0.92 - 1.01)*		*0.95*	*(0.90 - 1.01)*		*0.98*	*(0.91 - 1.04)*
Crustaceans and molluscs										
1st tertile (ref.)	0.3	28/26	1.00	-	14/26	1.00	-	11/26	1.00	-
2nd tertile	4.3	9/14	0.59	(0.21 - 1.65)	7/14	0.93	(0.29 - 2.96)	2/14	0.27	(0.05 - 1.55)
3nd tertile	10.6	17/14	1.22	(0.48 - 3.10)	9/14	1.16	(0.39 - 3.47)	5/14	1.31	(0.32 - 5.32)
Linear trend (10g increase)			*0.92*	*(0.55 - 1.53)*		*1.01*	*(0.58 - 1.74)*		*0.63*	*(0.20 - 1.98)*
Linear trend (1g increase)			*0.99*	*(0.94 - 1.04)*		*1.00*	*(0.95 - 1.06)*		*0.95*	*(0.85 - 1.07)*
**All vegetables**										
1st tertile (ref.)	72.1	29/18	1.00	-	17/18	1.00	-	9/18	1.00	-
2nd tertile	125.1	7/18	0.23	(0.08 - 0.70)	2/18	0.11	(0.02 - 0.59)	3/18	0.33	(0.07 - 1.53)
3nd tertile	189.3	18/18	0.53	(0.21 - 1.38)	11/18	0.62	(0.21 - 1.76)	6/18	0.60	(0.16 - 2.29)
Linear trend (10g increase)			*0.95*	*(0.90 - 1.00)*		*0.96*	*(0.90 - 1.01)*		*0.94*	*(0.86 - 1.02)*
Leafy vegetables										
1st tertile (ref.)	7.1	26/18	1.00	-	13/18	1.00	-	9/18	1.00	-
2nd tertile	18.5	16/18	0.56	(0.21 - 1.53)	9/18	0.62	(0.19 - 2.01)	7/18	0.64	(0.16 - 2.55)
3nd tertile	46.6	12/18	0.43	(0.15 - 1.19)	8/18	0.55	(0.17 - 1.80)	2/18	0.20	(0.03 - 1.19)
Linear trend (10g increase)			*0.82*	*(0.66 - 1.02)*		*0.87*	*(0.68 - 1.11)*		*0.66*	*(0.42 - 1.03)*
Linear trend (1g increase)			*0.98*	*(0.96 - 1.00)*		*0.99*	*(0.96 - 1.01)*		*0.96*	*(0.92 - 1.00)*
Tomatoes										
1st tertile (ref.)	14.2	21/18	1.00	-	14/18	1.00	-	6/18	1.00	-
2nd tertile	38.4	16/18	0.68	(0.26 - 1.78)	7/18	0.41	(0.12 - 1.33)	8/18	1.08	(0.27 - 4.26)
3nd tertile	68.1	17/18	0.74	(0.27 - 1.97)	9/18	0.60	(0.19 - 1.90)	4/18	0.51	(0.10 - 2.47)
Linear trend (10g increase)			*0.95*	*(0.87 - 1.04)*		*0.95*	*(0.86 - 1.06)*		*0.94*	*(0.82 - 1.08)*
Root vegetables										
1st tertile (ref.)	8.2	27/18	1.00	-	15/18	1.00	-	8/18	1.00	-
2nd tertile	21.3	14/18	0.57	(0.21 - 1.50)	6/18	0.33	(0.09 - 1.19)	7/18	1.12	(0.29 - 4.34)
3nd tertile	60.6	13/18	0.47	(0.18 - 1.27)	9/18	0.53	(0.17 - 1.65)	3/18	0.37	(0.07 - 1.95)
Linear trend (10g increase)			*0.89*	*(0.77 - 1.02)*		*0.91*	*(0.78 - 1.07)*		*0.85*	*(0.66 - 1.09)*
Linear trend (1g increase)			*0.99*	*(0.97 - 1.00)*		*0.99*	*(0.98 - 1.01)*		*0.98*	*(0.96 - 1.01)*
Cabbage										

nutrients

MDPI

1st tertile (ref.)	0.0	32/31	1.00	-	17/31	1.00	-	10/31	1.00	-
2nd tertile	4.5	12/12	0.90	(0.33 - 2.41)	7/12	0.89	(0.28 - 2.82)	5/12	1.26	(0.33 - 4.74)
3nd tertile	13.6	10/11	0.76	(0.26 - 2.18)	6/11	0.86	(0.25 - 2.95)	3/11	0.74	(0.15 - 3.60)
Linear trend (10g increase)			*0.95*	*(0.58 - 1.57)*		*0.93*	*(0.51 - 1.71)*		*1.22*	*(0.62 - 2.37)*
Linear trend (1g increase)			*1.00*	*(0.95 - 1.05)*		*0.99*	*(0.93 - 1.06)*		*1.02*	*(0.95 - 1.09)*
Other vegetables										
1st tertile (ref.)	9.2	25/18	1.00	-	11/18	1.00	-	9/18	1.00	-
2nd tertile	28.0	18/18	0.81	(0.31 - 2.11)	13/18	1.29	(0.43 - 3.88)	5/18	0.68	(0.17 - 2.70)
3nd tertile	47.4	11/18	0.38	(0.14 - 1.06)	6/18	0.47	(0.14 - 1.61)	4/18	0.39	(0.09 - 1.77)
Linear trend (10g increase)			*0.83*	*(0.68 - 1.02)*		*0.84*	*(0.66 - 1.07)*		*0.87*	*(0.65 - 1.17)*
Linear trend (1g increase)			*0.98*	*(0.96 - 1.00)*		*0.98*	*(0.96 - 1.01)*		*0.99*	*(0.96 - 1.02)*
**Mushrooms**										
1st tertile (ref.)	0.2	30/24	1.00	-	16/24	1.00	-	12/24	1.00	-
2nd tertile	2.0	9/10	0.63	(0.21 - 1.90)	4/10	0.57	(0.14 - 2.22)	4/10	0.57	(0.13 - 2.54)
3nd tertile	4.0	15/20	0.55	(0.22 - 1.40)	10/20	0.70	(0.24 - 2.04)	2/20	0.13	(0.02 - 0.77)
Linear trend (10g increase)			*0.51*	*(0.11 - 2.36)*		*0.71*	*(0.12 - 4.26)*		*0.06*	*(0.00 - 1.11)*
Linear trend (1g increase)			*0.93*	*(0.80 - 1.09)*		*0.97*	*(0.83 - 1.09)*		*0.76*	*(0.57 - 1.01)*
**Legumes**										
1st tertile (ref.)	6.2	17/18	1.00	-	10/18	1.00	-	6/18	1.00	-
2nd tertile	17.0	16/18	0.93	(0.35 - 2.48)	9/18	0.87	(0.27 - 2.76)	4/18	0.63	(0.14 - 2.90)
3nd tertile	31.8	21/18	1.27	(0.48 - 3.31)	11/18	1.08	(0.35 - 3.32)	8/18	1.35	(0.36 - 5.07)
Linear trend (10g increase)			*0.93*	*(0.76 - 1.15)*		*0.92*	*(0.72 - 1.17)*		*0.94*	*(0.71 - 1.23)*
Linear trend (1g increase)			*0.99*	*(0.97 - 1.01)*		*0.99*	*(0.97 - 1.02)*		*0.99*	*(0.97 - 1.02)*
**Potatoes**										
1st tertile (ref.)	5.3	22/18	1.00	-	13/18	1.00	-	7/18	1.00	-
2nd tertile	12.2	15/18	0.54	(0.20 - 1.45)	7/18	0.40	(0.12 - 1.36)	7/18	0.70	(0.18 - 2.79)
3nd tertile	36.0	17/18	0.70	(0.27 - 1.83)	10/18	0.69	(0.23 - 2.11)	4/18	0.40	(0.09 - 1.85)
Linear trend (10g increase)			*0.88*	*(0.69 - 1.12)*		*0.91*	*(0.69 - 1.19)*		*0.72*	*(0.44 - 1.19)*
Linear trend (1g increase)			*0.99*	*(0.96 - 1.01)*		*0.99*	*(0.96 - 1.02)*		*0.97*	*(0.92 - 1.02)*
**Fresh fruit**										
1st tertile (ref.)	108.3	17/18	1.00	-	10/18	1.00	-	7/18	1.00	-
2nd tertile	218.3	16/18	0.77	(0.28 - 2.12)	11/18	0.93	(0.29 - 2.94)	3/18	0.29	(0.05 - 1.54)
3nd tertile	381.0	21/18	0.97	(0.36 - 2.58)	9/18	0.74	(0.23 - 2.38)	8/18	0.68	(0.17 - 2.64)

Linear trend (10g increase)			*1.00*	*(0.97 - 1.03)*		*0.99*	*(0.96 - 1.02)*		*0.99*	*(0.95 - 1.04)*
Citrus fruit										
1st tertile (ref.)	11.9	21/19	1.00	-	15/19	1.00	-	4/19	1.00	-
2nd tertile	50.0	20/17	0.94	(0.37 - 2.43)	10/17	0.64	(0.21 - 1.89)	8/17	2.17	(0.49 - 9.61)
3nd tertile	97.8	13/18	0.53	(0.19 - 1.46)	5/18	0.28	(0.08 - 1.00)	6/18	1.54	(0.32 - 7.48)
Linear trend (10g increase)			*0.91*	*(0.83 - 1.00)*		*0.87*	*(0.76 - 0.99)*		*0.93*	*(0.82 - 1.07)*
All other fruit										
1st tertile (ref.)	82.7	17/18	1.00	-	9/18	1.00	-	7/18	1.00	-
2nd tertile	174.9	12/18	0.55	(0.19 - 1.57)	9/18	0.74	(0.22 - 2.45)	3/18	0.25	(0.05 - 1.34)
3nd tertile	303.2	25/18	1.23	(0.45 - 3.35)	12/18	1.12	(0.33 - 3.74)	8/18	0.63	(0.15 - 3268)
Linear trend (10g increase)			*1.01*	*(0.98 - 1.05)*		*1.01*	*(0.97 - 1.05)*		*1.00*	*(0.95 - 1.06)*
**Dry fruits, nuts and seeds**										
1st tertile (ref.)	0.3	31/19	1.00	-	15/19	1.00	-	10/19	1.00	-
2nd tertile	1.6	14/18	0.38	(0.15 - 1.00)	7/18	0.41	(0.13 - 1.31)	7/18	0.50	(0.14 - 1.82)
3nd tertile	8.7	9/17	0.24	(0.08 - 0.72)	8/17	0.44	(0.14 - 1.44)	1/17	0.05	(0.00 - 0.64)
Linear trend (1g increase)			*0.89*	*(0.80 - 0.98)*		*0.94*	*(0.85 - 1.05)*		*0.73*	*(0.56 - 0.97)*
Dry fruits										
1st tertile (ref.)	0.0	29/20	1.00	-	18/20	1.00	-	7/20	1.00	-
2nd tertile	0.1	18/22	0.52	(0.21 - 1.28)	8/22	0.34	(0.12 - 1.02)	8/22	1.03	(0.28 - 3.82)
3nd tertile	1.8	7/12	0.23	(0.06 - 0.80)	4/12	0.24	(0.06 - 1.04)	3/12	0.29	(0.05 - 1.76)
Linear trend (1g increase)			*0.74*	*(0.51 - 1.07)*		*0.88*	*(0.60 - 1.27)*		*0.46*	*(0.20 - 1.05)*
Nuts and seeds										
1st tertile (ref.)	0.2	32/20	1.00	-	15/20	1.00	-	11/20	1.00	-
2nd tertile	1.4	13/15	0.43	(0.16 - 1.17)	7/15	0.52	(0.16 - 1.69)	6/15	0.48	(0.12 - 1.88)
3nd tertile	7.1	9/19	0.23	(0.08 - 0.68)	8/19	0.43	(0.14 - 0.36)	1/19	0.06	(0.00 - 0.62)
Linear trend (1g increase)			*0.89*	*(0.79 - 1.00)*		*0.94*	*(0.84 - 1.06)*		*0.74*	*(0.54 - 1.02)*
**Sweets, chocolate, cakes, etc.**										
1st tertile (ref.)	32.9	11/18	1.00	-	6/18	1.00	-	2/18	1.00	-
2nd tertile	75.3	15/18	1.47	(0.50 - 4.38)	9/18	1.53	(0.43 - 5.50)	5/18	2.67	(0.43 - 16.45)
3nd tertile	167.8	28/18	2.61	(0.82 - 8.34)	15/18	2.62	(0.66 - 10.29)	11/18	5.48	(0.85 - 35.26)

Linear trend (10g increase)			*1.00*	*(0.96 - 1.06)*		*1.01*	*(0.96 - 1.07)*		*0.98*	*(0.91 - 1.07)*
Sugar, non-chocolate confectionery										
1st tertile (ref.)	3.1	25/25	1.00	-	13/25	1.00	-	8/25	1.00	-
2nd tertile	23.4	13/13	0.85	(0.31 - 2.30)	6/13	0.82	(0.24 – 2.85)	5/13	0.92	(0.22 - 3.87)
3nd tertile	43.1	16/16	0.79	(0.30 - 2.07)	11/16	1.16	(0.40 - 3.38)	5/16	0.55	(0.13 - 2.38)
Linear trend (10g increase)			*0.98*	*(0.88 - 1.09)*		*1.03*	*(0.92 - 1.15)*		*0.91*	*(0.75 - 1.11)*
Chocolate, candy bars, etc.										
1st tertile (ref.)	0.0	37/33	1.00	-	21/33	1.00	-	12/33	1.00	-
2nd tertile	5.7	8/10	0.77	(0.26 - 2.28)	5/10	0.82	(0.24 - 2.81)	3/10	1.00	(0.20 - 4.93)
3nd tertile	20.0	9/11	0.83	(0.28 - 2.49)	4/11	0.61	(0.15 - 2.42)	3/11	0.94	(0.27 - 4.76)
Linear trend (10g increase)			*0.70*	*(0.45 - 1.08)*		*0.69*	*(0.41 - 1.17)*		*0.71*	*(0.37 - 1.38)*
Linear trend (1g increase)			*0.96*	*(0.92 - 1.01)*		*0.96*	*(0.91 - 1.02)*		*0.97*	*(0.90 - 1.03)*
Ice-cream										
1st tertile (ref.)	2.5	18/24	1.00	-	12/24	1.00	-	4/24	1.00	-
2nd tertile	10.7	12/15	1.37	(0.48 - 3.87)	8/15	1.20	(0.38 - 3.76)	3/15	1.73	(0.30 - 10.06)
3nd tertile	32.1	24/15	2.69	(1.00 - 7.22)	10/15	1.41	(0.45 - 4.47)	11/15	7.35	(1.53 - 35.38)
Linear trend (10g increase)			*1.15*	*(0.97 - 1.37)*		*1.12*	*(0.94 - 1.34)*		*1.20*	*(0.91 - 1.58)*
Linear trend (1g increase)			*1.01*	*(1.00 - 1.03)*		*1.01*	*(0.99 - 1.03)*		*1.02*	*(0.99 - 1.05)*
Cakes, pies and pastries										
1st tertile (ref.)	6.0	13/22	1.00	-	8/22	1.00	-	3/22	1.00	-
2nd tertile	26.5	11/16	1.26	(0.43 - 3.66)	6/16	1.09	(0.30 - 3.88)	4/16	2.43	(0.42 - 13.96)
3nd tertile	88.5	30/16	3.16	(1.15 - 8.69)	16/16	2.95	(0.88 - 9.89)	11/16	5.28	(1.09 - 25.70)
Linear trend (10g increase)			*0.99*	*(0.93 - 1.05)*		*0.98*	*(0.91 - 1.06)*		*0.99*	*(0.91 - 1.07)*
Biscuits, dry cakes										
1st tertile (ref.)	0.0	24/28	1.00	-	11/28	1.00	-	10/28	1.00	-
2nd tertile	12.0	9/13	1.04	(0.36 - 3.04)	4/13	0.98	(0.25 - 3.87)	3/13	0.90	(0.19 - 4.21)
3nd tertile	30.0	21/13	1.72	(0.63 - 4.66)	15/13	3.12	(1.02 - 9.59)	5/13	0.68	(0.15 - 3.11)
Linear trend (10g increase)			*1.19*	*(0.94 - 1.51)*		*1.31*	*(0.99 - 1.75)*		*1.10*	*(0.82 - 1.47)*

Linear trend (1g increase)			*1.02*	*(0.99 - 1.04)*		*1.03*	*(1.00 - 1.06)*		*1.01*	*(0.98 - 1.04)*
**Oils and fats**										
1st tertile (ref.)	14.6	25/18	1.00	-	14/18	1.00	-	8/18	1.00	-
2nd tertile	21.9	10/18	0.37	(0.13 - 1.03)	5/18	0.33	(0.09 - 1.15)	3/18	0.33	(0.07 - 1.58)
3rd tertile	35.7	19/18	0.58	(0.21 - 1.61)	11/18	0.65	(0.21 - 2.05)	7/18	0.62	(0.59 - 2.49)
Linear trend (10g increase)			*0.66*	*(0.46 - 0.97)*		*0.70*	*(0.45 - 1.07)*		*0.65*	*(0.38 - 1.12)*
Linear trend (1g increase)			*0.96*	*(0.92 - 1.00)*		*0.96*	*(0.92 - 1.01)*		*0.96*	*(0.91 - 1.01)*
Vegetable fats and non-olive oils										
1st tertile (ref.)	0.0	31/28	1.00	-	21/28	1.00	-	7/28	1.00	-
2nd tertile	2.4	13/12	0.99	(0.37 - 2.66)	3/12	0.28	(0.07 - 1.22)	9/12	4.54	(1.10 - 18.76)
3rd tertile	8.5	10/14	0.77	(0.27 - 2.21)	6/14	0.62	(0.19 - 2.08)	2/14	0.89	(0.13 - 5.99)
Linear trend (10g increase)			*0.39*	*(0.11 - 1.38)*		*0.39*	*(0.08 - 1.84)*		*0.42*	*(0.07 - 2.52)*
Linear trend (1g increase)			*0.91*	*(0.80 - 1.03)*		*0.91*	*(0.78 - 1.06)*		*0.92*	*(0.77 - 1.10)*
Olive oil										
1st tertile (ref.)	9.6	19/18	1.00	-	11/18	1.00	-	4/18	1.00	-
2nd tertile	17.4	15/18	0.72	(0.26 - 1.94)	8/18	0.65	(0.20 - 2.10)	7/18	1.61	(0.35 - 7.32)
3rd tertile	29.1	20/18	0.79	(0.29 - 2.18)	11/18	0.76	(0.23 - 2.46)	7/18	1.26	(0.26 - 5.97)
Linear trend (10g increase)			*0.73*	*(0.50 - 1.06)*		*0.77*	*(0.50 - 1.17)*		*0.73*	*(0.43 - 1.22)*
Linear trend (1g increase)			*0.97*	*(0.93 - 1.01)*		*0.97*	*(0.93 - 1.02)*		*0.97*	*(0.92 - 1.02)*
Butter and other animal fats										
1st tertile (ref.)	0.1	16/22	1.00	-	8/22	1.00	-	7/22	1.00	-
2nd tertile	1.2	24/16	2.22	(0.85 - 5.80)	14/16	2.41	(0.78 - 7.45)	8/16	1.61	(0.42 - 6.19)
3rd tertile	3.4	14/16	1.23	(0.42 - 3.56)	8/16	1.31	(0.37 - 4.64)	3/16	0.48	(0.09 - 2.57)
Linear trend (10g increase)			*0.78*	*(0.26 - 2.36)*		*0.76*	*(0.20 - 2.95)*		*0.76*	*(0.16 - 3.56)*
Linear trend (1g increase)			*0.98*	*(0.87 - 1.09)*		*0.97*	*(0.85 - 1.11)*		*0.97*	*(0.83 - 1.14)*
**Beverages**										
Coffee and tea										
1st tertile (ref.)	47.9	27/19	1.00	-	13/19	1.00	-	11/19	1.00	-
2nd tertile	90.0	11/17	0.46	(0.17 - 1.24)	5/17	0.46	(0.13 - 1.59)	4/17	0.38	(0.09 - 1.55)
3rd tertile	196.4	16/18	0.56	(0.21 - 1.46)	12/18	0.93	(0.32 - 2.70)	3/18	0.18	(0.03 - 0.97)
Linear trend (10g increase)			*0.99*	*(0.96 - 1.02)*		*1.01*	*(0.98 - 1.04)*		*0.85*	*(0.75 - 0.96)*
Coffee										

nutrients

MDPI

1st tertile (ref.)	25.7	22/19	1.00	-	10/19	1.00	-	8/19	1.00	-
2nd tertile	67.8	12/14	0.77	(0.27 - 2.19)	8/14	1.03	(0.31 - 3.43)	3/14	0.50	(0.09 - 2.71)
3nd tertile	100.0	20/21	0.71	(0.29 - 1.78)	12/21	1.03	(0.35 - 3.04)	7/21	0.63	(0.18 - 2.26)
Linear trend (10g increase)			*0.99*	*(0.92 - 1.06)*		*1.02*	*(0.95 - 1.10)*		*0.95*	*(0.83 - 1.08)*
Tea										
1st tertile (ref.)	0.0	40/36	1.00	-	19/36	1.00	-	17/36	1.00	-
2nd tertile	42.9	7/7	0.78	(0.24 - 2.54)	5/7	1.13	(0.30 - 4.22)	1/7	0.23	(0.02 - 2.33)
3nd tertile	150.0	7/11	0.61	(0.19 - 1.93)	6/11	1.09	(0.31 - 3.91)	0/11	-	-
Linear trend (10g increase)			*0.99*	*(0.96 - 1.02)*		*0.99*	*(0.96 - 1.02)*		*0.68*	*(0.41 - 1.13)*
Wine										
1st tertile (ref.)	0	33/33	1.00	-	19/33	1.00	-	10/33	1.00	-
2nd tertile	53.6	1/7	0.17	(0.02 - 1.47)	1/7	0.28	(0.03 - 2.52)	0/7	-	-
3nd tertile	250.0	20/14	1.31	(0.54 - 3.16)	10/14	1.35	(0.46 - 4.00)	8/14	1.60	(0.49 - 5.21)
Linear trend (10g increase)			*0.99*	*(0.97 - 1.02)*		*0.99*	*(0.96 - 1.03)*		*1.00*	*(0.97 - 1.04)*
Red wine										
1st tertile (ref.)	0.0	38/37	1.00	-	21/37	1.00	-	13/37	1.00	-
2nd tertile	31.7	3/9	0.38	(0.09 - 1.56)	1/9	0.22	(0.03 - 1.91)	1/9	0.37	(0.04 - 3.43)
3nd tertile	125.0	13/8	1.70	(0.58 - 5.00)	8/8	2.39	(0.67 - 8.58)	4/8	1.26	(0.28 - 5.61)
Linear trend (10g increase)			*1.02*	*(0.97 - 1.08)*		*1.04*	*(0.98 - 1.11)*		*1.00*	*(0.91 - 1.10)*
White wine										
1st tertile (ref.)	0.0	38/37	1.00	-	22/37	1.00	-	12/37	1.00	-
2nd tertile	44.6	14/9	1.50	(0.55 - 4.07)	8/9	1.49	(0.48 - 4.62)	3/9	1.59	(0.38 - 6.58)
3nd tertile	236.1	2/8	0.20	(0.04 - 1.10)	0/8	-	-	2/8	0.61	(0.10 - 3.73)
Linear trend (10g increase)			*0.99*	*(0.95 - 1.02)*		*0.95*	*(0.88 - 1.02)*		*1.00*	*(0.97 - 1.04)*
Aperitif wines and beers										
1st tertile (ref.)	0.0	44/33	1.00	-	22/33	1.00	-	16/33	1.00	-
2nd tertile	6.8	6/11	0.40	(0.12 - 1.30)	5/11	0.69	(0.20 - 2.44)	1/11	0.10	(0.01 - 1.13)
3nd tertile	94.3	4/10	0.25	(0.06 - 1.01)	3/10	0.41	(0.08 - 2.07)	1/10	0.12	(0.01 - 1.42)
Linear trend (10g increase)			*0.95*	*(0.90 - 1.01)*		*0.94*	*(0.86 - 1.02)*		*0.96*	*(0.90 - 1.03)*
Spirits and liqueurs										
1st tertile (ref.)	0.0	48/36	1.00	-	27/36	1.00	-	16/36	1.00	-
2nd tertile	0.7	5/11	0.28	(0.08 - 0.96)	2/11	0.19	(0.03 - 1.07)	2/11	0.29	(0.05 - 1.76)
3nd tertile	5.3	1/7	0.11	(0.01 - 1.15)	1/7	0.22	(0.02 - 2.22)	0/7	-	-

nutrients

MDPI

Linear trend (1g increase)			*0.42*	*(0.16 - 1.09)*		*0.52*	*(0.19 - 1.41)*		*0.16*	*(0.01 - 1.88)*
Fruit juices										
1st tertile (ref.)	0.0	30/38	1.00	-	17/38	1.00	-	10/38	1.00	-
2nd tertile	89.3	12/8	2.50	(0.84 - 7.43)	7/8	2.44	(0.70 - 8.51)	3/8	2.14	(0.41 - 11.13)
3nd tertile	272.0	12/8	1.94	(0.66 - 5.71)	6/8	1.89	(0.51 - 6.97)	5/8	2.62	(0.60 - 11.56)
Linear trend (10g increase)			*1.02*	*(0.99 - 1.04)*		*1.02*	*(0.98 - 1.05)*		*1.03*	*(0.99 - 1.07)*
Soft drinks										
1st tertile (ref.)	0.0	43/42	1.00	-	24/42	1.00	-	15/42	1.00	-
2nd tertile	71.4	5/4	1.67	(0.38 - 7.40)	3/4	1.72	(0.33 - 9.05)	2/4	1.91	(0.26 - 13.92)
3nd tertile	400.0	6/8	0.62	(0.18 - 2.18)	3/8	0.69	(0.15 - 3.22)	1/8	0.17	(0.01 - 2.21)
Linear trend (10g increase)			*0.98*	*(0.95 - 1.01)*		*0.98*	*(0.94 - 1.02)*		*0.95*	*(0.88 - 1.02)*
Alcohol										
1st tertile (ref.)	0.3	22/22	1.00	-	13/22	1.00	-	6/22	1.00	-
2nd tertile	3.5	14/16	0.81	(0.30 - 2.21)	8/16	0.78	(0.25 - 2.48)	5/16	1.13	(0.27 - 4.78)
3nd tertile	23.8	16/16	1.04	(0.37 - 2.93)	9/16	1.01	(0.28 - 3.65)	7/16	1.37	(0.32 - 5.79)
Linear trend (1g increase)			*0.99*	*(0.97 - 1.02)*		*0.99*	*(0.95 - 1.02)*		*1.01*	*(0.98 - 1.03)*

[a]Adjusted model for sex, age, educational attainment, and energy intake; Abbreviations: CI, confidence interval; OR: odds ratio.

nutrients

MDPI

Supplemental Figures S1-S10. Spline regression analysis of risk of early-onset Alzheimer's dementia (EO-AD) for an increasing intake of food: Cereals and cereal products (1); meats and meat products (2); milk, dairy products, and eggs (3); fish and seafood (4); vegetables (5); mushrooms, legumes, and potatoes (6); fresh and dry fruits (7); sweets, chocolate, cakes, etc. (8); oils and fats (9); beverages (10). The black line indicates the odds ratio for dementia risk; the dash gray lines are 95% confidence limits; the reference line at 1.0 with black spikes indicates the distribution of intake of participants. Note: Spline analysis was not possible for offal and most beverages due to a few subjects reporting consumption which is different from the null value (tea, red, white, aperitif wines and beers, spirits and soft drinks).

Supplemental Figure S1. Cereals and cereal products

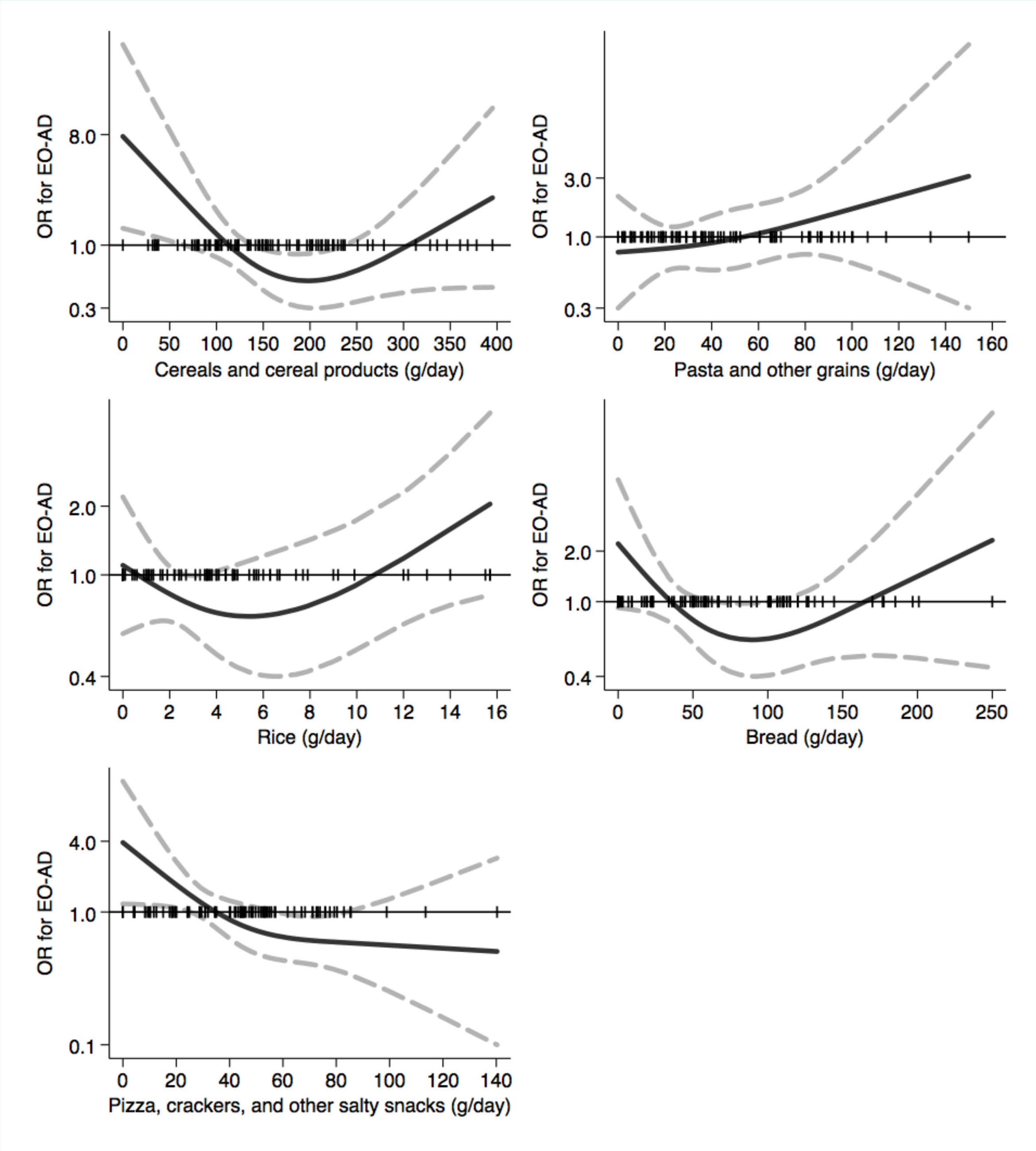

nutrients

MDPI

Supplemental Figure S2. Meat products

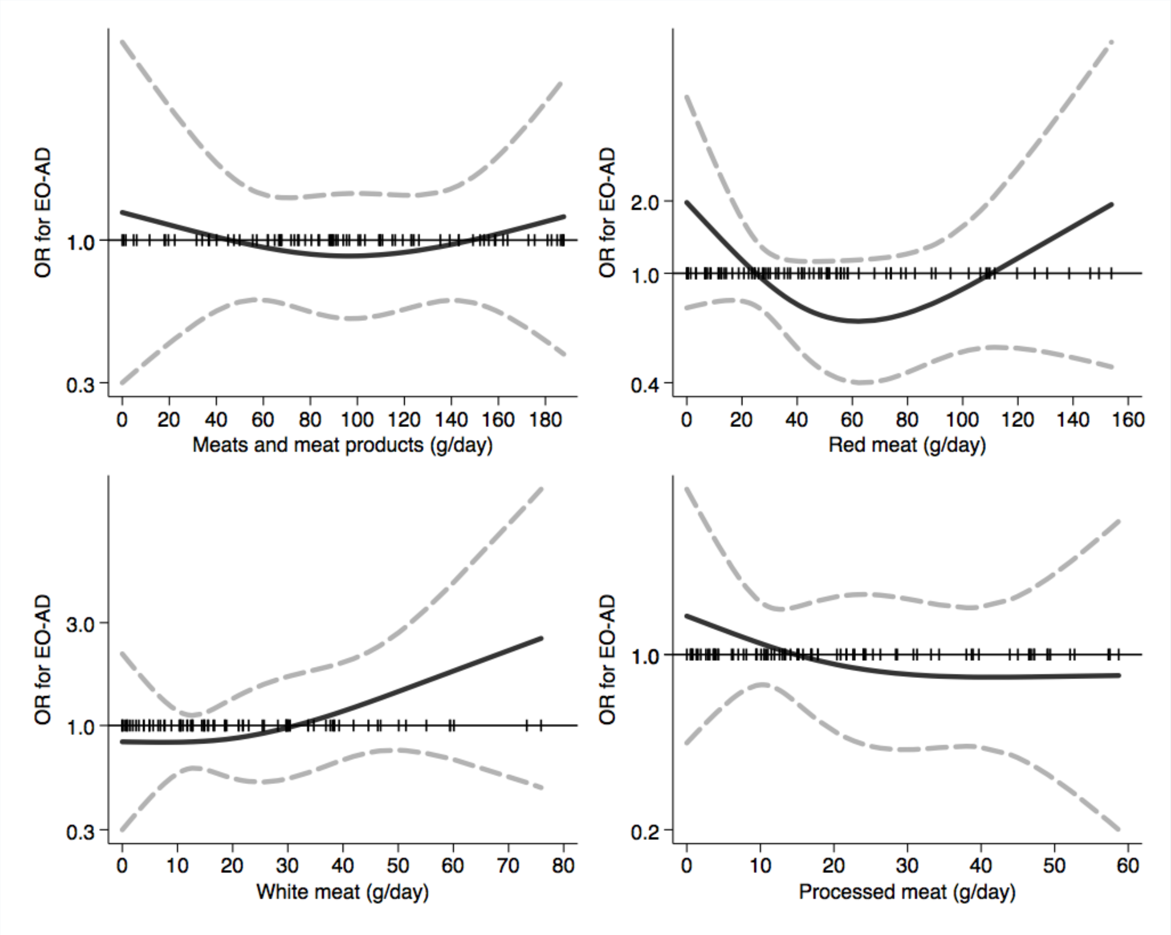

nutrients

MDPI

Supplemental Figure S3. Dairy products and eggs

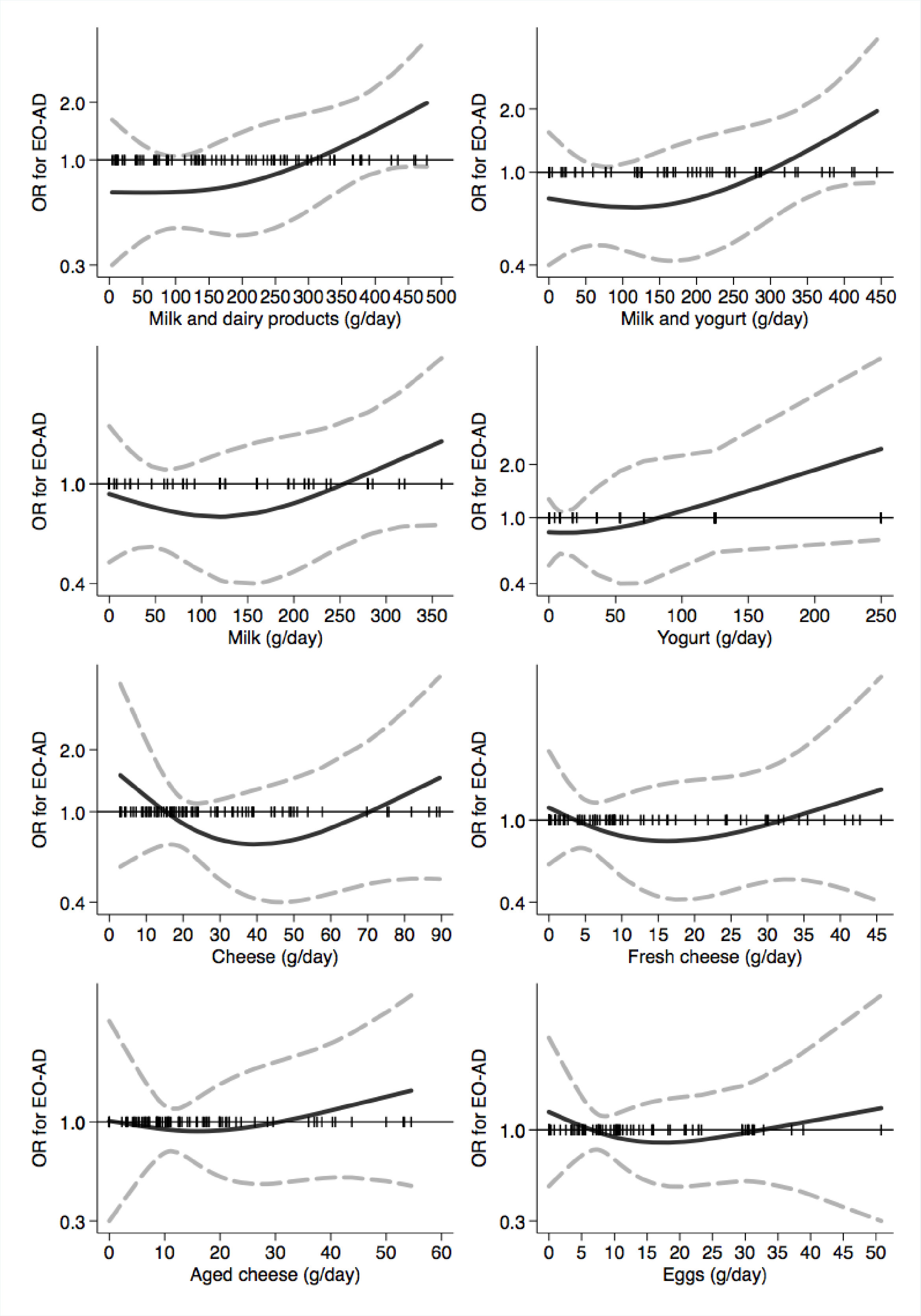

nutrients

MDPI

Supplemental Figure S4. Fish and seafood

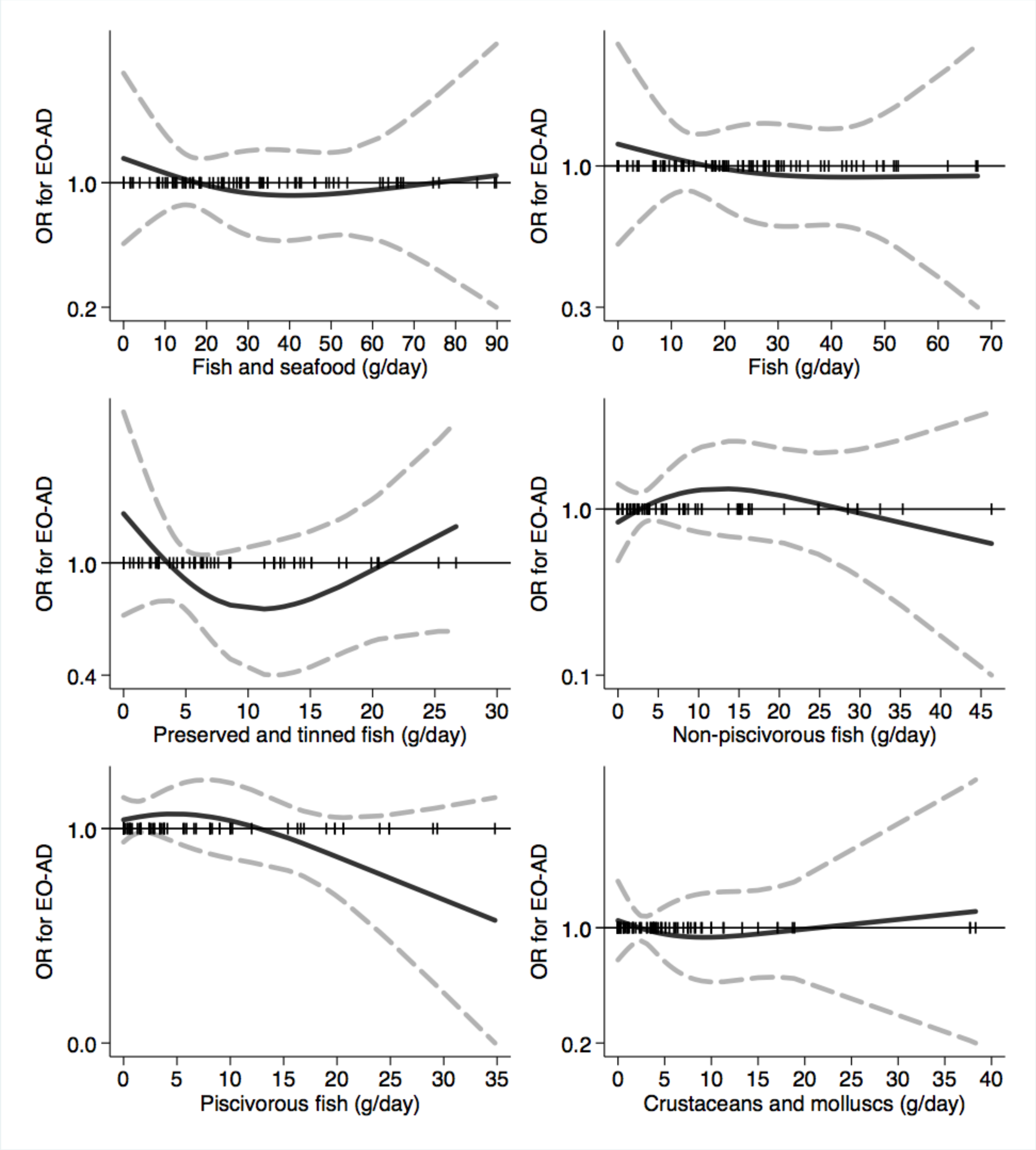

Supplemental Figure S5. Vegetables

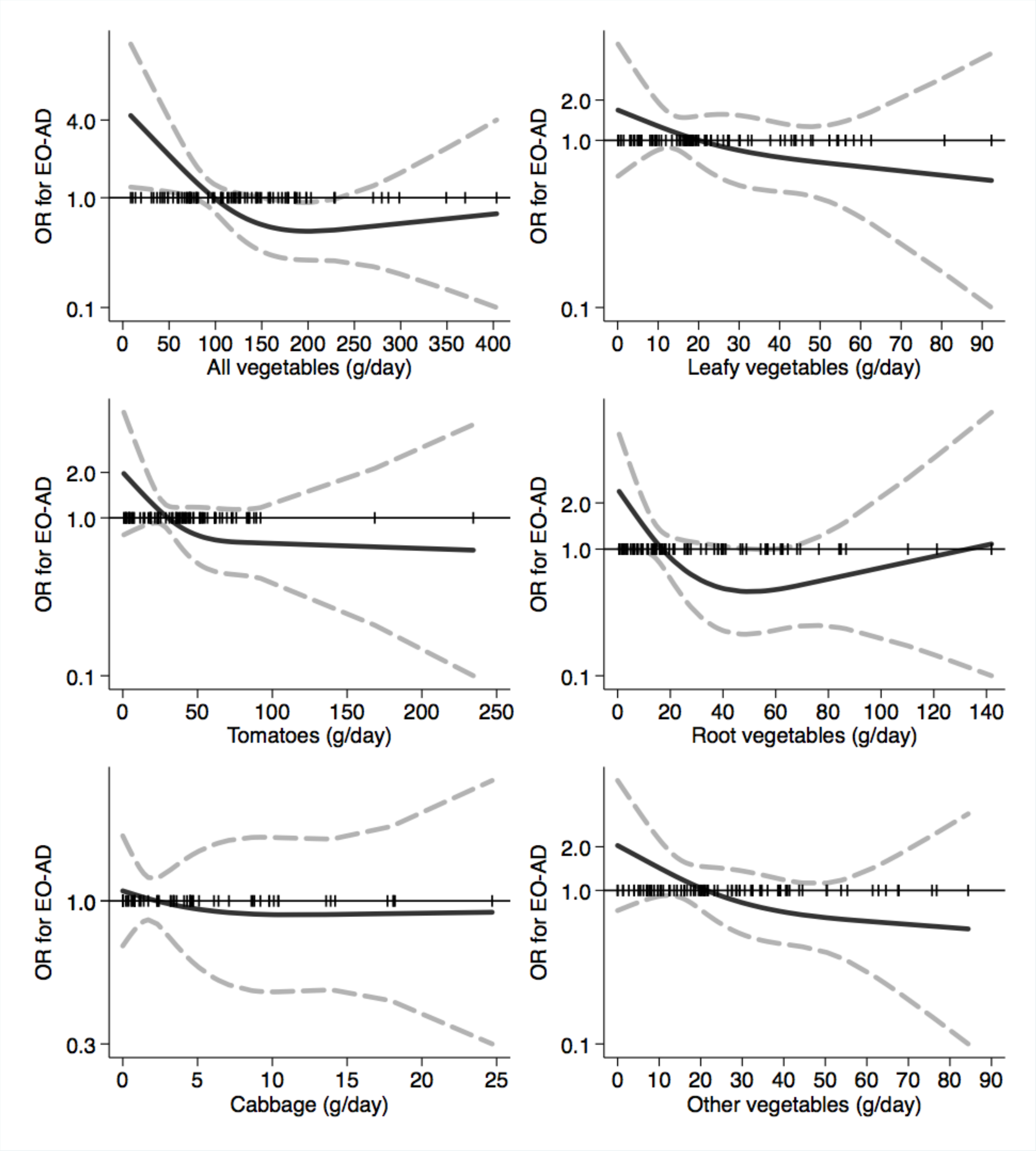

nutrients

MDPI

Supplemental Figure S6. Mushrooms, legumes and potatoes

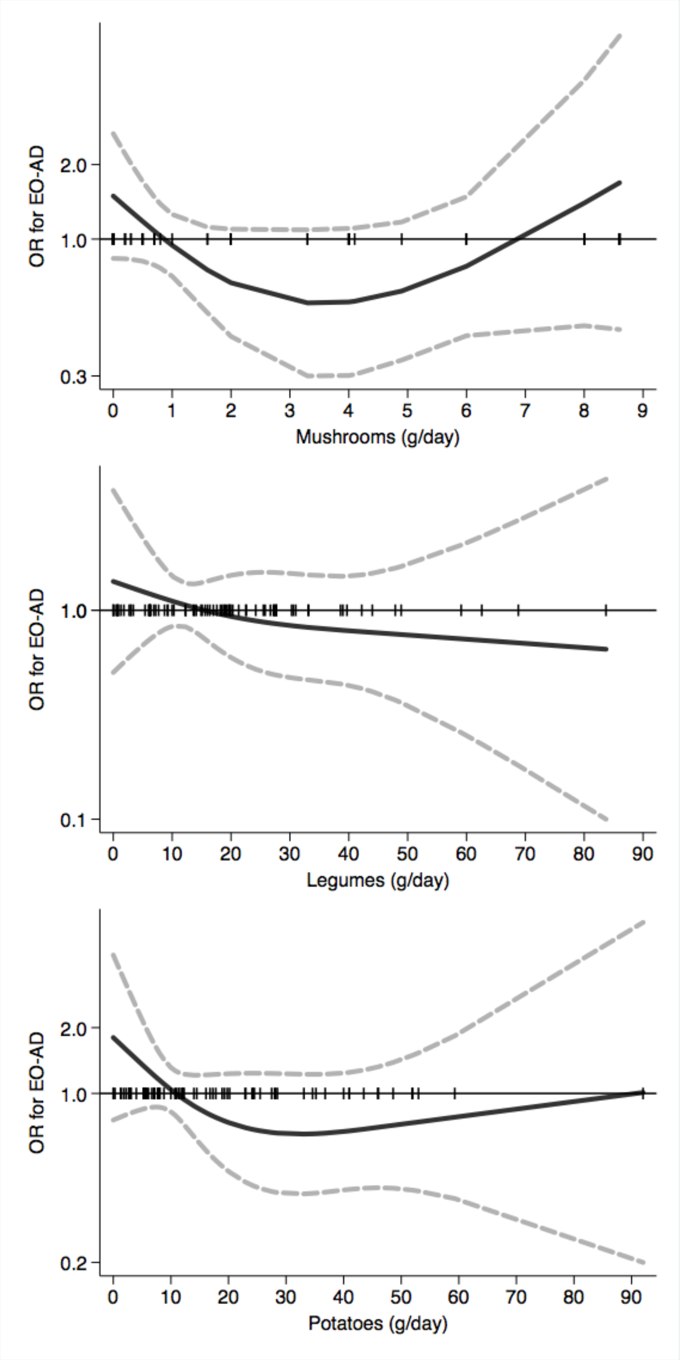

nutrients

MDPI

Supplemental Figure S7. Fresh and dry fruits

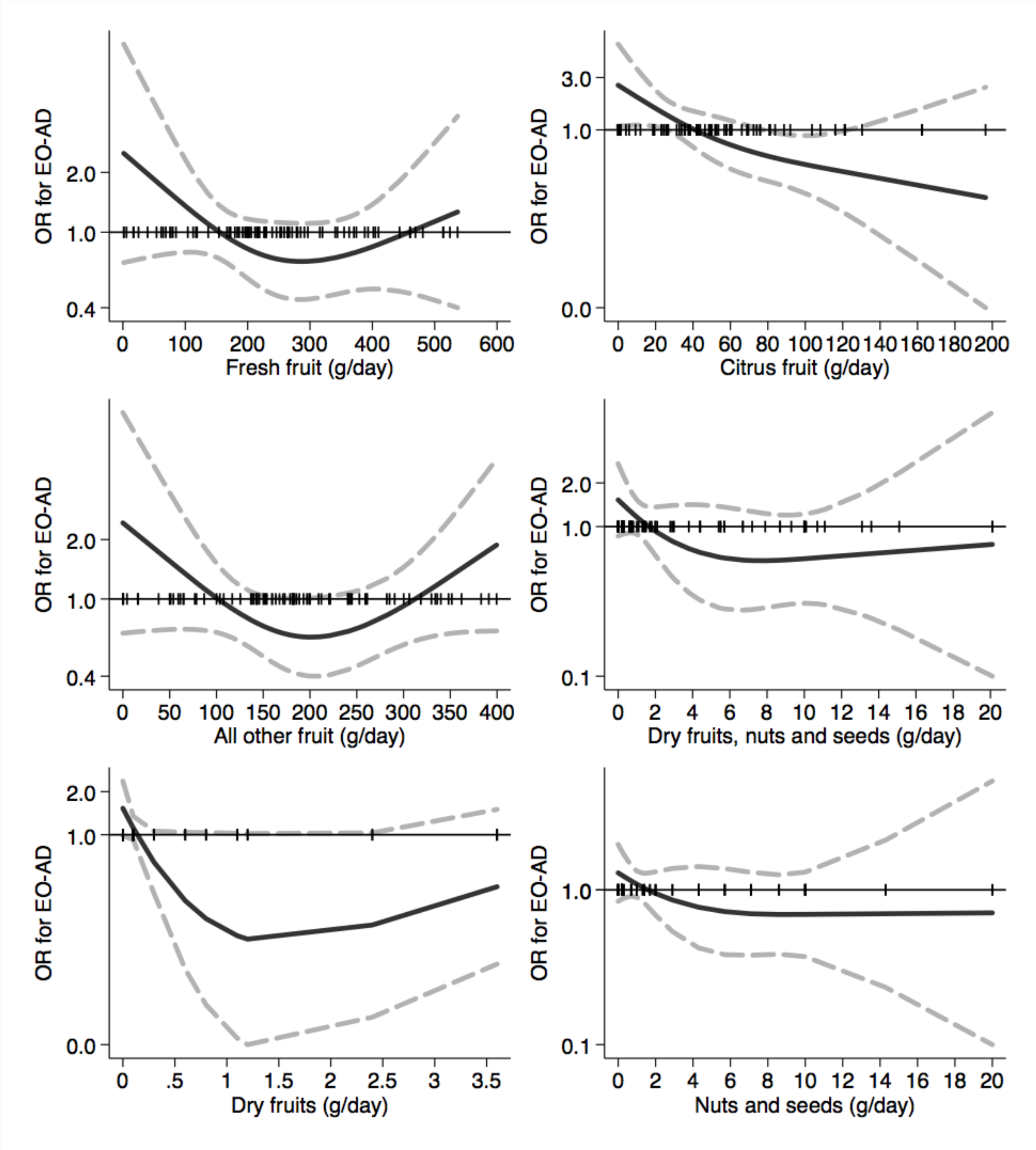

Supplemental Figure S8. Sweets, chocolate, cakes, etc.

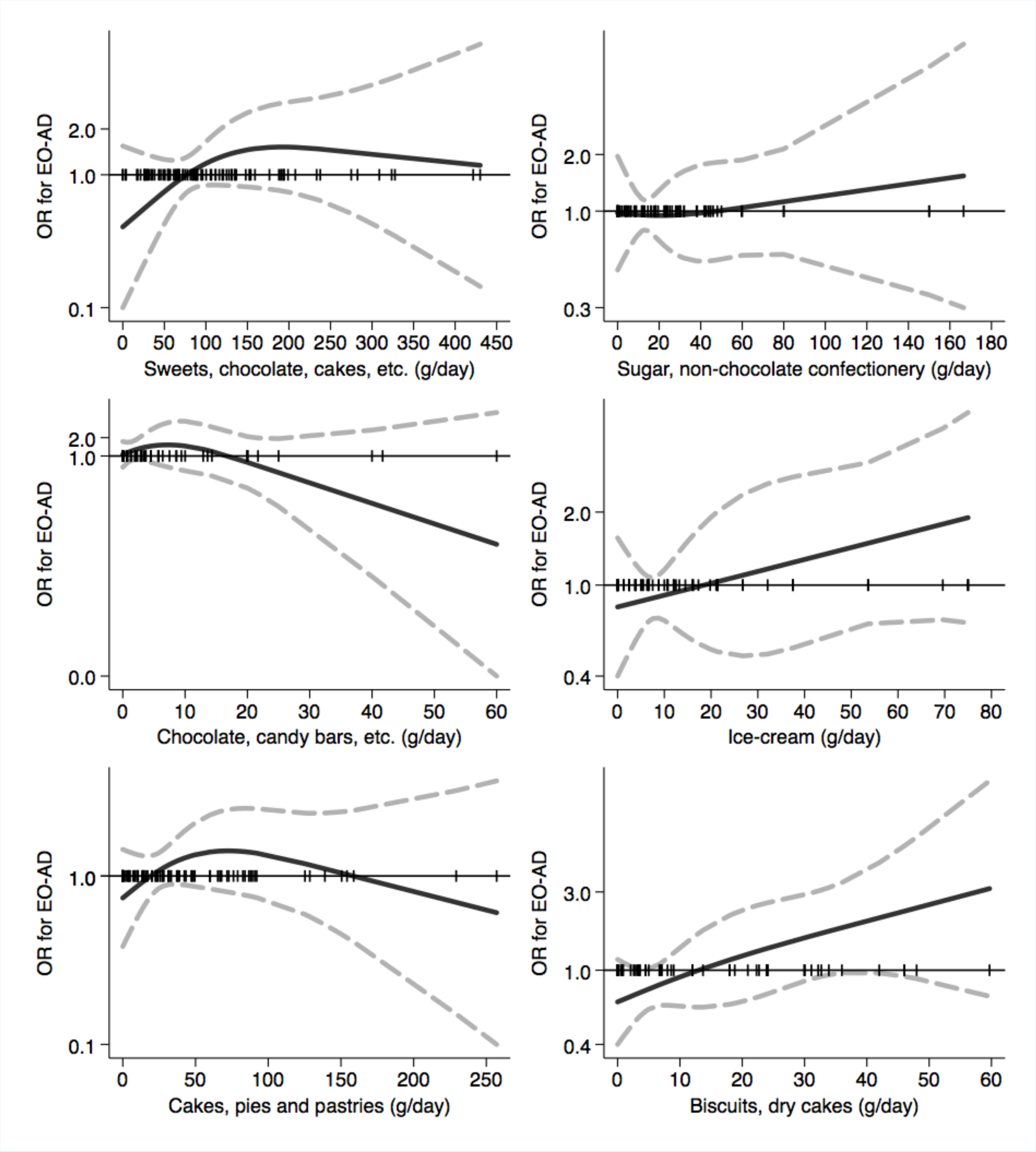

nutrients

MDPI

Supplemental Figure S9. Oils and fats

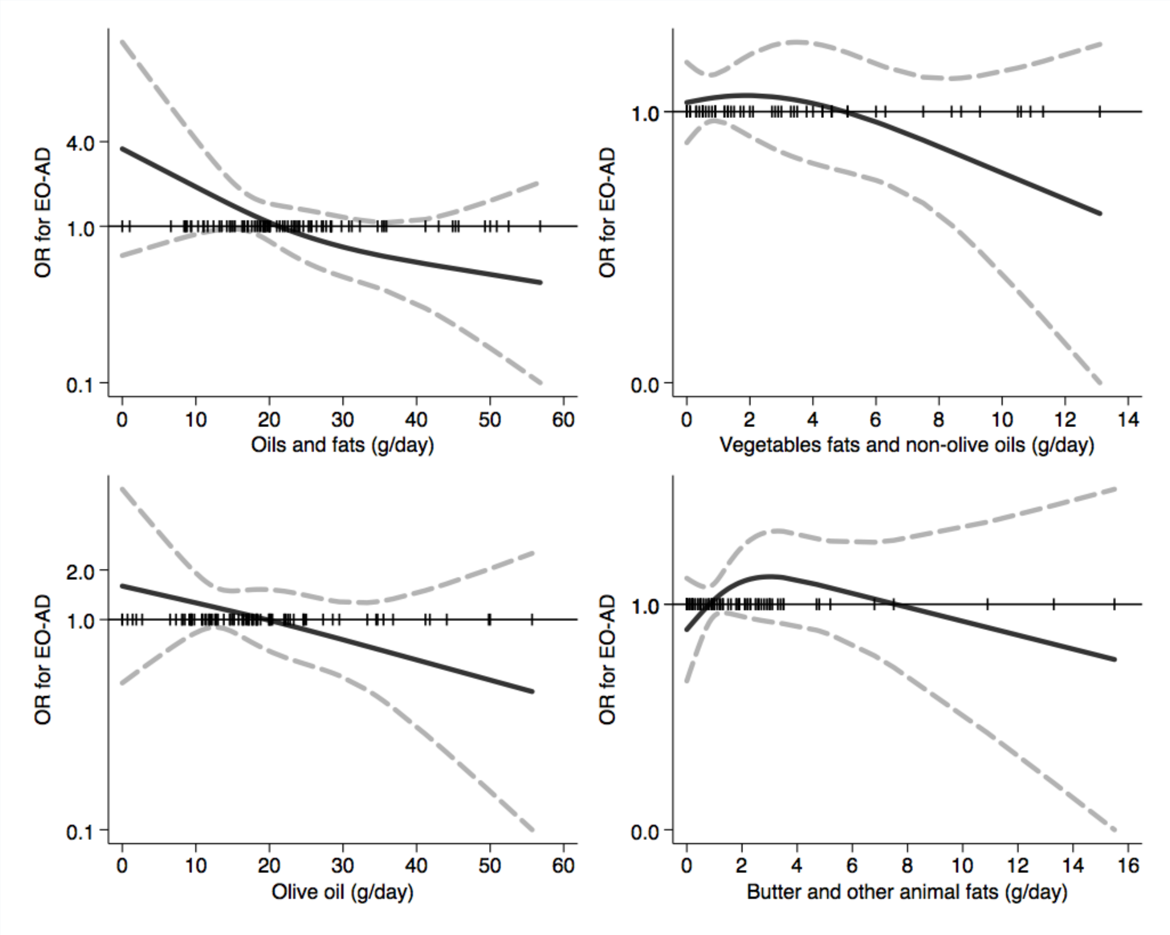

nutrients

MDPI

Supplemental Figure S10. Beverages

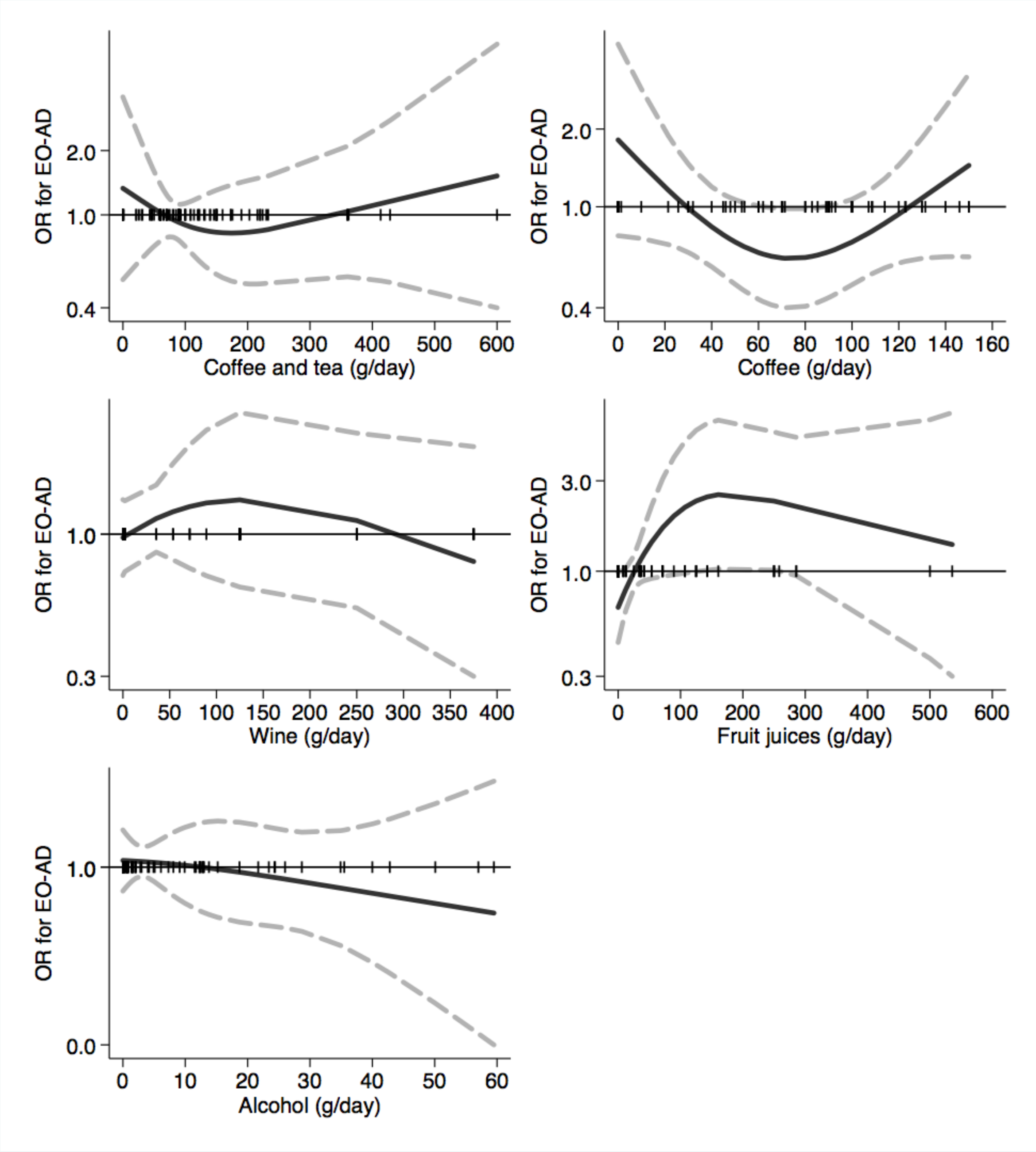

Supplemental Figures S11-S20. Spline regression analysis of risk of early-onset frontotemporal dementia spectrum (EO-FTD) for an increasing intake of food: Cereals and cereal products (1); meats and meat products (2); milk, dairy products, and eggs (3); fish and seafood (4); vegetables (5); mushrooms, legumes, and potatoes (6); fresh and dry fruits (7); sweets, chocolate, cakes, etc. (8); oils and fats (9); beverages (10). The black line indicates the odds ratio for dementia risk; the dash gray lines are 95% confidence limits; the reference line at 1.0 with black spikes indicates the distribution of intake of participants. Note: Spline analysis was not possible for offal and most beverages due to a few subjects reporting consumption which is different from the null value (tea, red, white, aperitif wines and beers, spirits, fruit juices, and soft drinks).

Supplemental Figure S11. Cereals and cereal products

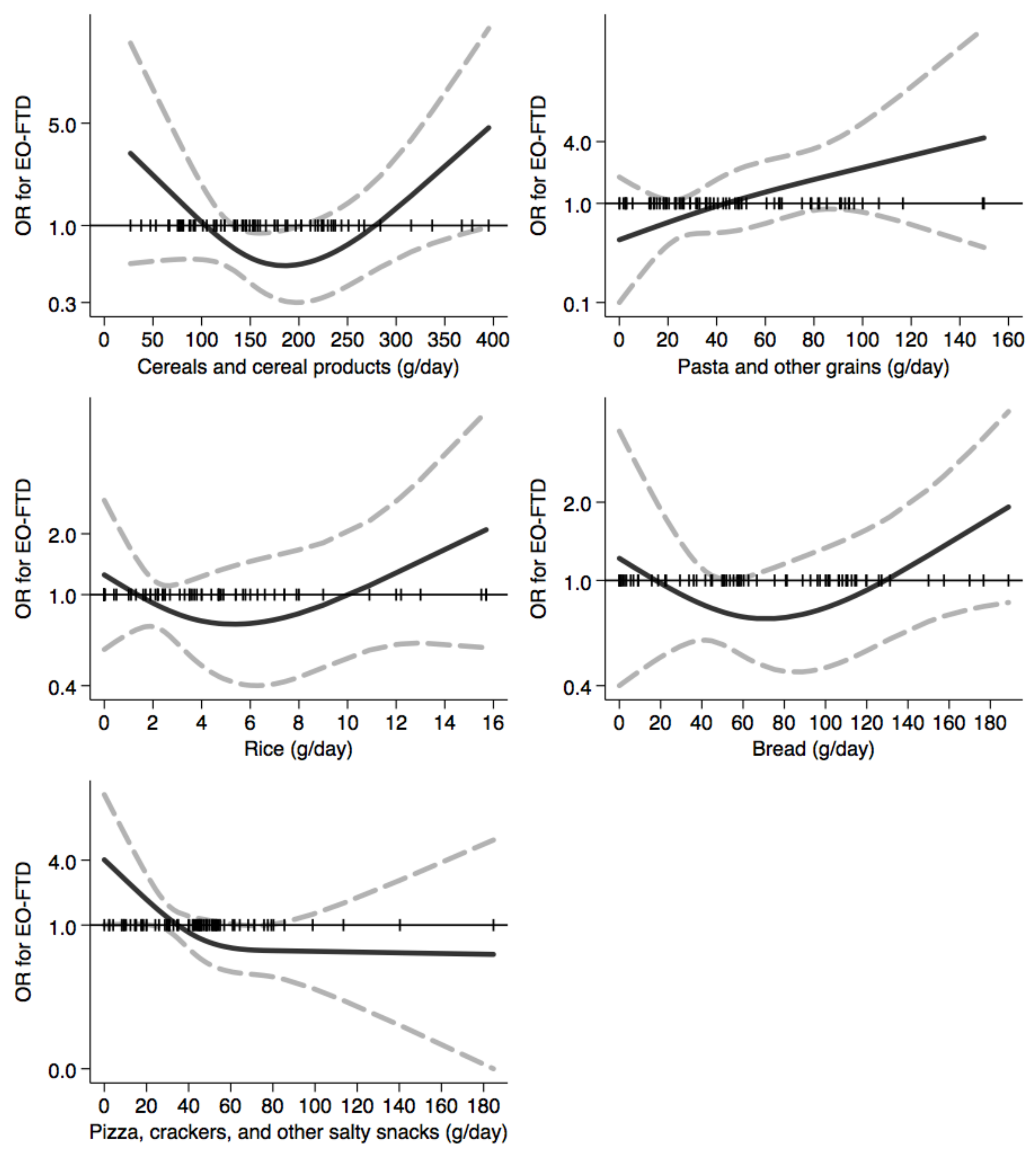

nutrients

MDPI

Supplemental Figure S12. Meats and meat products

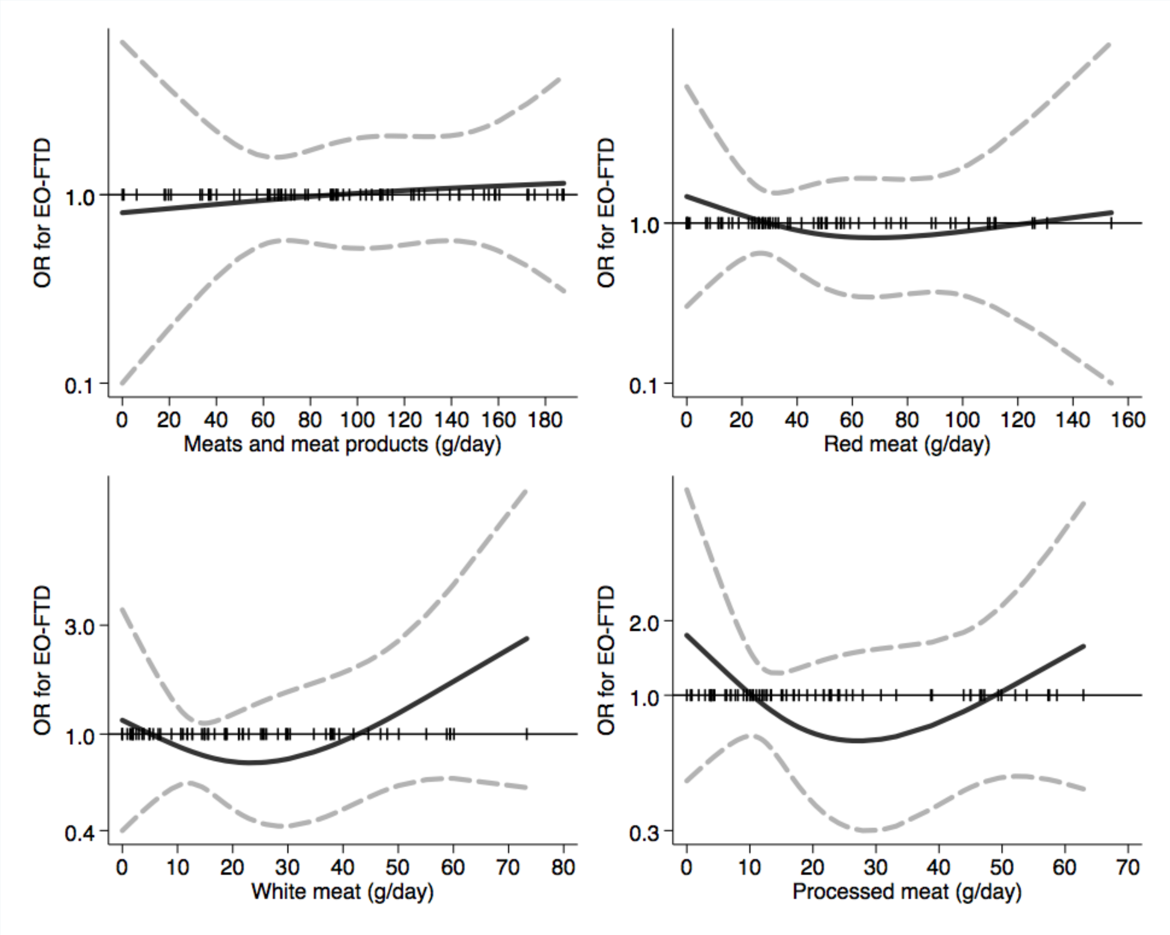

nutrients

Supplemental Figure S13. Milk, dairy products and eggs

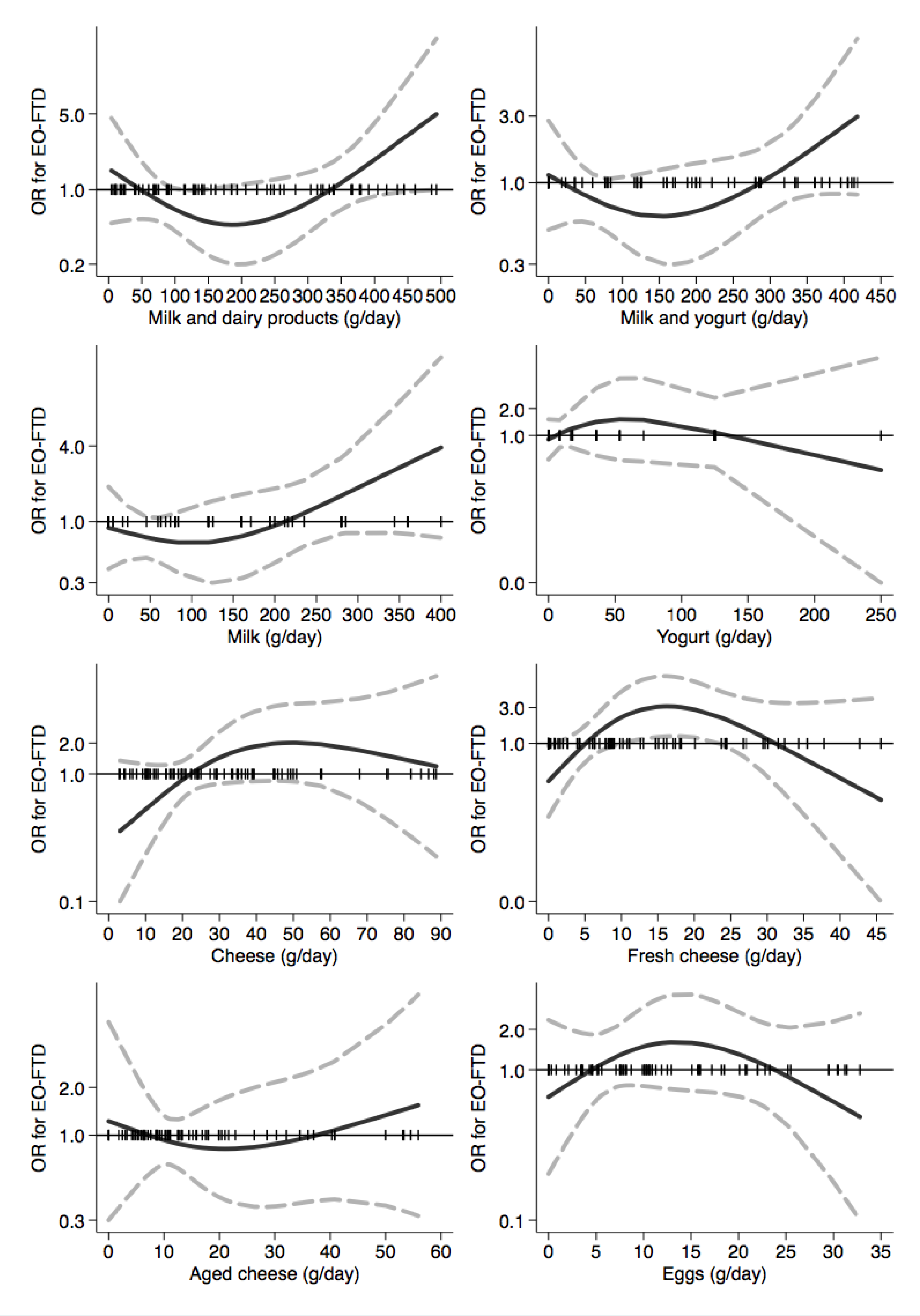

nutrients

MDPI

Supplemental Figure S14. Fish and seafood

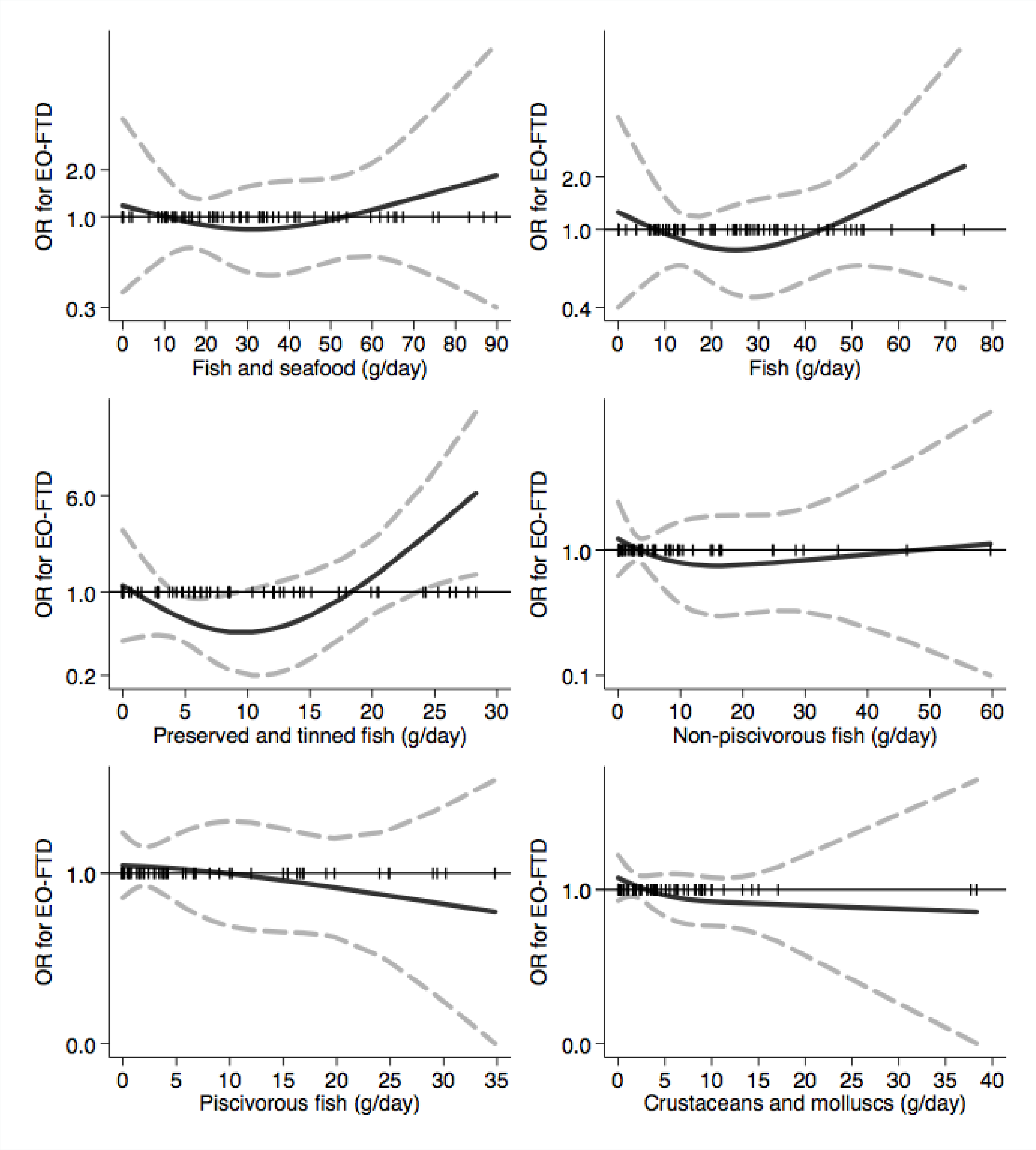

nutrients

MDPI

Supplemental Figure S15. Vegetables

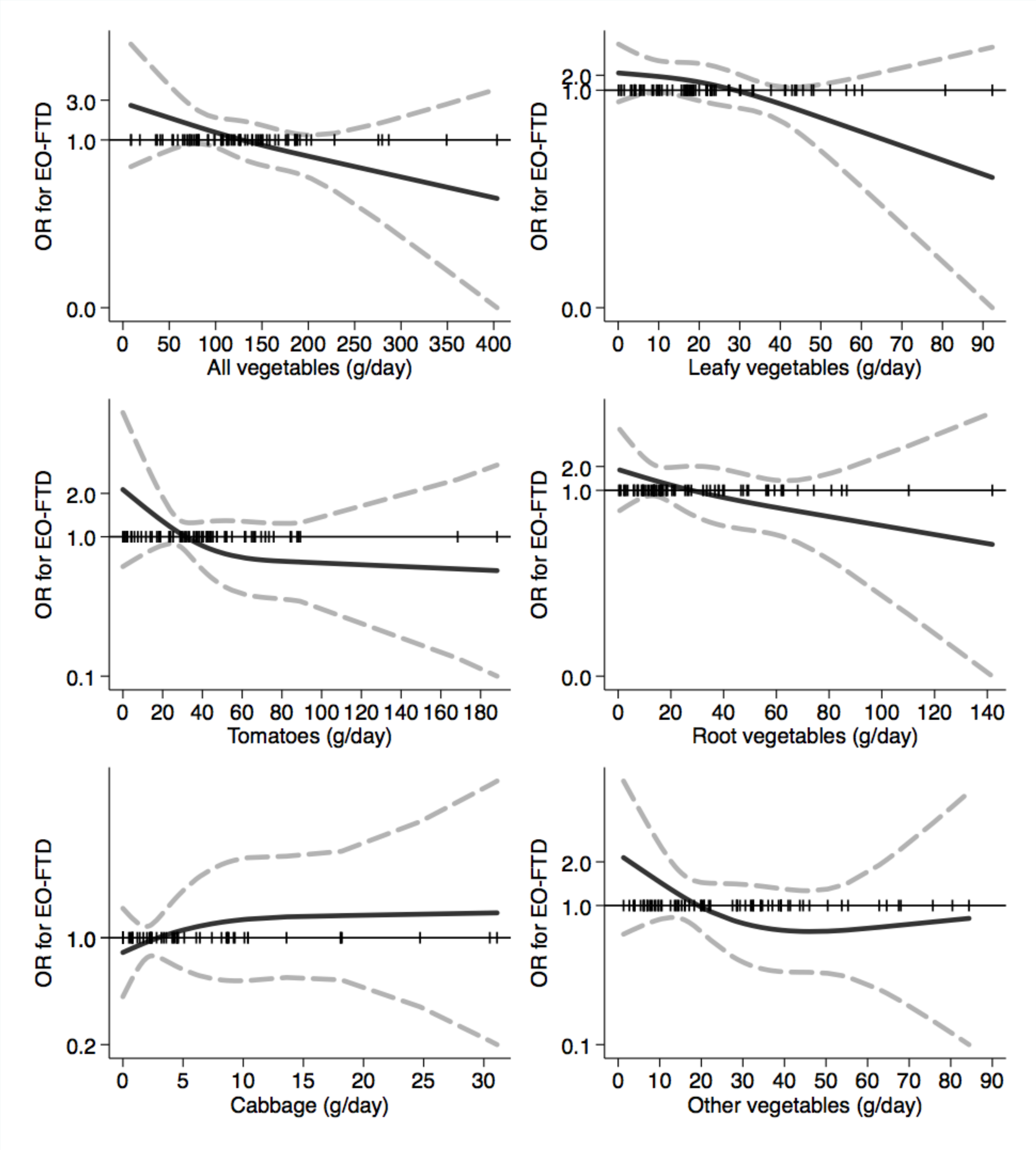

nutrients

Supplemental Figure S16. Mushrooms, legumes and potatoes

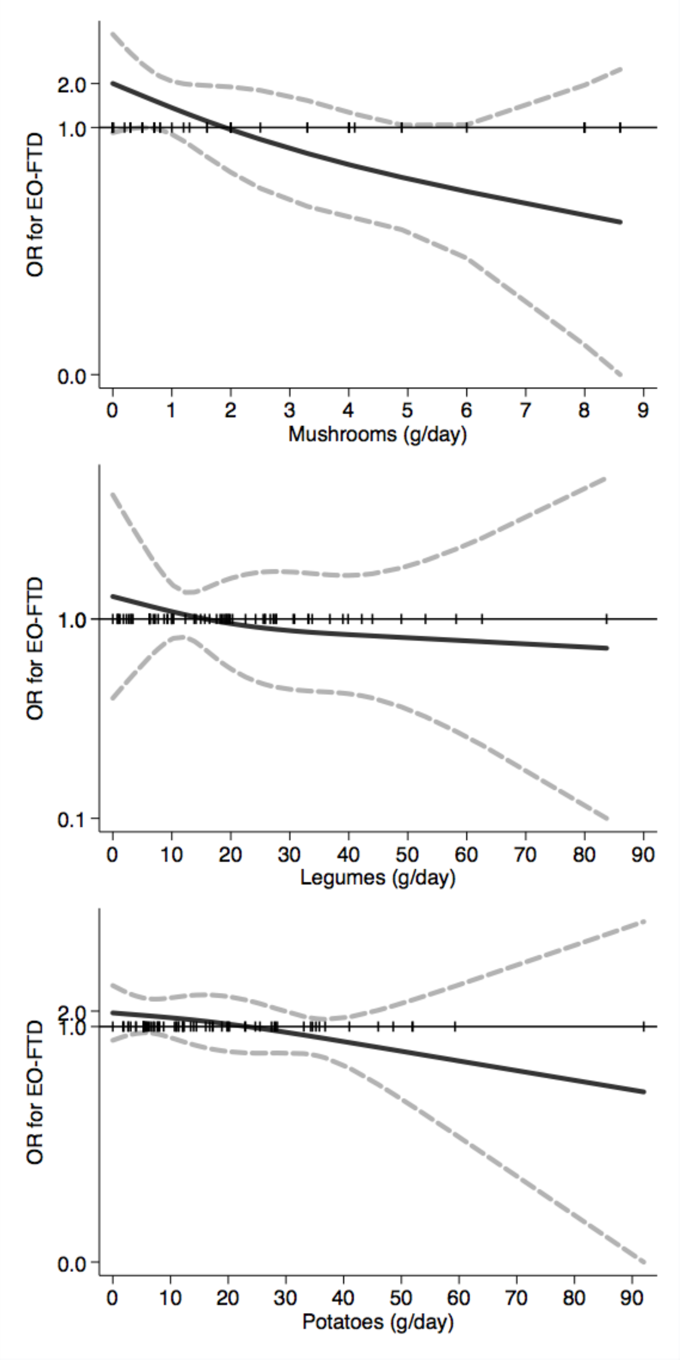

nutrients

MDPI

Supplemental Figure S17. Fresh and dry fruits

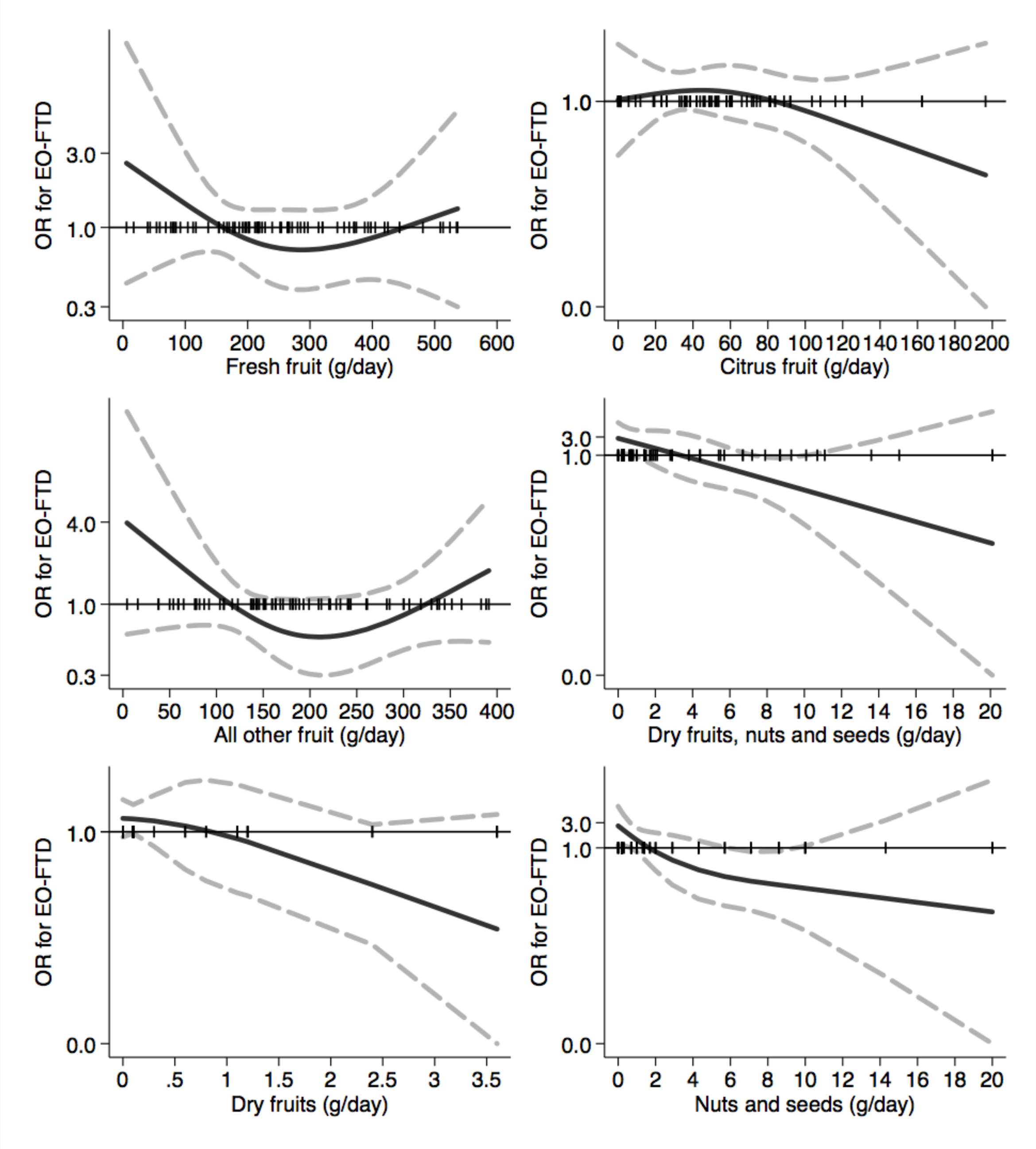

Supplemental Figure S18. Sweets, chocolate, cakes, etc.

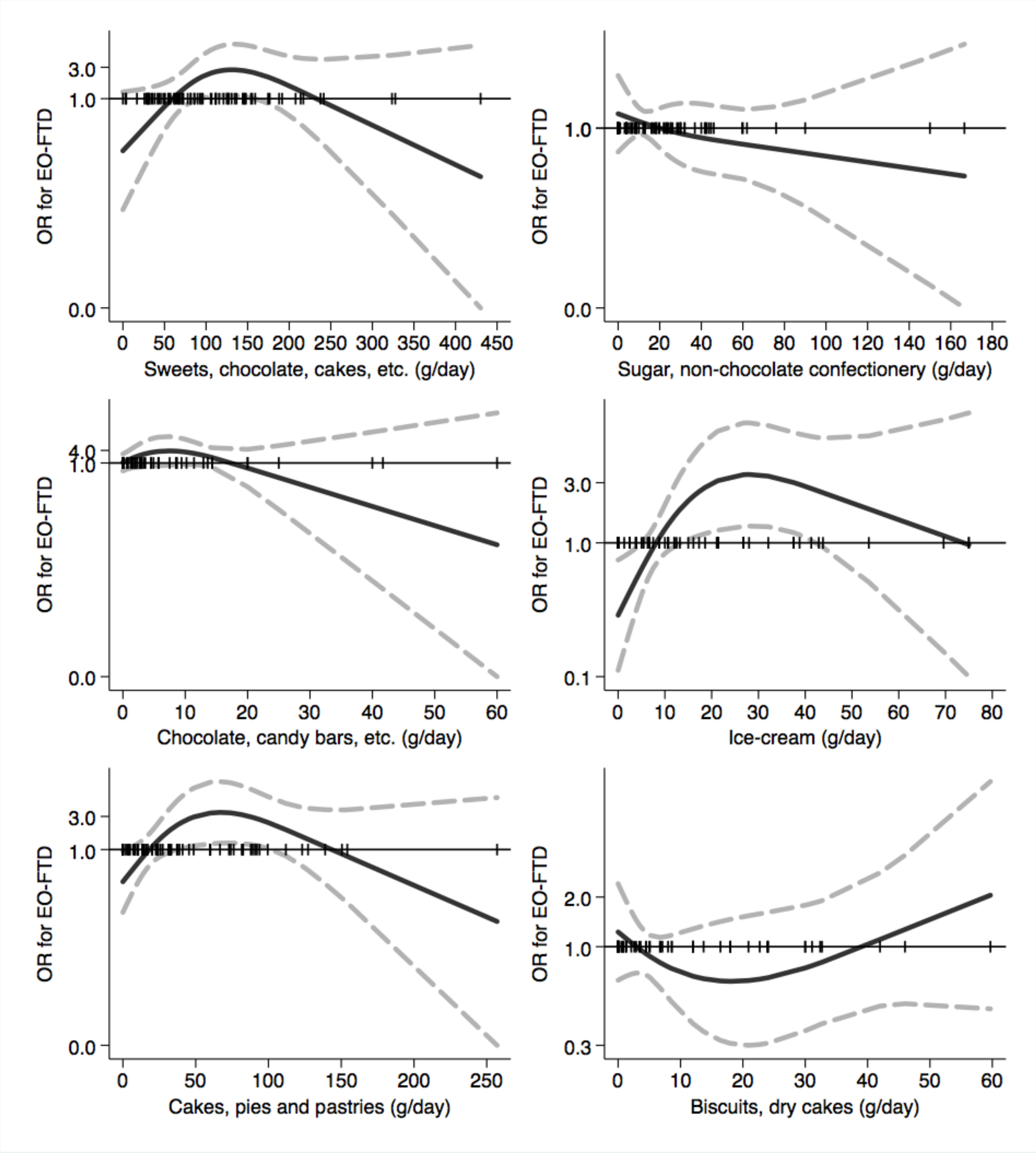

nutrients

Supplemental Figure S19. Oils and fats

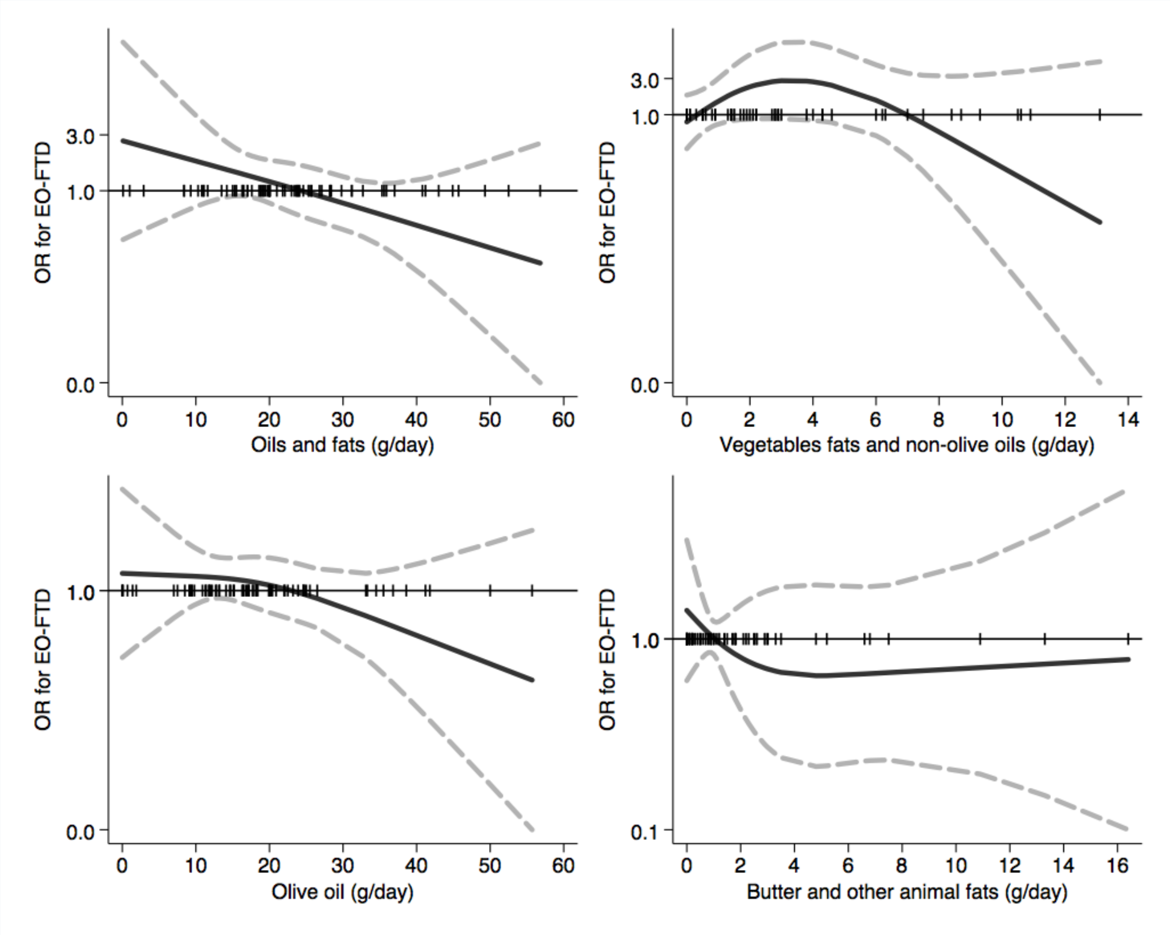

Supplemental Figure S20. Beverages

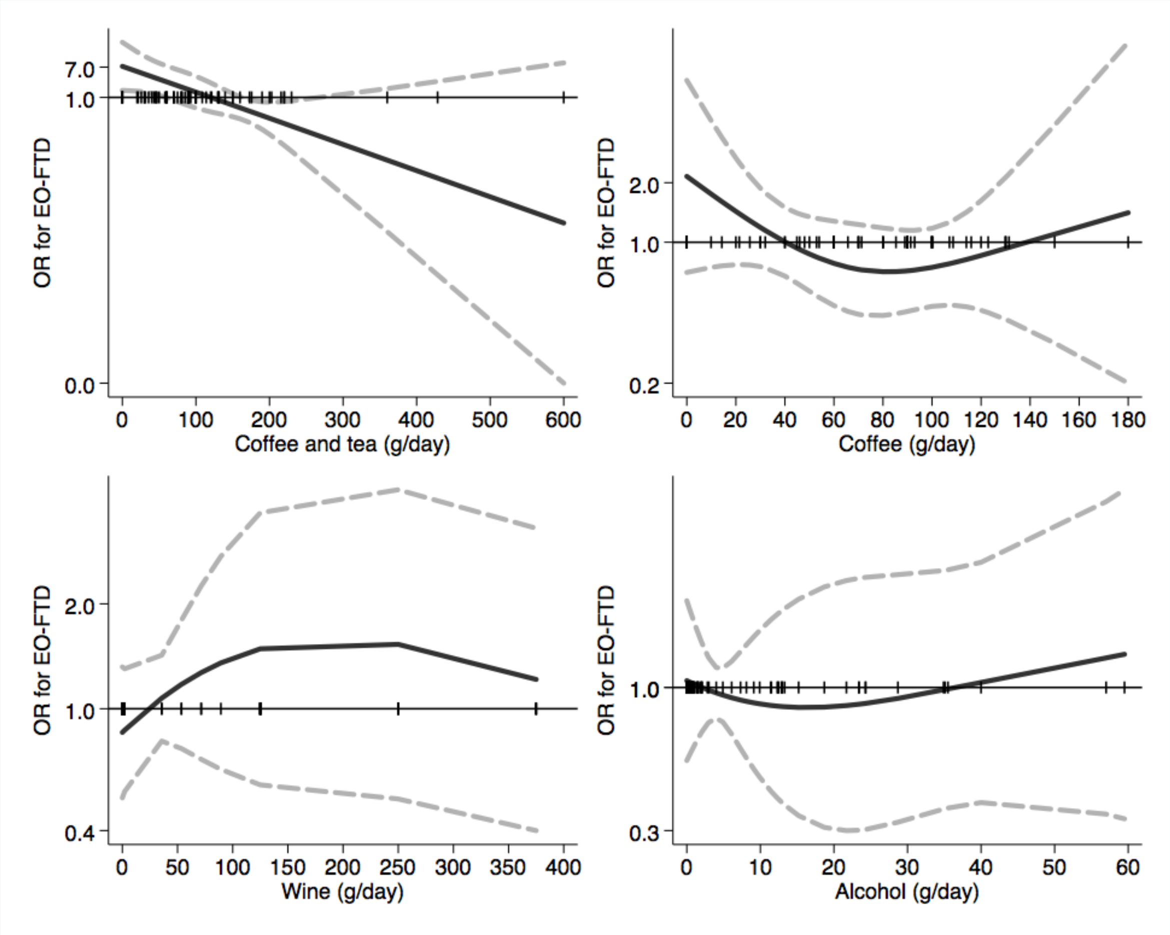

nutrients

MDPI

Supplemental Figure S21. Spline regression analysis of risk of early-onset Alzheimer's dementia (EO-AD) for increasing adherence to the Greek-Mediterranean (GM) diet, Dietary Approaches to Stop Hypertension (DASH) diet, and Mediterranean-DASH Intervention for Neurodegenerative Delay (MIND) diet. The black line indicates the odds ratio for dementia risk; the dash gray lines are 95% confidence limits; the reference line at 1.0 with gray bars shows the distribution of dietary pattern scores.

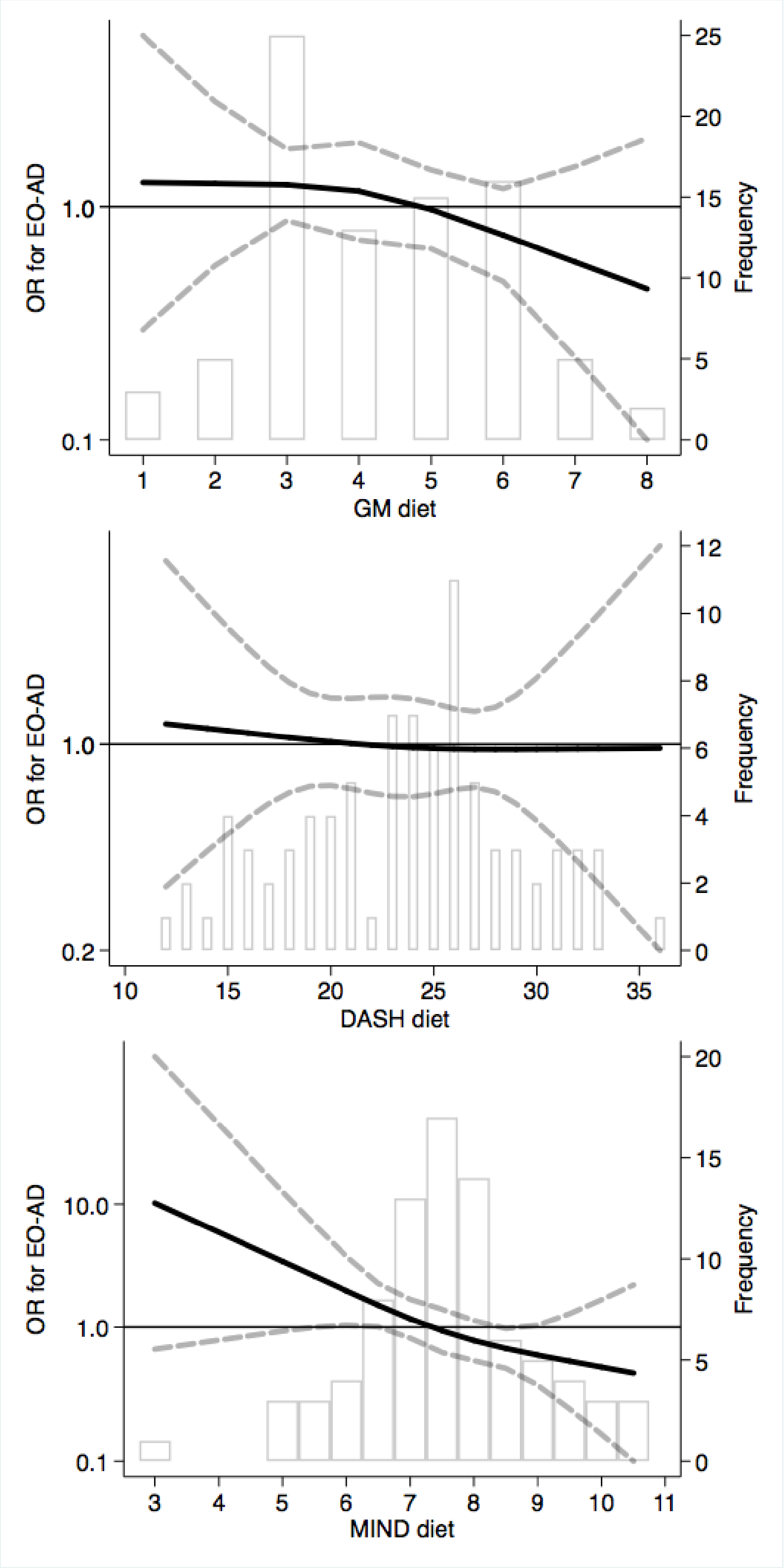

nutrients

MDPI

Supplemental Figure S22. Spline regression analysis of risk of frontotemporal dementia spectrum (EO-FTD) for increasing adherence to dietary patterns. Greek-Mediterranean (GM) diet, Dietary Approaches to Stop Hypertension (DASH) diet, and Mediterranean-DASH Intervention for Neurodegenerative Delay (MIND) diet. The black line indicates the odds ratio for dementia risk; the dash gray lines are 95% confidence limits; the reference line at 1.0 with gray bars shows the distribution of dietary pattern scores.

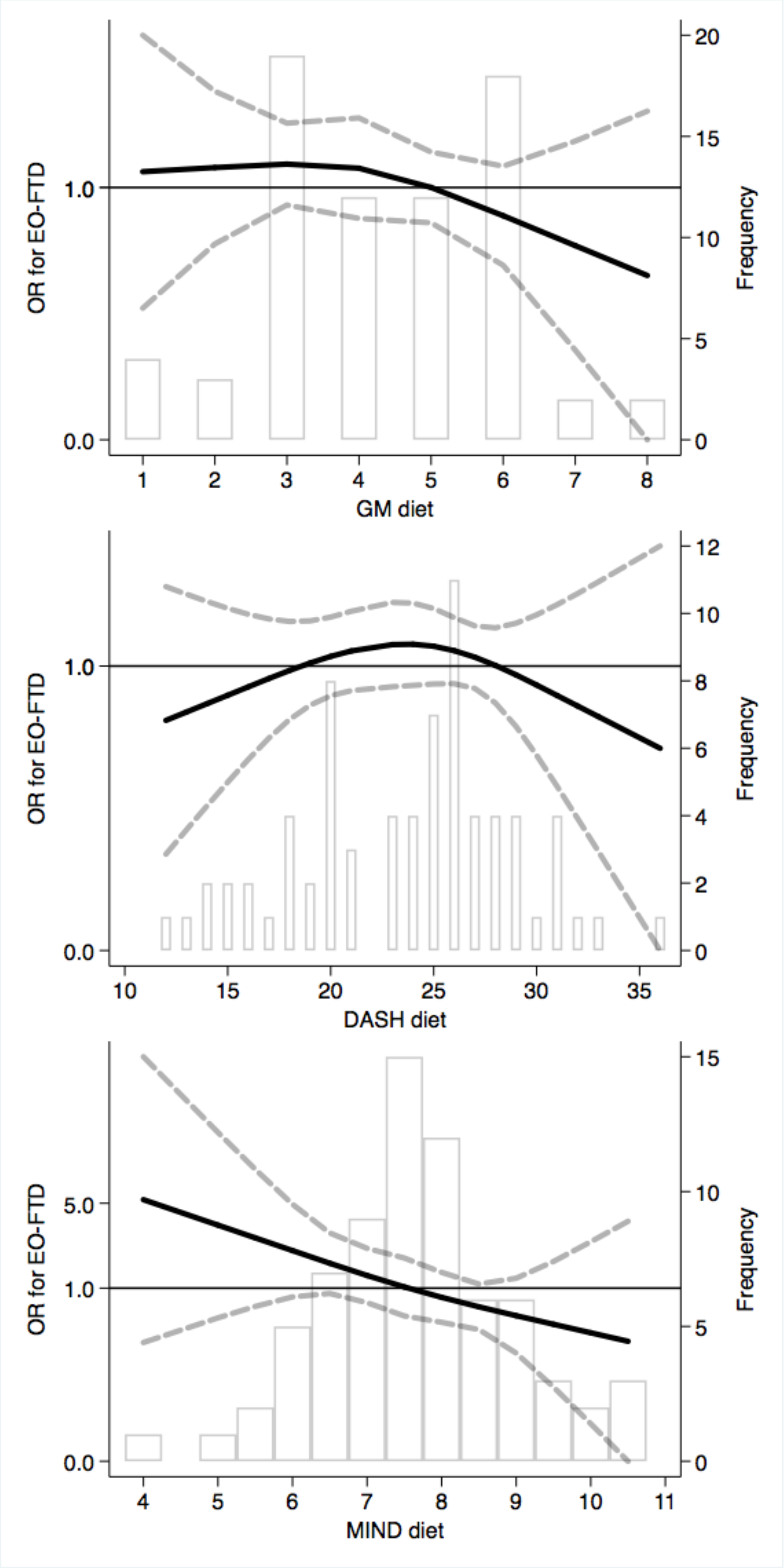

Supplement: Supplementary file 1 [file nutrients-12-03682-s001.pdf]
